# Metabolomics-guided discovery of cytotoxic anthraquinones from the Red Sea-derived fungus *Aspergillus amstelodami* associated with *Sargassum aquifolium*

**DOI:** 10.1039/d6ra07402b

**Published:** 2026-11-03

**Authors:** Ebtisam T. Badawy, Amany E. Ragab, Mohamed A. Farag, Doris Finkelmeier, Anke Burger-Kentischer, Kamilia A. Abo El-Seoud, Ahmed Zayed

**Affiliations:** a Department of Pharmacognosy, Tanta University, College of Pharmacy El-Geish Street (Medical Campus) 31527 Tanta Egypt ahmed.zayed1@pharm.tanta.edu.eg; b Department of Pharmacognosy, Faculty of Pharmacy, Cairo University Kasr El-Aini Street Cairo 11562 Egypt; c Department of Biofabrication and Material Development, Fraunhofer Institute for Interfacial Engineering and Biotechnology Nobelstrasse 12 70569 Stuttgart Germany

## Abstract

Marine macroalgae-associated fungi constitute an underexplored reservoir of structurally diverse secondary metabolites with considerable pharmaceutical potential. To assess the chemical diversity and anticancer potential of fungi associated with *Sargassum aquifolium*, several fungal endophytes were isolated from specimens collected along the Egyptian Red Sea coast. Among the isolated fungi, *Aspergillus amstelodami* AUMC 16413 (Am-10) was prioritized for in-depth chemical and biological investigation based on its metabolite-rich profile in preliminary screening assays. UPLC-HRMS/MS-based metabolomics and GNPS networking enabled the annotation of 53 metabolites (anthraquinones, dihydro-isocoumarins, and prenylated benzaldehydes), revealing an anthraquinone-rich metabolome. Fractionation and spectroscopic analyses led to elucidation of seven anthraquinones, identified as danthron 1, catenarin 2, questin 3A, rubrocristin 3B, questinol 4, dermolutein 5A, and endocrocin 5B. The fungal extract exhibited cytotoxic activity toward MCF-7 cells (CC_50_ = 16.5 ± 1.9 µg mL^−1^; SI = 5.07), with a differential response observed between MCF-7 and normal cells together with alteration in cell-cycle progression and induction of apoptosis. RT-qPCR showed upregulation of *Bax* and *p53*, with downregulation of *Bcl-2*. Among the tested metabolites, compound 1, 3A/3B and 5A/5B mixtures displayed cytotoxic activity, with 5A/5B mixture showing the highest cytotoxic activity and selectivity index among the tested compounds (CC_50_ = 7.37 ± 0.38 µg mL^−1^, SI = 8.88). Molecular docking showed predicted interactions with apoptosis-related targets. Moreover, SwissADME and toxicity predictions indicated predicted ADME properties of the investigated compounds. These findings highlight Red Sea-derived *A. amstelodami* as a source of bioactive anthraquinones warranting further biological investigation.

## Abstract 2

Marine macroalgae-associated fungi constitute an underexplored reservoir of structurally diverse secondary metabolites with considerable pharmaceutical potential.

## Graphical abstract

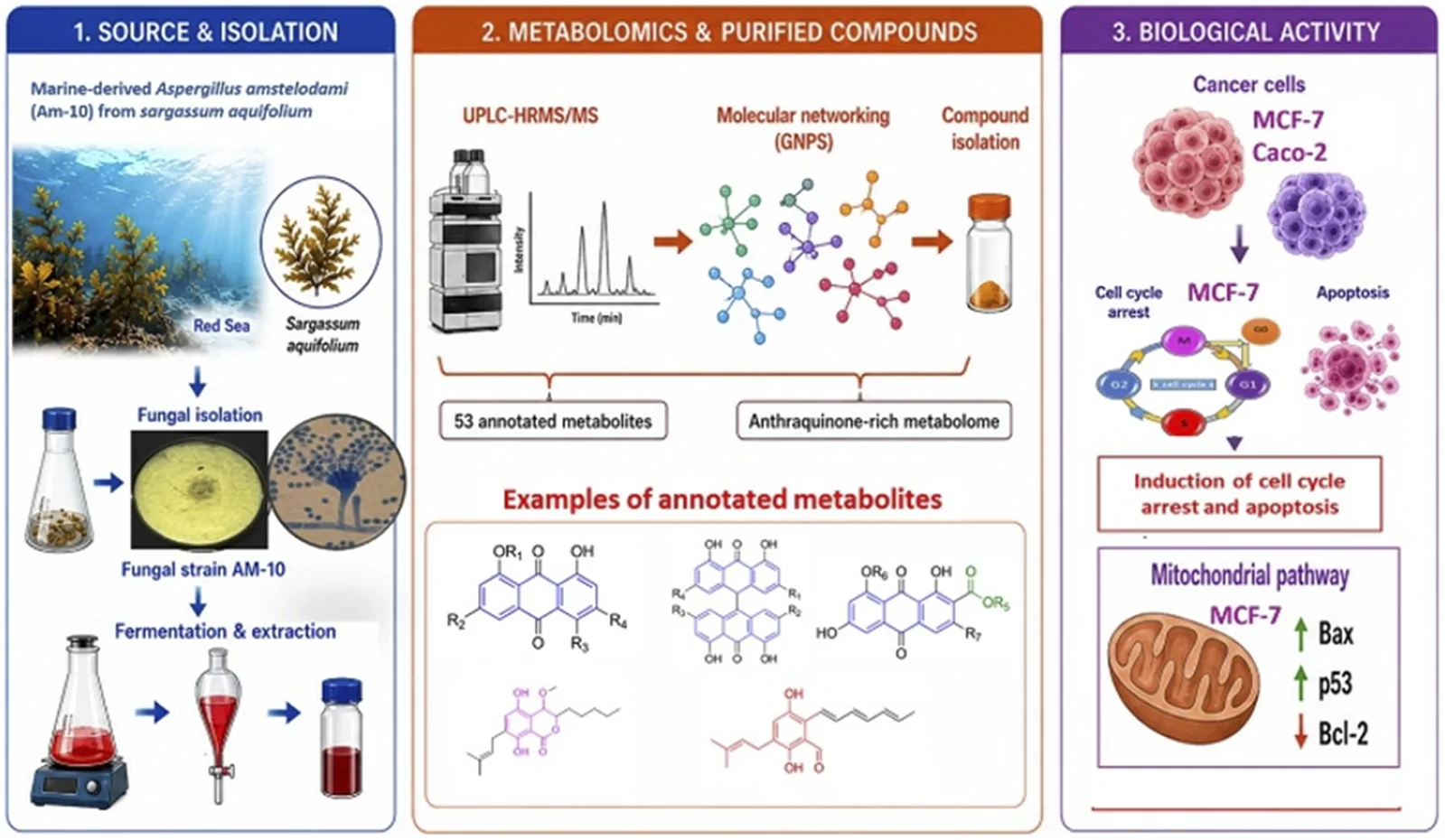

## 1 Introduction

Microorganisms associated with aquatic organisms are an important source of bioactive metabolites with pharmaceutical and industrial applications. Among marine microorganisms, fungi associated with marine macroalgae have attracted considerable attention for their remarkable biosynthetic capacity and adaptation to unique marine environmental conditions.^1^ These fungi represent a prolific yet still underexplored reservoir of structurally diverse natural products with potential applications in drug discovery, particularly in the development of novel anticancer agents.^2^

*Sargassum aquifolium* is a brown macroalga widely distributed in tropical and subtropical marine environments and is known for its diverse biological activities.^3,4^ It also serves as a host for marine-associated microorganisms. However, the fungal communities associated with *S. aquifolium* from the Egyptian Red Sea Coast and their biosynthetic potential remain largely unexplored. This knowledge gap underscores the need for systematic investigations of the fungi associated with them as potential sources of bioactive secondary metabolites.

The genus *Eurotium*, formerly used for the teleomorph state of several *Aspergillus* species, has been taxonomically reassigned to the genus *Aspergillus* under the “one fungus-one name” nomenclature system.^5^ The genus comprises several xerophilic fungi such as *A. amstelodami*, *A. cristatus*, *A. repens*, *A. rubrum*, *A. herbariorum*, and *A. chevalieri*.^6–8^

Several studies have demonstrated that species within the genus *Aspergillus* are rich sources of structurally diverse secondary metabolites with important biological activities.^7,8^ Specifically, *A. amstelodami* has emerged as a promising source due to its ability to produce several biologically active compounds. Previous studies have reported the isolation of anthraquinone derivatives, indole diketopiperazine alkaloids and prenylated benzaldehydes.^7^ These metabolites exhibited significant antioxidant, antimicrobial, and cytotoxic activities,^7,9–11^ highlighting the pharmaceutical and biotechnological importance of *A. amstelodami*.

While anthraquinone production has been extensively investigated in the related species *Aspergillus cristatus*,^8^ comparatively little is known about the anthraquinone profile and biological activities of marine-derived *A. amstelodami* strains. Furthermore, comprehensive metabolomic investigations of marine-derived *A. amstelodami*, especially those associated with marine macroalgae from the Egyptian Red Sea remain scarce. Consequently, the contribution of these strains to the discovery of selective anticancer metabolites is largely unexplored.

Natural products remain an important source of anticancer agents, and numerous fungal secondary metabolites exert their effects through apoptosis induction and cell cycle modulation.^12^ Breast cancer, in particular, remains a major global health burden, highlighting the need to identify new lead compounds with improved selectivity and safety profiles.^13–15^

Therefore, the present study aimed to investigate the metabolomic profile and anticancer potential of a marine-derived *A. amstelodami* strain AUMC 16413 (Am-10) isolated from the Egyptian Red Sea macroalga *S. aquifolium*. The cytotoxic activities of the fungal extract and isolated compounds were evaluated against selected human cancer cell lines, and their selectivity toward normal cells was assessed by calculating the selectivity index. The effects of the extract on apoptosis-related gene expression and cell cycle progression in MCF-7 cells were also assessed. Metabolite profiling was performed using UPLC-HRMS/MS and chromatographic isolation. Molecular docking and ADMET prediction studies were performed to evaluate the binding interactions of selected metabolites with apoptosis-related molecular targets (*Bax*, *Bcl-2*, and *p53*) and predicted their pharmacokinetic properties.

To the best of our knowledge, this is the first integrated study of an Egyptian Red Sea-derived *A. amstelodami* associated with *S. aquifolium*, combining UPLC-HRMS/MS metabolite profiling, anthraquinone purification, cytotoxic and mechanistic evaluation, molecular docking, and ADMET prediction.

## 2 Materials and methods

### 2.1 General procedures

All solvents used for extraction, isolation, and NMR analyses were purchased from E. Merck (Darmstadt, Germany) or Sigma-Aldrich (St. Louis, MO, USA). NMR spectra were recorded in DMSO-d_6_ on Bruker Avance (400 and 100.6 MHz for ^1^H and ^13^C NMR, respectively) or Bruker Ascend (600 and 151 MHz for ^1^H and ^13^C NMR, respectively) spectrometers. Chemical shifts (*δ*) are reported in ppm relative to residual DMSO-*d*_6_ signals (*δ*_H_ 2.50 and *δ*_C_ 39.5). High-resolution ESI-MS data (HRESI-MS) were acquired using a Waters Acquity UPLC-QTOF Premier system. Moreover, analytical LC-MS analyses were conducted on a Waters system equipped with a 2767 autosampler, a 2545 pump, and a Phenomenex Kinetex C18 column (2.6 µm, 100 Å, 4.6 × 100 mm) fitted with a Phenomenex Security Guard precolumn (Luna C5, 300 Å). The mobile phase was delivered at a flow rate of 1.0 mL min^−1^ using a 15 min linear gradient from 10% to 90% acetonitrile (0.045% formic acid) in water (0.05% formic acid). Detection was carried out using a Waters 2998 diode array detector (210–600 nm), a Waters 2424 evaporative light scattering detector (ELSD), and a Waters SQD-2 mass detector operating in both ESI^+^ and ESI^−^ modes (*m*/*z* 100–1000). The compounds isolation was monitored by thin-layer chromatography (TLC) on silica gel G_60_ F_254_ plates using three different solvent systems (SS1–SS3) petroleum ether: ethyl acetate (Pet. ether : EtOAc, 8 : 2), CH_2_Cl_2_ : EtOAc (7 : 3) and CH_2_Cl_2_ : MeOH (8 : 2), respectively.

### 2.2 Macroalgae sample collection, fungal isolation, and fermentation procedures

#### 2.2.1 Macroalgae sample collection

*S. aquifolium* seaweed sample (Fig. S1) was collected from the Red Sea coast at Hurghada, Egypt, located at latitudes 27°17′07.9″ North and longitudes 33°46′24.8″ East. The seaweed was identified and authenticated morphologically by Dr Sarah H. Rashedy, Assistant professor at the National Institute of Oceanography and Fisheries (NIOF), and a voucher specimen (no. 140) was deposited in the Hydrobiology Laboratory herbarium at NIOF.^4,16^

Collected samples (250 g) were thoroughly washed with seawater, sterile distilled water, 5% Tween 80, followed by surface sterilization with 70% ethanol for 1 min to minimize external microbial contamination.^17^

#### 2.2.2 Fungal isolation and culture conditions

The seaweed blades were aseptically cut into small sections (1 × 0.5 cm) using a sterile razor and cultured on saline Sabouraud Dextrose Agar (SDA, Oxoid Ltd, UK) supplemented with chloramphenicol (250 mg L^−1^, Cidocetine^®^, CID, Egypt). Plates were incubated at 28 ± 2 °C for 1–2 weeks, and pure fungal isolates were obtained through repeated subculturing. Pure cultures were maintained on saline SDA slants at 4 °C for further analysis.^17^

#### 2.2.3 Morphological identification

Fungal isolates were cultured on Czapek's yeast extract agar (CYA; Oxoid, UK) and incubated at 28 ± 2 °C, pH 7 for 7–14 days. For *A. amstelodami*, CYA medium was supplemented with 40% sucrose.^18^ Microscopic characteristics were examined by a trinocular Axiostar Plus research microscope at 1000× magnification using lactophenol cotton blue stain. The identified fungal isolates were deposited at Assiut University Mycological Centre as *Aspergillus amstelodami* AUMC 16413 (Am-10, Fig. 1), *A. flavus* AUMC 16405 (Fig. S2-I), and *A. sydowii* AUMC 16417 (Fig. S2-II).

**Fig. 1:**
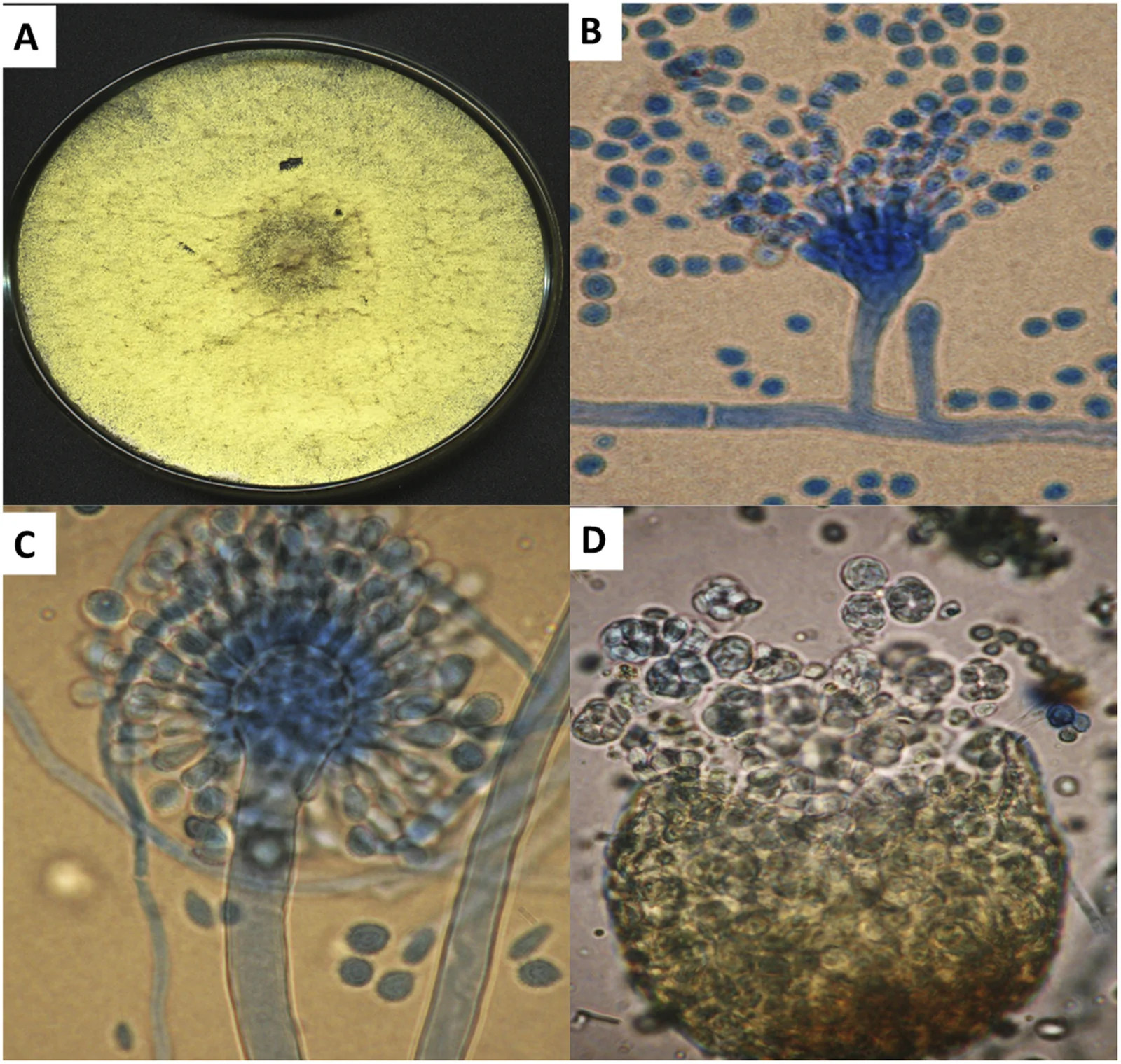
Morphological and microscopic features (1000× magnification) of *A. amstelodami* AUMC 16413 (Am-10), showing yellowish-green colony on 40% sucrose CYA (A) and a conidiophore with young terminal vesicle bearing conidiogenous cells and chains of conidia (B). Conidiophore and conidial head (C) and an ascoma containing asci and ascospores (D).

#### 2.2.4 Preliminary screening of endophytic fungal isolates

A preliminary screening was performed to identify the most promising fungal isolates with potential for secondary metabolite production. The three isolates were cultivated on rice grains (100 g/110 mL distilled water/1 L flask), wheat grains (100 g/110 mL distilled water/1 L flask), and potato dextrose broth (400 mL/1 L flask) at 28 ± 2 °C under static conditions for 21 days.^19^

After the incubation period, cultures were extracted with EtOAc, and the solvent was evaporated under reduced pressure to obtain crude residues. The crude extract weight was determined and expressed as (g) residue per 100 g media (rice and wheat) or per 400 mL culture potato dextrose broth as presented in Table S1. The crude residues were preliminarily analysed by TLC using suitable solvent systems (Fig. S2-III).

#### 2.2.5 Molecular identification

Based on preliminary screening, the most promising fungal isolate was cultured on CYA agar at 28 ± 2 °C for 5 days.^18^ Genomic DNA was extracted using the Patho-gene-spin DNA/RNA extraction kit (Intron Biotechnology, Korea) at the Molecular Biology Research Unit, Assiut University. The internal transcribed spacer (ITS) region was amplified with universal primers ITS1 and ITS4 and sequenced by SolGent (Daejeon, South Korea).^20^ Sequences were identified using BLAST (Basic Local Alignment Search Tool) at the National Centre for Biotechnology Information (NCBI) database, and phylogenetic analysis was performed with MegAlign (DNA Star) version 5.05.

### 2.3 Large-scale fermentation

Large-scale fermentation was conducted using potato dextrose broth based on the results of the preliminary screening. Sterile Erlenmeyer flasks (50, 1 L in size), each containing 400 mL of potato dextrose broth culture, were inoculated and incubated under static, dark conditions at 28 ± 2 °C for 21 days.^21,22^ Culture broth was extracted repeatedly with EtOAc, filtered, and concentrated under reduced pressure to yield the reddish-brown crude fungal extract (4.633 g).

A portion of the extract was reserved for biological and chemical analyses (UPLC-HRMS/MS). The remaining portion (3.590 g) was defatted with petroleum ether and further purified by silica gel column chromatography to isolate secondary metabolites. Yields of isolated compounds or compounds mixtures were calculated as percentages (w/w) relative to the crude total EtOAc extract (3.590 g).

### 2.4 UPLC-HRMS/MS

#### 2.4.1 UPLC-HRMS/MS conditions

For UPLC-HRMS/MS analysis, 200 µL of fungal extract in ethanol (100% v/v) was spiked with umbelliferone (10 µg mL^−1^, Sigma-Aldrich, Taufkirchen, Germany) as an internal standard, mixed with acetonitrile/methanol (1 : 1), and the resulting supernatant was analyzed using an Agilent 6540 UHD Accurate-Mass Q-TOF coupled to a UPLC system (Agilent Technologies). Separation was achieved on Waters Acquity UPLC^®^ CSH C18 column (2.1 × 50 mm, 1.7 µm). The autosampler was kept at 5 °C and the column oven was set to 45 °C. Metabolites were separated using a binary solvent system (A: 0.1% formic acid in water and B: 0.1% formic acid in acetonitrile) running with the following gradient: 0–5 min: 5% B; 5–19 min: 5–95% B; 19–21 min: 100% B; 21–21.5: 100–5% B; and 21.5–24 min: 5% B. Metabolites were eluted at a constant flow rate of 0.3 mL min^−1^. MS/MS data were acquired in negative ion mode (*m*/*z* 60–1600). Blank solvent injections were run between samples.^23–25^

#### 2.4.2 UPLC-HRMS/MS data pre-processing using MZmine 3

Metabolites were annotated following Metabolomics Standards Initiative (MSI) guidelines using MS/MS fragmentation patterns and comparison with mzCloud, KEGG, ChEBI, GNPS, MONA, and the Dictionary of Natural Products databases (Wiley).^23^ Raw UPLC-HRMS/MS data were converted to mzXML using MSConvert (ProteoWizard) and processed in MZmine 3 (v3.9.0) for mass detection. Features with MS/MS spectra within 0–25 min were retained. Metabolites were tentatively annotated by matching library spectra, fragmentation patterns, and previously reported literature data.^26,27^

#### 2.4.3 Molecular networking and compounds dereplication

MS/MS data acquired in negative ionization mode were uploaded as .mgf files to the publicly available Global Natural Product Social Molecular Networking (GNPS) platform (https://gnps.ucsd.edu/) for feature-based molecular networking analysis.^28^ Networks were generated using a parent mass tolerance of 0.05 Da, fragment ion tolerance of 0.05 Da, a cosine score >0.6, and at least four matched peaks. Spectra were matched against GNPS spectral libraries, including NIST13, MassBank, MONA and ReSpect. Molecular networking was performed using GNPS and visualized in Cytoscape (v3.10.4).^29^ Putative annotations were supported by SIRIUS/CSI: FingerID (v 6.3.2).^30,31^

#### 2.4.4 Isolation, purification, and structure elucidation of secondary metabolites

The EtOAc residue of Am-10, obtained after defatting (2.052 g), was dissolved in CH_2_Cl_2_ : MeOH (1 : 1) and loaded onto a silica gel column (65 g, 52 × 1.5 cm, dry method). Elution was carried out by isocratic solvent system of CH_2_Cl_2_ : EtOAc (9.7 : 0.3, Fr. 1–164), then CH_2_Cl_2_ : EtOAc : MeOH (9.5 : 0.5 : 0.3, Fr. 168–250). The collected fractions (10 mL each) were checked by TLC using SS1–SS3 with *R*_f_ values as in Table S2. Similar fractions were concentrated and purified using Sephadex LH-20 (Fig. S3). Compound 1 was isolated from Fr. 5–12 (0.01 g, 0.3% w/w) and compound 2 from Fr. 39–48 (0.008 g, 0.2% w/w). A mixture of 3A/3B was obtained from Fr. 62–68 (0.011 g, 0.3% w/w). Compound 4 was isolated from Fr. 175–180 (0.007 g, 0.19% w/w), while a mixture of 5A/5B was obtained from Fr. 188–190 (0.004 g, 0.11% w/w). The identification and structure elucidation were carried out using various spectroscopic techniques including UV, ^1^H NMR, ^13^C NMR, COSY, HSQC, HMBC, HR-MS and LR-MS spectroscopies.

### 2.5 Cytotoxic activity

#### 2.5.1 Cell lines

Cytotoxic activity was performed using two commercial cancer cell lines MCF-7 (ATCC^®^ HTB-22™) and Caco-2 (ATCC^®^ HTB-37™), derived from human breast and colorectal adenocarcinoma, respectively. They were obtained from LGC Standards GmbH (Wesel, Germany). The study also included normal cell lines of MCF-10A, a non-tumorigenic human mammary epithelial cell line (ATCC^®^ Number: CRL-10317), and FHC, a non-tumorigenic human fetal colon epithelial cell line (ATCC^®^ Number: CRL-1831). The cell lines were routinely tested for mycoplasma contamination using MycoStrip^®^ 2.0 (InvivoGen, cat. no. rep-mysv2-10) and were confirmed to be mycoplasma-free. The cell line identity was verified by the supplier ATCC using STR profiling. The cell lines were maintained according to the supplier's recommendation at low passage numbers to minimize the risk of cross contamination or genetic drift. Given these precautions, no additional STR profiling was performed after receipt of the cells.

#### 2.5.2 Cell culture

Cell culture reagents were purchased from Thermo-Fisher Scientific Inc (Waltham, MA, USA). Cells were cultured in Dulbecco's Modified Eagle medium (DMEM) or Roswell Park Memorial Institute (RPMI-1640) supplemented with 10% fetal calf serum FCS, l-glutamine, and penicillin–streptomycin according to ATCC recommendations.^32^

#### 2.5.3 *In vitro* cytotoxic activity

The cytotoxic activity of the EtOAc extract and isolated compounds from the macroalgal fungal endophyte Am-10 was evaluated using a 96-well fluorescence-based assay according to Zayed *et al.*^32^ Samples were dissolved in DMSO and serially diluted (1250–4.98 µg mL; final DMSO ≤0.1%). Cells (5 × 10^3^ per well) were incubated with the samples at 37 °C and 5% CO_2_. Methotrexate (0.59–300 µg mL^−1^), SDS, and 0.1% DMSO were used as positive, cell death and negative controls, respectively. The tested concentration range of methotrexate was selected based on previously published studies in tumor cell lines.^33,34^ Methotrexate was included as a positive reference control in the cytotoxicity assay. However, because no reliable concentration–response relationship could be established under the experimental conditions used, no CC_50_ value was assigned, and methotrexate was excluded from quantitative comparisons of cytotoxic potency and selectivity.

Cells were exposed to the tested samples for 5 days before FDA measurement. After five days of treatment, the wells were washed with PBS and incubated with FDA (10 µg mL^−1^) for 15 min at room temperature before fluorescence measurement at 485/538 nm. Cell viability was determined by fluorescein diacetate staining (FDA, 10 µg mL^−1^, Sigma-Aldrich^®^), and fluorescence was measured at 485/538 nm. CC_50_ values were calculated from dose–response curves. Three independent biological experiments were conducted (*n* = 3). Data (mean ± SD) were analysed using GraphPad Prism 11.0.2 with two-way ANOVA followed by Tukey's *post hoc* test. The differences were considered statistically significant when *p* < 0.05.

#### 2.5.4 Flow cytometry analysis of cell cycle distribution and apoptosis

For analysis of the effect of Am-10 extract on the cell cycle distribution of the MCF-7 breast cancer cell line, a propidium iodide flow cytometry kit (PI, ab139418, Abcam, Cambridge, UK) was used. Cells were treated with Am-10 EtOAc extract at a concentration of 16.5 µg mL^−1^ for 24 h. After treatment, cells were fixed in 66% cold ethanol at 4 °C for at least 2 h, washed twice with phosphate-buffered saline (PBS), and stained with PI/RNase solution (50 µg per mL PI and 100 µg per mL RNase A) for 30 min at 37 °C in the dark. Samples were analysed using a FACSCalibur flow cytometer equipped with a 488 nm excitation laser. The proportions of cells in G_0_/G_1_, S, and G_2_/M phases were calculated using ModFit LT software v5.0. For detection of apoptosis, an Annexin V apoptosis kit (K101-25, Biovision, Milpitas, CA, USA) was used. Cells were seeded in six-well plates (5 × 10^5^ cells per well) and allowed to adhere overnight before treatment with Am-10 EtOAc extract at a concentration of 16.5 µg mL^−1^ for 24 h, while untreated cells served as the negative control. After treatment, both adherent and floating cells were collected for analysis. Cells were washed twice with cold binding buffer and resuspended in 500 µL of the same buffer. Subsequently, 5 µL Annexin V-FITC (fluorescein isothiocyanate) and 5 µL PI (50 µg mL^−1^) were added to each sample, followed by incubation for 5 min at room temperature in the dark. Stained cells were immediately analyzed using a FACSCalibur flow cytometer (BD Biosciences, San Jose, CA, USA) equipped with a 488 nm excitation laser. FITC and PI fluorescence were detected. The gating strategy was applied sequentially to exclude debris and non-cellular events, followed by analysis of the gated cell population. At least 20 000 events were acquired and analysed by flow cytometry. Data were processed using FlowJo v10 software to discriminate viable (Annexin V^−^/PI^−^), early apoptotic (Annexin V^+^/PI^−^), late apoptotic (Annexin V^+^/PI^+^), and necrotic (Annexin V^−^/PI^+^) cell populations. The percentages of cells in each population were determined from the gated population and used for subsequent analysis.^35,36^

The experiments were performed in three independent biological replicates (*n* = 3). For apoptosis analysis, the total apoptosis percentage for each biological replicate was calculated by summing the percentages of early and late apoptotic cells. The mean and standard deviation (SD) of total apoptosis were calculated from these replicate-level totals. Data are presented as mean ± SD and were analyzed using GraphPad Prism version 11.0.2. Statistical comparisons were performed using two-way ANOVA followed by Tukey's post hoc test, with biological replicates serving as the experimental units. Differences were considered statistically significant at *p* < 0.05.

#### 2.5.5 Effect on apoptotic gene expression

MCF-7 cells were treated with the Am-10 extract (16.5 µg mL^−1^) for 24 h, while control cells received DMSO. Total RNA was extracted using the TRIzol method and reverse transcribed into cDNA with the RevertAid First Strand cDNA Synthesis Kit (Thermo Fisher Scientific, USA). RT-qPCR was performed using SYBR Green Master Mix on a StepOnePlus Real-Time PCR System (Applied Biosystems, Bio-Rad, USA). The expression of apoptosis-related genes *Bax* (*Bcl-2*-associated X protein), *Bcl-2* (B-cell lymphoma 2) and tumor protein *p53* were normalized to glyceraldehyde-3-phosphate dehydrogenase (GAPDH, housekeeping gene) and analysed using the 2^−ΔΔ*C*_*t*_^ method corrected for amplification efficiency (*E* = 1.984). Experiments were performed in triplicate, expressed as mean ± SD, and primer sequences are provided in Table S3.^37,38^

#### 2.5.6 *In silico* molecular docking studies

Molecular docking simulations were performed using Molegro Virtual Docker 2019 v7.0.0 (MVD, accessed April 17th, 2026). The X-ray crystal structures of *Bax* (PDB ID: 4BD6; resolution 2.49 Å), *Bcl-2* (PDB ID: 6O0K; resolution 1.62 Å), and *p53* (PDB ID: 8DC6; resolution 1.60 Å) were obtained from the Research Collaboratory for Structural Bioinformatics Protein Data Bank (RCSB PDB). Protein structures were inspected and prepared in MVD by checking explicit hydrogens, bond orders, hybridization states, atomic charges, and ligand flexible torsions; structural warnings were manually inspected and corrected where necessary. The investigated metabolites were constructed using ChemAxon MarvinSketch, and their lowest-energy conformers were generated using the DREIDING force field and saved as .SDF files before docking.

The binding pockets were defined based on the positions of the corresponding co-crystallized ligands in the experimentally determined structures. The docking search-space centres and radii were: *Bax*, *X* = 1.82, *Y* = −32.98, *Z* = 4.42 Å, sphere radius = 12.4 Å; *Bcl-2*, *X* = −15.29, *Y* = 2.19, *Z* = −11.51 Å, sphere radius = 15 Å; and *p53*, *X* = 40.15, *Y* = −17.21, *Z* = −41.81 Å, sphere radius = 8 Å. Thus, the selected pockets corresponded to experimentally defined ligand-binding regions rather than arbitrarily selected cavities. The MolDock Score (GRID) was used as the scoring function with a grid resolution of 0.30 Å, and the MolDock Simplex Evolution algorithm was employed for the docking search.

Redocking accuracy was assessed by calculating the root-mean-square deviation (RMSD) between the crystallographic co-crystallized ligand poses and the corresponding redocked ligand poses.

For each investigated metabolite, the generated poses were evaluated using the MVD Pose Organizer based on MolDock score and predicted protein–ligand interactions. Docking was performed in 10 independent runs, and the best-scoring pose was selected based on the MolDock score, ligand efficiency and predicted protein–ligand interactions.^39–42^

#### 2.5.7 *In silico* prediction of ADME, toxicity, and drug-likeness

Drug-likeness, pharmacokinetic, and toxicity properties were predicted using SwissADME and pkCSM online tools.^43–45^

### 2.6 Ethics approval

Protocol was approved by Research Ethics Committee, Faculty of Pharmacy, Tanta University, Tanta, Egypt (approval code: TP/RE11/23 Ph-8).

## 3 Results and discussion

### 3.1 Morphological identification

The three fungal strains, isolated from blades of *S. aquifolium*, were morphologically and microscopically identified as *Aspergillus amstelodami* (L. Mangin) Thom and Church (AUMC 16413, Am-10), *Aspergillus flavus* var. columnaris (AUMC 16405), and *Aspergillus sydowii* (Bainier and Saroty) AUMC 16417 (Fig. 1, S2-I and S2-II, respectively). Am-10 appeared as yellowish-green colonies, with long, smooth-walled conidiophores and rough-walled spherical conidia (Fig. 1).

### 3.2 Preliminary screening and selection of the promising isolate

Among the three fungal isolates evaluated, the Am-10 strain exhibited the most promising extract production across the tested fermentation media (Table S1 and Fig. S2-III). It produced yields of 0.156 g crude extract/100 g on rice grain, 0.180 g/100 g on wheat grain, and 0.264 g/400 mL on potato dextrose broth, representing the highest overall crude extract yield. Accordingly, potato dextrose broth was selected as the fermentation medium for large-scale cultivation for Am-10. Based on its superior extract yield and TLC profile, Am-10 was chosen as the most promising fungus for subsequent chemical characterization and biological evaluation.

### 3.3 Molecular identification, phylogenetic analysis, and sequencing results of Am-10

The ITS sequence for Am-10 strain (AUMC 16413, GenBank accession NO: PQ164788) was 553 base pairs, showing 99.49–100% identity with reference strains like *A. amstelodami* NRRL90T (AY373885). The phylogenetic tree constructed for this strain further supported its close relationship with other *Aspergillus* strains (Fig. 2).

**Fig. 2:**
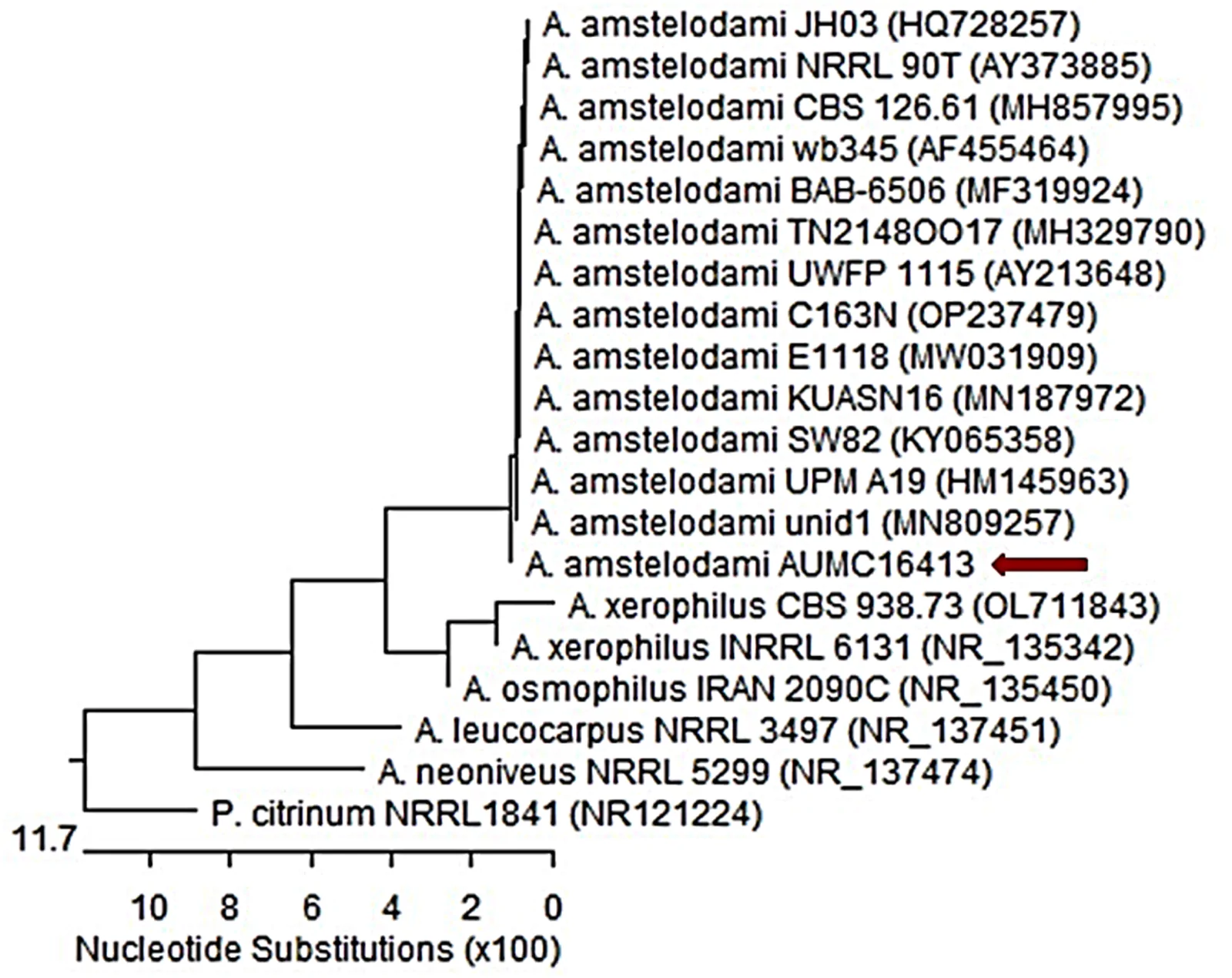
Phylogenetic tree based on ITS sequences of rDNA of the fungal strain *A. amstelodami* AUMC16413 (Am-10, indicated by an arrow) aligned with closely related sequences of the same species accessed from the GenBank. [*A.* = *Aspergillu*s, *P.* = *Penicillium*].

### 3.4 UPLC-HRMS/MS metabolite annotation aided with molecular networking

UPLC-HRMS/MS analysis was performed to profile the metabolites in the EtOAc extract of Am-10. A total of 53 metabolites were tentatively annotated based on accurate mass (error <±5 ppm), MS/MS fragmentation, molecular networking, and comparison with literature and spectral databases (Table S4).^46^ The base peak chromatogram (BPC), metabolite class percentage, chemical structures, and representative MS/MS spectra are shown in Fig. 3-I, 4, S4 and S5–S8, respectively.

**Fig. 3:**
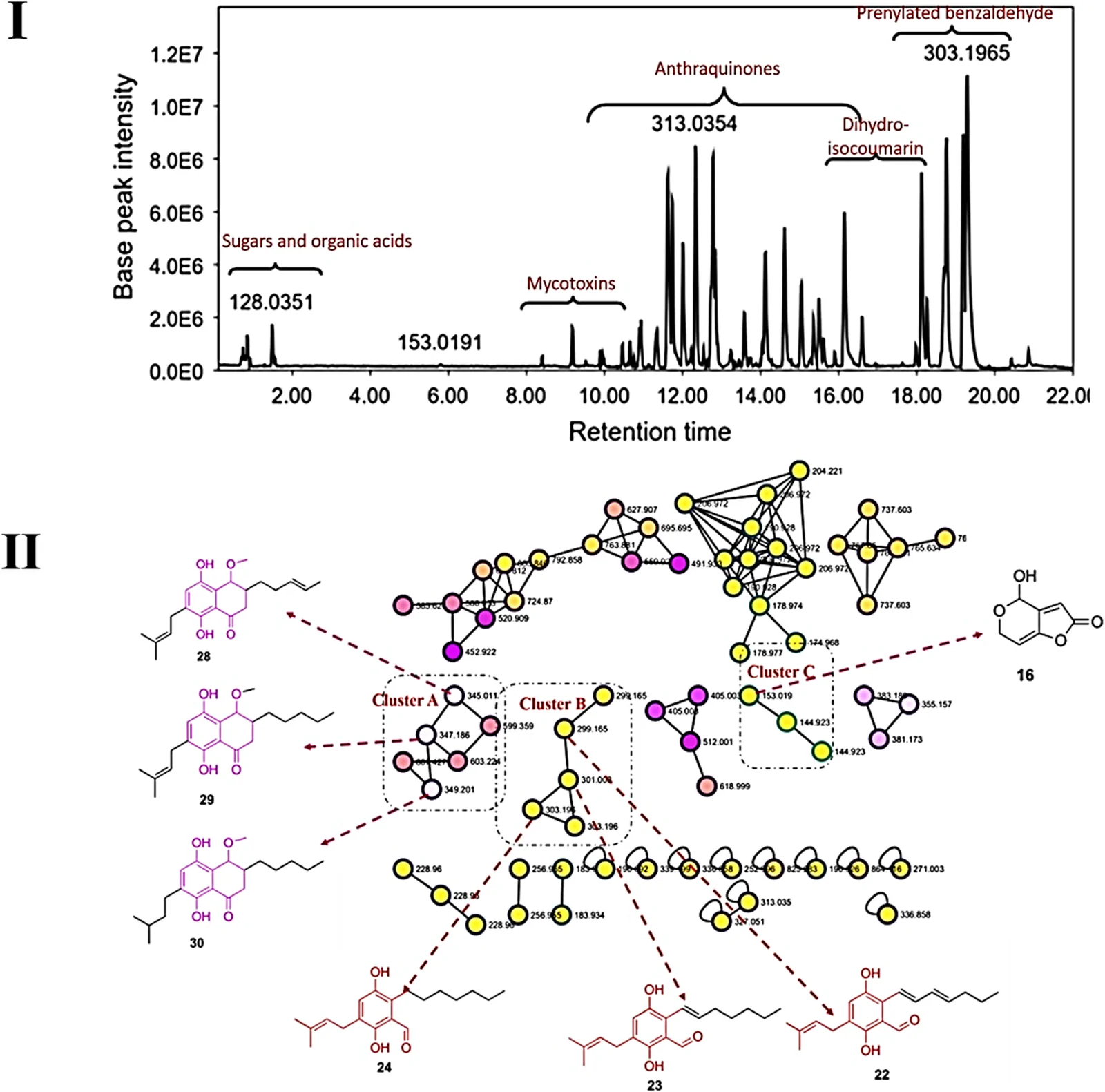
(I) BPC of EtOAc extract of Am-10 as analysed by UPLC-HRMS/MS in negative ion-mode. (II) GNPS feature-based molecular networking (FBMN). Nodes represent precursor ions detected by UPLC-HRMS/MS, while edges indicate spectral similarity relationships based on cosine scores ≥0.6. The figure includes only clustered nodes.

**Fig. 4:**
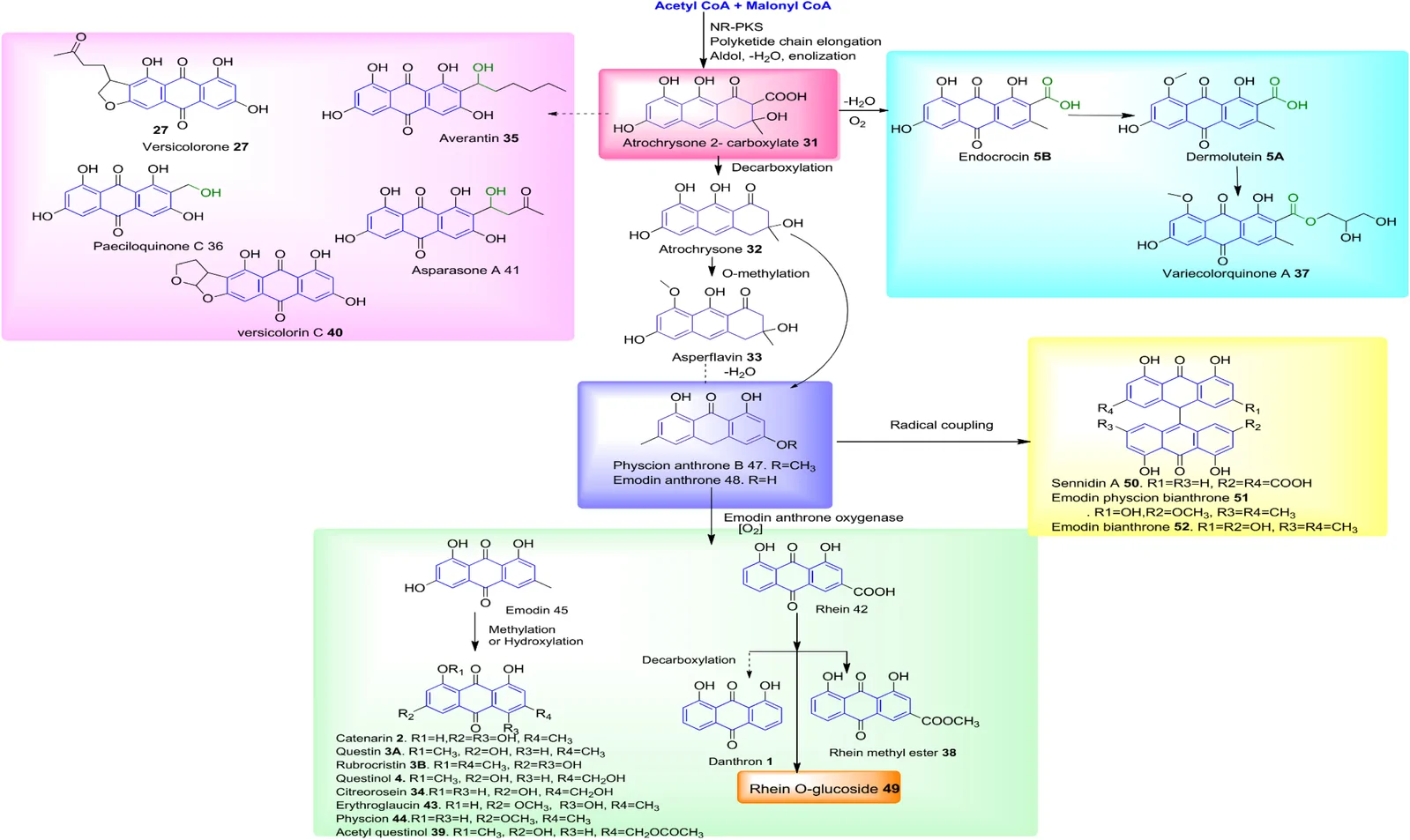
Proposed hypothetical relationships of annotated compounds based on literature and UPLC-HRMS/MS annotation (negative ion mode) of Am-10 EtOAc extract. Solid arrows indicate previously reported biosynthetic pathways of anthraquinones; dashed arrows indicate proposed relationships from LC-MS annotation and literature. Sennidin A is proposed to be derived from rhein anthrone.^49^

Molecular networking (MN) generated 123 nodes, including 11 clusters and 61 singletons, with clusters corresponding to dihydro-isocoumarin polyketides, prenylated benzaldehydes, and mycotoxins (Fig. 3-II). The annotated metabolites comprised 31 anthraquinones, 5 prenylated benzaldehydes, 5 organic/phenolic acids, 3 dihydro-isocoumarin, 3 mycotoxins, 2 lactones, 2 sugars, and 1 indole diketopiperazine alkaloid. The presence of anthraquinones and prenylated benzaldehydes is consistent with the characteristic secondary metabolism of *Aspergillus* species.^8,9^ Notably, dihydroemodin anthrone and dihydroeurotiumide A were tentatively annotated for the first time based on their characteristic MS/MS fragmentation patterns. Several sugars and organic acids were detected at low abundance (Table S4).

#### 3.4.1 Anthraquinones and related derivatives

The structural diversity of fungal anthraquinones presents a considerable challenge for characterization based solely on LC-MS/MS data. Although numerous anthraquinones have been reported from *Aspergillus* species, only a few, including questinol, asperflavin, erythroglaucin, and physcion, have previously been reported from *A. amstelodami*.^8,47,48^

In the present study, anthraquinones constituted the main metabolite class detected in the Am-10 extract (Table S4 and Fig. 4), indicating a broader anthraquinone diversity than previously recognized for *A. amstelodami*. Most anthraquinones appeared as isolated nodes in the negative molecular network because their MS/MS spectra contained only two or three dominant product ions, preventing them from meeting the similarity threshold required for spectral clustering. Subsequently, seven anthraquinones were isolated and structurally characterized by spectroscopic analyses, supporting the UPLC-HRMS/MS-based metabolite annotation.

Early anthraquinones biosynthetic intermediates atrochrysone-2-carboxylate, atrochrysone, and asperflavin were annotated.^49,50^ As illustrated in Fig. 4, these intermediates undergo successive reduction, oxidation, hydroxylation, *O*-methylation, carboxylation, acetylation, glycosylation, and oxidative coupling reactions, giving rise to the structurally diverse anthraquinones detected in the present study.^8,49,50^

Emodin (45) was selected as the representative anthraquinone because it serves as a key biosynthetic precursor of several fungal anthraquinones (Fig. 4). In the negative ion mode, emodin generated the deprotonated ion [M–H]^−^ at *m*/*z* 269.04518 and exhibited characteristic neutral losses of H_2_O, CO, CO_2_, and CH_3_, producing diagnostic fragment ions consistent with previous reports (Fig. 5 and S6).^51,52^ Further cleavage yielded the conserved aromatic fragments at *m*/*z* 178.9775, 112.9850, and 91.0032, which were consistently observed among most annotated anthraquinones, indicating preservation of the anthraquinone aromatic core despite structural modifications.^8,50^

**Fig. 5:**
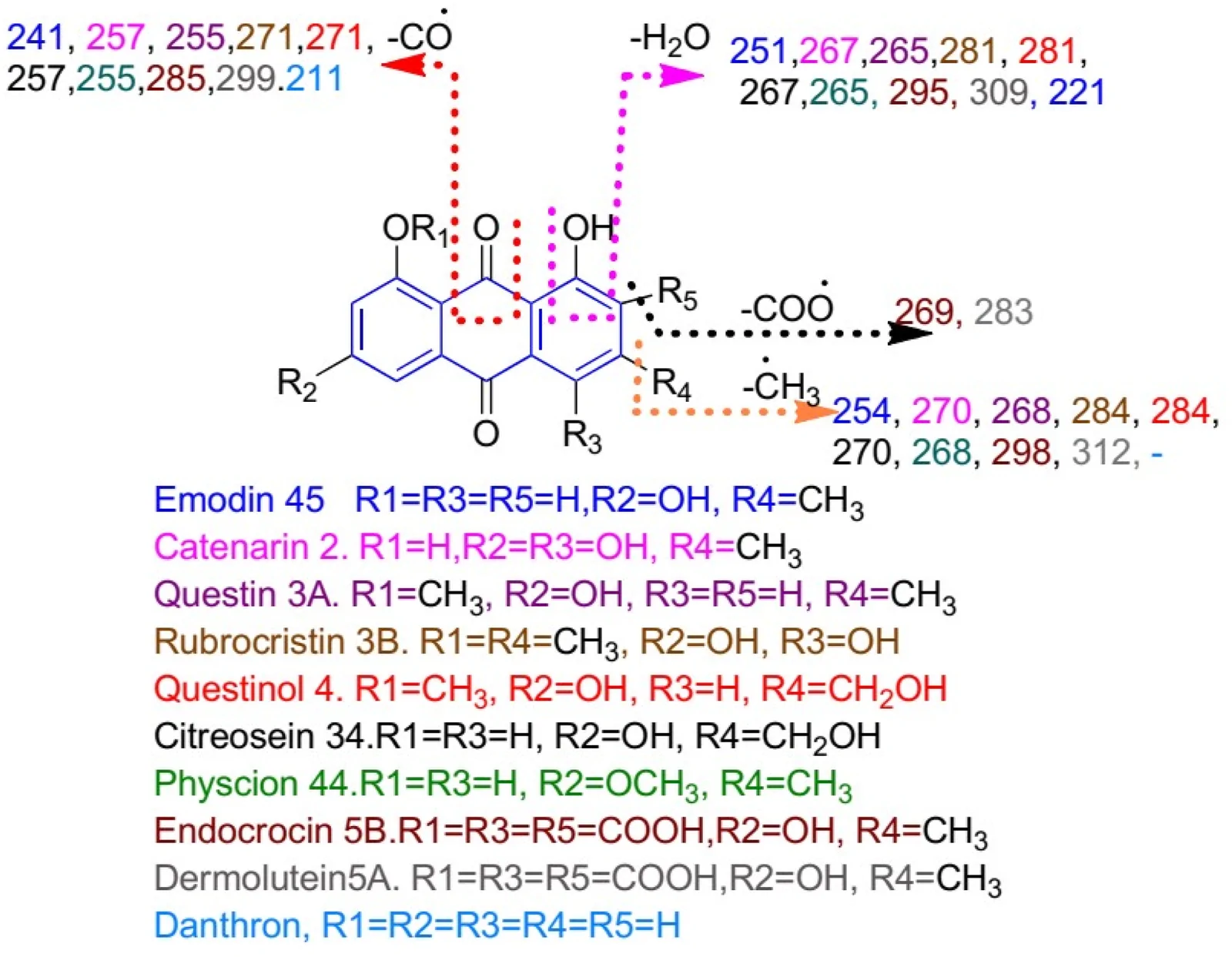
Proposed fragmentation pattern of emodin and some related anthraquinones (negative ion mode).

The anthrone derivatives, including emodin anthrone, dihydroemodin anthrone, and physcion anthrone B, exhibited fragmentation patterns analogous to emodin. Emodin anthrone showed a 14 Da lower precursor ion owing to reduction of one quinone carbonyl, whereas dihydroemodin anthrone displayed an additional 2 Da increase due to hydrogenation of the central ring. Physcion anthrone B retained the same precursor mass as emodin because anthrone formation (−14 Da) was compensated by *O*-methylation (+14 Da), while preserving the characteristic fragmentation pathway.

Substituted anthraquinones were readily distinguished by systematic precursor- and fragment-ion shifts relative to emodin. *O*-Methylated derivatives (*e.g.*, physcion and questin) showed a +14 Da shift, hydroxylated derivatives (*e.g.*, catenarin and citreorosein) showed +16 Da, whereas combined hydroxylation/*O*-methylation (*e.g.*, questinol and rubrocristin) resulted in a +30 Da shift (Fig. 5). Conversely, danthron exhibited a 30 Da decrease due to deoxygenation and demethylation.^8,51^

Highly oxygenated anthraquinones, including endocrocin, dermolutein, acetyl questinol, variecolorquinone A, and asparasone A, exhibited fragmentation patterns consistent with additional carboxyl, methoxy, hydroxyl, or acetyl substituents. Their MS/MS spectra showed the characteristic neutral losses of H_2_O, CO, CO_2_, and acetyl groups, supporting the proposed structures.^8^

The occurrence of glycosylated and dimeric anthraquinones further demonstrated the extensive tailoring capacity of fungal anthraquinone biosynthesis. Rhein *O*-glucoside showed neutral loss of glucose (−162 Da), followed by the conserved anthraquinone fragmentation pathway, whereas the dimeric anthraquinones Sennidin A, emodin–physcion bianthrone, and emodin bianthrone fragmented through cleavage of the dimeric C–C bond to yield characteristic monomeric anthrone ions.

Hence, the annotated anthraquinones shared a conserved emodin-derived fragmentation pattern, with predictable mass shifts reflecting biosynthetic tailoring reactions such as reduction, hydroxylation, *O*-methylation, carboxylation, and glycosylation.^49^ These findings expand the known anthraquinone diversity of *A. amstelodami* and are consistent with previous reports from related *Aspergillus* species.^8^ Notably, several tentatively annotated anthraquinones have not previously been reported from *A. amstelodami*.

#### 3.4.2 Dihydro-isocoumarin polyketide derivatives

Three dihydro-isocoumarin polyketides, including eurotiumide D and eurotiumide A, were tentaively annotated (Table S4, Fig. S5 and S7).^53^ In addition, metabolite 30 was tentatively annotated as a potentially new dihydro derivative of eurotiumide A based on its accurate mass, MS/MS fragmentation pattern, and molecular-network clustering. Structural confirmation by isolation and comprehensive spectroscopic analysis is required. Relative to eurotiumide A, metabolite 30 showed a similar MS/MS fragmentation pattern [M–H_2_O–CO] and [M–H_2_O–CO–CO], with 2 Da difference and close clustering within the GNPS molecular network (Cluster A, Fig. 3-II). It showed coexistence of oxidized and reduced dihydro-isocoumarin derivatives. To the best of our knowledge, these metabolites have not previously been tentatively annotated from *A. amstelodami*.

#### 3.4.3 Prenylated benzaldehyde derivatives

Five prenylated benzaldehydes were tentatively annotated, including flavoglaucin, tetrahydroauroglaucin, dihydroauroglaucin, auroglaucin, and 2-(3,6-dihydroxyhepta-1,4-dien-1-yl)-3,6-dihydroxy-5-dimethylallyl benzaldehyde (Table S4 and Fig. S5). Flavoglaucin was selected as the representative metabolite and exhibited the characteristic fragmentation pattern of this class. It showed a deprotonated molecular ion [M–H]^−^ at *m*/*z* 303.1959, followed by sequential neutral losses of H_2_O and CO to yield fragment ions at *m*/*z* 285.0395 and 275.1975, respectively.

The proposed MS/MS fragmentation tree of flavoglaucin, generated by the SIRIUS software, is presented in Fig. S8. Other related metabolites showed sequential mass losses due to progressive side-chain unsaturation while preserving the conserved benzaldehyde scaffold.^9^ All derivatives clustered within GNPS molecular network Cluster B (Fig. 3-II), supporting their structural annotation. The occurrence of these auroglaucin-related metabolites agrees with previous reports and further confirms their characteristic production by *A. amstelodami*.^9^

#### 3.4.4 Mycotoxins

Three mycotoxins, patulin, aflatoxin G1, and sterigmatocystin, were tentatively annotated in the Am-10 extract (Table S4 and Fig. S5). Patulin clustered within GNPS Cluster C (Fig. 3-II) and exhibited characteristic MS/MS fragmentation pattern. However, these metabolites were detected at low intensity. Importantly, chromatographic purification enables separation of these toxic constituents from the bioactive metabolites.

#### 3.4.5 Indole diketopiperazine (IDP) alkaloids

One IDP alkaloid, neoechinulin A, was tentatively annotated. Neoechinulin A exhibited a precursor ion at *m*/*z* 322.15598 [M–H]^−^ corresponding to the molecular formula C_19_H_21_N_3_O_2_ (Table S4 and Fig. S5). It represented one of non-polyketide metabolites detected in the extract. This is consistent with previous reports from *A. amstelodami*.^11^ The occurrence of this alkaloid alongside abundant polyketides highlights the coexistence of multiple biosynthetic systems within the fungal metabolome.

#### 3.4.6 Lactones

Two lactone metabolites, epiheveadride and oryzalide B, were tentatively annotated (Table S4 and Fig. S5). Epiheveadride has previously been reported from *A. amstelodami*.^10^

#### 3.4.7 Miscellaneous

Other metabolites were tentatively annotated such as Variecoxanthone B, Xanthomonasin B, and Taichunin B (Table S4 and Fig. S5). These compounds belong to different biosynthetic classes, namely xanthones, pyrones, and benzofurans, respectively, and further demonstrate the chemical diversity of the fungal metabolome.

Therefore, the metabolomic profiling was characterized by prominent annotation of polyketide-derived secondary metabolites using potato dextrose broth culture media. These findings are consistent with metabolite patterns previously observed in related fungi.^8^

### 3.5 Structure elucidation of the isolated compounds

Spectroscopic analyses enabled the structural elucidation of seven anthraquinones. 3A/3B and 5A/5B were each obtained as mixtures (each of two compounds). The metabolites were identified as danthron (1), catenarin (2), questin (3A), rubrocristin (3B), questinol (4), endocrocin (5B), and dermolutein (5A) using UV, ^1^H NMR, ^13^C NMR, COSY, HSQC, HMBC, and HRESI-MS spectroscopic analyses (Tables S5 and S6). Their chemical structures and key HMBC correlations are shown in Fig. 6. Detailed spectroscopic data and comparisons with the published literature are provided in the SI.

**Fig. 6:**
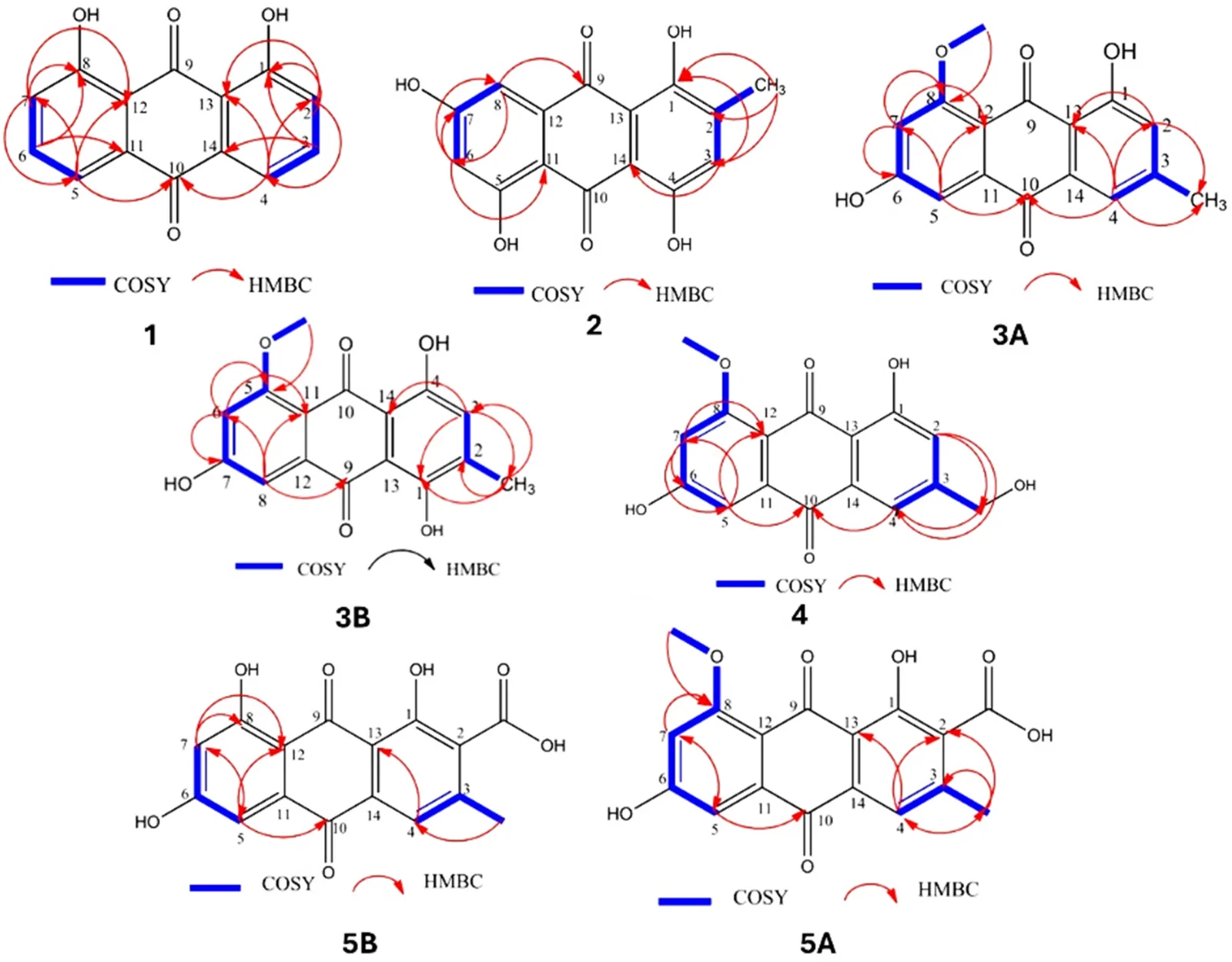
Chemical structures of compounds 1, 2, 3A, 3B, 4, 5A, and 5B showing HMBC (red arrows) and COSY (bold blue) correlations.

The relative chromatographic purity of the isolated compounds was estimated from photodiode array (PDA) chromatograms using the peak-area normalization method. The relative area percentage was calculated by dividing the integrated area of the peak assigned to the target compound by the sum of the integrated areas of all detected chromatographic peaks at the same detection wavelength and multiplying by 100. The estimated relative chromatographic purities were 88.00%, 80.89%, and 85.70% for compounds 1, 2, and 4, respectively. For the mixtures containing compounds 3A/3B and 5A/5B, the corresponding relative peak-area percentages were 58.15% and 41.85%, and 13.38% and 86.62%, respectively. For compounds evaluated as mixtures (3A/3B and 5A/5B), the cytotoxicity results reflect the tested mixtures and cannot be attributed independently to each component. We acknowledge that the relative chromatographic area percentages do not establish absolute chemical purity.

These values represent relative chromatographic area percentages under the applied analytical conditions rather than absolute chemical purity, because differences in UV absorptivity among components and the possible presence of co-eluting or undetected impurities may affect the estimates. Accordingly, the cytotoxicity results should be interpreted in the context of the estimated relative chromatographic compositions, and a contribution from minor impurities cannot be completely excluded.

### 3.6 Cytotoxic activity

#### 3.6.1 *In vitro* cytotoxic activity of total crude extract

The cytotoxic activity of the Am-10 EtOAc extract was evaluated against MCF-7 breast cancer and Caco-2 colon cancer cell lines using the FDA assay. Selectivity was assessed using MCF-10A and FHC normal cell lines. As the Am-10 extract showed the highest cytotoxicity and selectivity against MCF-7 breast cancer cells, the cytotoxicity of the isolated compounds was assessed against MCF-7 breast cancer cells, and the selectivity of the most active compounds was evaluated using normal breast (MCF-10A) cells.

The resulting CC_50_ values and selectivity indices are summarized in Table 1 and Fig. 7. The representative microscopic images are shown in Fig. 8, and S52. Data are presented as mean ± SD (*n* = 3), and the differences were significant (*p* < 0.0001).

**Table 1:** Cytotoxicity (CC_50_, µg mL^−1^) and selectivity indices (SI) of extracts and isolated compounds from endophytic fungi (Am-10) against cancerous and normal cell lines

CC_50_ (µg mL^−1^ ± SD)^a^						
Sample	MCF-7	MCF-10A	SI	Caco-2	FHC	SI
Am-10 extract	16.5 ± 1.9	83.59 ± 3.18	5.07	58.3 ± 6.4	55.07 ± 2.10	0.94
Compound 1	12.38 ± 0.44	40.43 ± 1.98	3.27	—	—	—
Compound 2	19.85 ± 0.65	—	—	—	—	—
Mixture 3A/3B	17.81 ± 0.84	36.82 ± 2.31	2.07	—	—	—
Compound 4	26.12 ± 1.73	—	—	—	—	—
Mixture 5A/5B	7.37 ± 0.38	65.46 ± 3.28	8.88	—	—	—
Methotrexate	ND*	ND*	—	ND*	ND*	—

a Data are presented as mean ± SD (*n* = 3). CC_50_, concentration causing 50% loss of cell viability; SI, selectivity index (CC_50_ normal cells/CC_50_ cancer cells); ND*, methotrexate CC_50_ could not be determined within the tested concentration range.

**Fig. 7:**
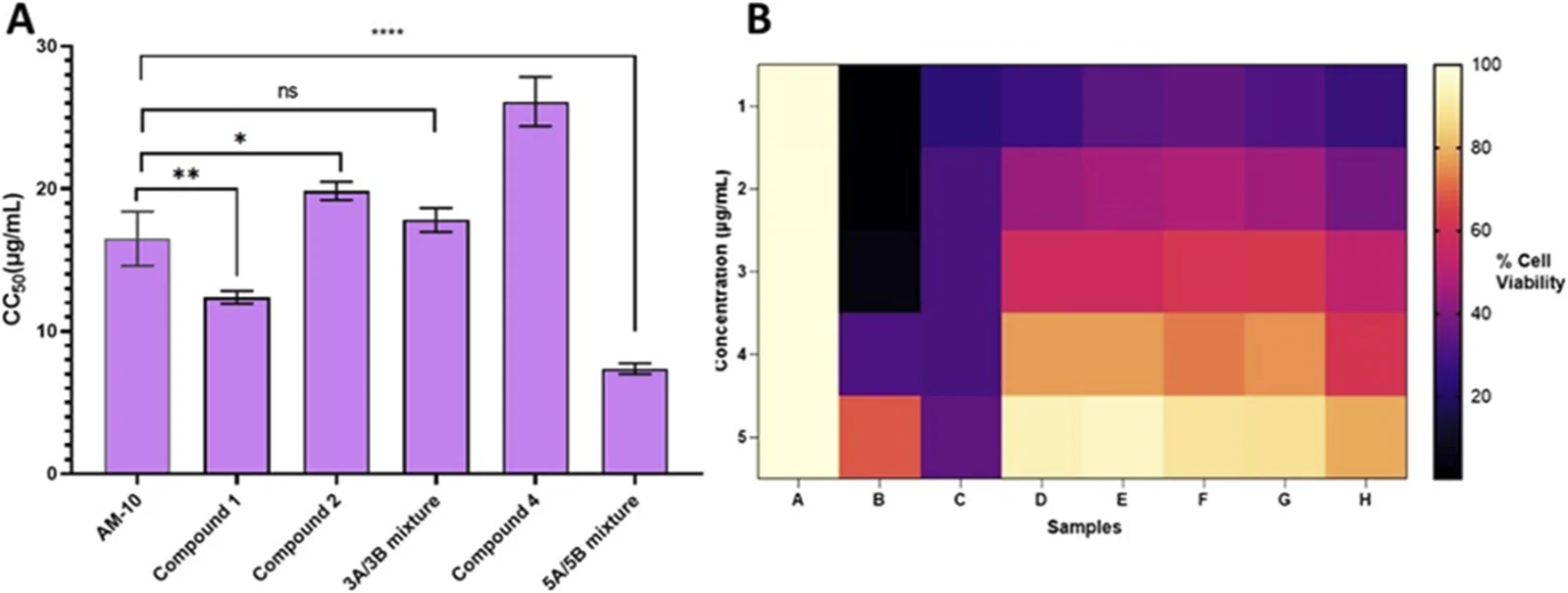
Cytotoxic effects of Am-10, methotrexate, and compounds 1, 2, 4, and the 3A/3B and 5A/5B mixtures on MCF-7 breast cancer cells. (A) CC_50_ values (µg mL^−1^) of Am-10 and isolated compounds are presented as mean ± SD with statistical significance (**** = *p* < 0.0001; ns = not significant). (B) Heatmap showing the percentage of cell viability after treatment with serially diluted samples: methotrexate (300 µg mL^−1^), Am-10 (1250 µg mL^−1^), and isolated compounds (100 µg mL^−1^). A = negative control, B = Am-10, C = methotrexate, and D–H = compounds 1–5, respectively.

**Fig. 8:**
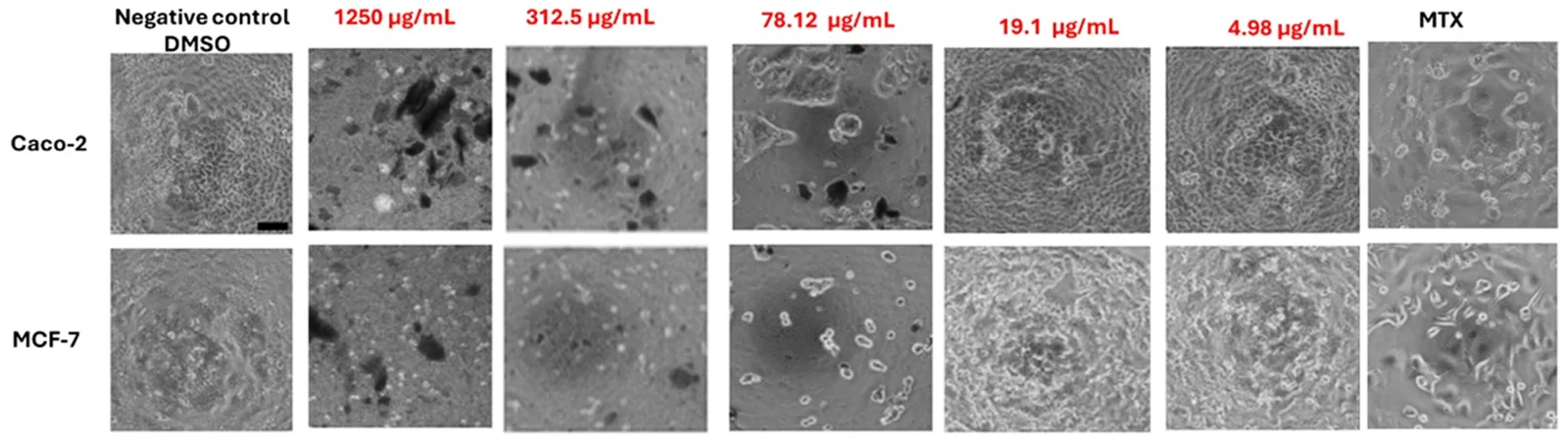
Microscopic visualization of cytotoxicity induced by Am-10 EtOAc extract on Caco-2, MCF-7 at different concentrations (µg mL^−1^), magnification power (*X* = 100).

The results presented in Table 1 and Fig. 7 and 8 revealed that, Am-10 exhibited concentration-dependent cytotoxicity with CC_50_ values of 16.5 ± 1.9 and 58.3 ± 6.4 µg mL^−1^ against MCF-7 and Caco-2 cells, respectively.^54^ Methotrexate (MTX) was tested over a broad concentration range (Fig. 7 and S52-III). However, cell viability remained relatively low across the tested concentrations, with no clear concentration-dependent response. As a reliable concentration–response relationship could not be established, a reliable CC_50_ value could not be determined. Therefore, methotrexate was used only as a reference positive control and was not included in quantitative potency or selectivity comparisons with the isolated metabolites.

Moreover, cytotoxicity of Am-10 extract was assessed using normal breast MCF-10A and normal colon FHC cell lines to determine its selectivity (Table 1 and Fig. S52-II). The results revealed that Am-10 extract showed selectivity indices of 5.07 and 0.94 for breast cancer MCF-7 and colon cancer Caco-2, respectively. SI values greater than 2 are generally considered selective for cancer cells.^55^ Therefore, Am-10 showed greater selectivity for breast cancer than for Caco-2 colon cancer cells.

These findings are consistent with previously reported cytotoxic activity of anthraquinones and the reported anticancer potential of *A. amstelodami* and related *Aspergillus* species.^56–58^ Although the cytotoxicity of the terrestrial *A. amstelodami* extracts against MCF-7 cells has been previously reported,^59^ the underlying mechanism remained unexplored. Based on the observed cytotoxic activity and selectivity index of Am-10 toward MCF-7 breast cancer cell line, further research, including mechanistic studies and metabolomic investigation, was necessary to explore the potential pathways of cytotoxicity.

#### 3.6.2 *In vitro* cytotoxic activity of isolated metabolites

The cytotoxic activity of the isolated metabolites was evaluated against the MCF-7 breast cancer cell line, and the SI of the most active metabolites were determined. As shown in Table 1, the 5A/5B mixture, compound 1, the 3A/3B mixture, compound 2, and compound 4 exhibited CC_50_ values of 7.37 ± 0.38, 12.38 ± 0.44, 17.81 ± 0.84, 19.85 ± 0.65, and 26.12 ± 1.73 µg mL^−1^, respectively. The 5A/5B mixture exhibited the highest SI (8.88), indicating cytotoxicity toward MCF-7 cells with comparatively lower toxicity toward normal mammary cells. Because compounds 5A and 5B were evaluated as a mixture, their individual biological activities could not be established. Methotrexate was included as a reference positive control; however, a reliable concentration–response relationship and CC_50_ value could not be established under the tested conditions. Therefore, methotrexate was not used for quantitative potency or selectivity comparisons with the isolated metabolites.

The observed activity of the 5A/5B mixture is consistent with previous reports of cytotoxic effects associated with carboxylated anthraquinones. However, the contribution of dermolutein or endocrocin individually cannot be determined from the present mixture.^56,60,61^

Based on their observed cytotoxic activity and selectivity indices under the tested conditions, the isolated compounds were subsequently subjected to molecular docking and *in silico* ADMET analyses to further explore their potential molecular interactions and predicted physicochemical and ADME properties.

#### 3.6.3 Effect of Am-10 on cell cycle distribution and apoptosis

In the present study, the effect of Am-10 extract on cell-cycle distribution and apoptosis of MCF-7 breast cancer cells was analysed by flow cytometry using propidium iodide and annexin V-FITC, respectively. The results obtained are presented in Table S7 and Fig. 9. In addition, statistical analysis of the cell-cycle populations has been provided in Table S7. Compared with untreated MCF-7 cells, Am-10 treatment significantly increased the G_0_/G_1_ population (61.22 ± 5.51% *vs.* 52.94 ± 3.18%, *p* = 0.0171) and S-phase population (36.92 ± 2.95% *vs.* 28.11 ± 1.97%, *p* = 0.0115), while significantly decreasing the G_2_/M population (1.86 ± 0.09% *vs.* 18.95 ± 1.33%, *p* < 0.0001). These results support an alteration in cell-cycle distribution following Am-10 treatment.

**Fig. 9:**
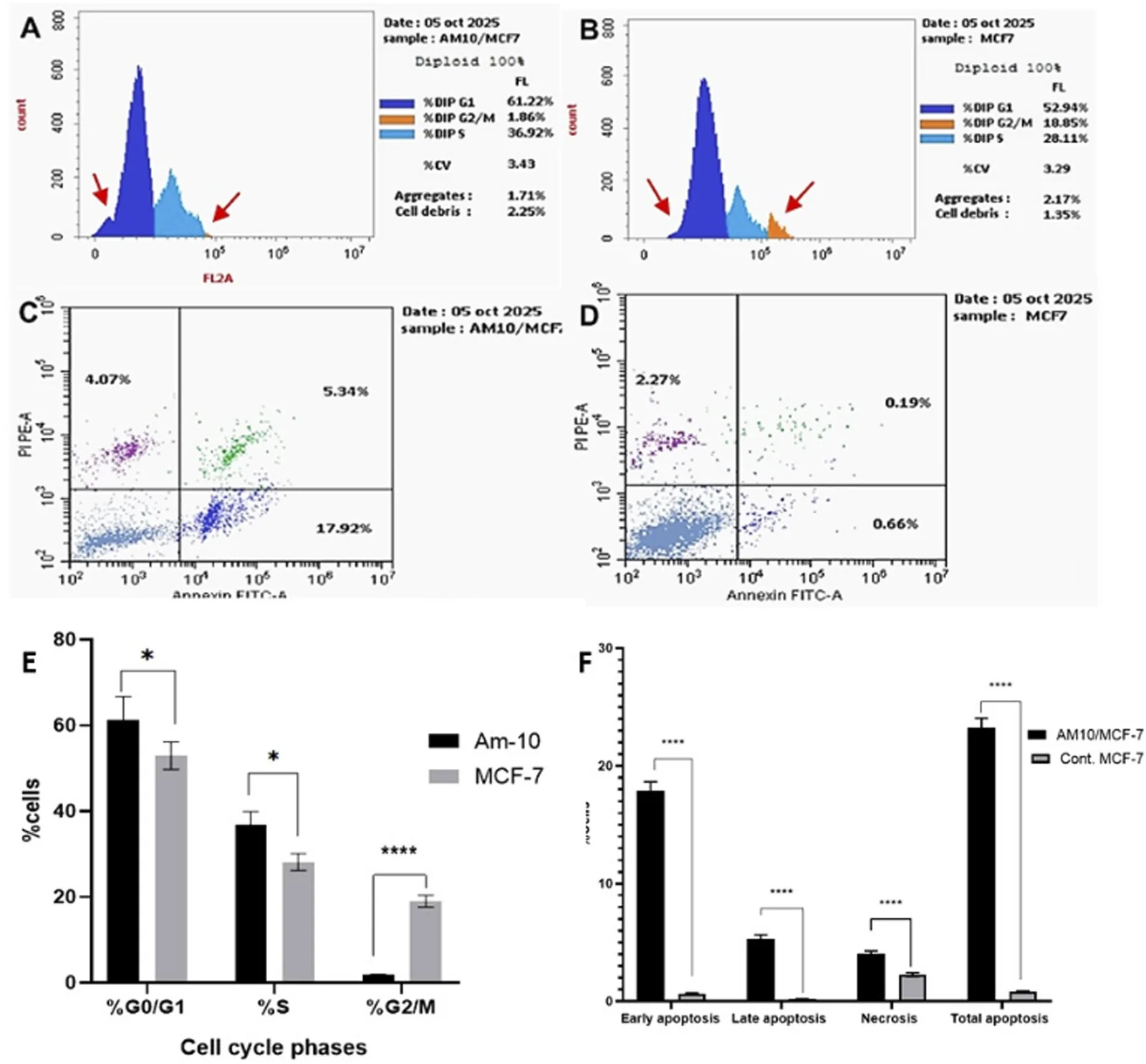
Effect of Am-10 on cell cycle and apoptosis of MCF-7 breast cancer cell line. (A) Effect of Am-10 on the cell cycle of MCF-7. (B) The cell cycle of untreated MCF-7 cells was used as a negative control. (C) Apoptosis induced by Am-10 using the MCF-7 breast cancer cell line. (D) Apoptosis in untreated MCF-7 cells as a negative control. The four quadrants in C and D are identified as: lower left (viable); lower right (early apoptotic); upper right (late apoptotic); upper left (necrotic). (E) Statistical analysis of effect of Am-10 on MCF-7 cell cycle. (F) Statistical analysis of effect of Am-10 on apoptosis of MCF-7.

Apoptosis was assessed using Annexin V-FITC/PI dual staining (Fig. 9 and Table S7). Am-10 markedly increased total apoptotic cells to 23.26 ± 1.04%, including early apoptosis (17.92% ± 0.72%) and late apoptosis (5.34% ± 0.32%), compared with 0.85 ± 0.04% total apoptosis in control. Necrosis increased only slightly (4.07 ± 0.20% *vs.* 2.27 ± 0.16%), indicating that apoptosis was the predominant mode of cell death.

The increase in early apoptotic cells, together with alteration in cell-cycle distribution, may suggest the involvement of apoptosis-related pathways, potentially associated with apoptotic factors such as *Bax*, *Bcl-2* and *p53*. These findings provide an indication of possible involvement of apoptosis-related signalling; however, further molecular and mechanistic studies are required to confirm this hypothesis. These findings are in good agreement with previously reported data on the effects of anthraquinone derivatives on cell cycle and apoptosis.^62^ To the best of our knowledge, this is the first report of *A. amstelodami* effect on cell cycle progression and apoptosis.

#### 3.6.4 Effect on apoptotic gene expression

The effect of Am-10 on apoptosis-related gene expression was evaluated by RT-qPCR. Am-10 significantly modulated the expression of key apoptotic regulators in MCF-7 cells by markedly upregulating the pro-apoptotic genes B-cell lymphoma 2-associated X protein (*Bax*; 7.92 ± 0.06-fold) and tumor protein *p53* (p53; 3.88 ± 0.21-fold), while downregulating the anti-apoptotic gene B-cell lymphoma 2 (*Bcl-2*; 0.43 ± 0.007-fold). Consequently, the *Bax*/*Bcl-2* ratio increased 18.52-fold relative to the untreated cells, suggesting increased expression of proapoptotic genes.

These findings are consistent with previous reports linking fungal anthraquinones with modulation of apoptosis-related pathways; however, the specific molecular pathway responsible for the effects observed here remains to be established.^63^

#### 3.6.5 *In silico* molecular docking simulation of isolated compounds

Molecular docking was performed to investigate the predicted interaction of the isolated compounds with the apoptosis-related proteins *Bax* (PDB ID: 4BD6), *Bcl-2* (PDB ID: 6O0K), and *p53* (PDB ID: 8DC6) using Molegro Virtual Docker (Fig. 10, S53 and Table S8).

**Fig. 10:**
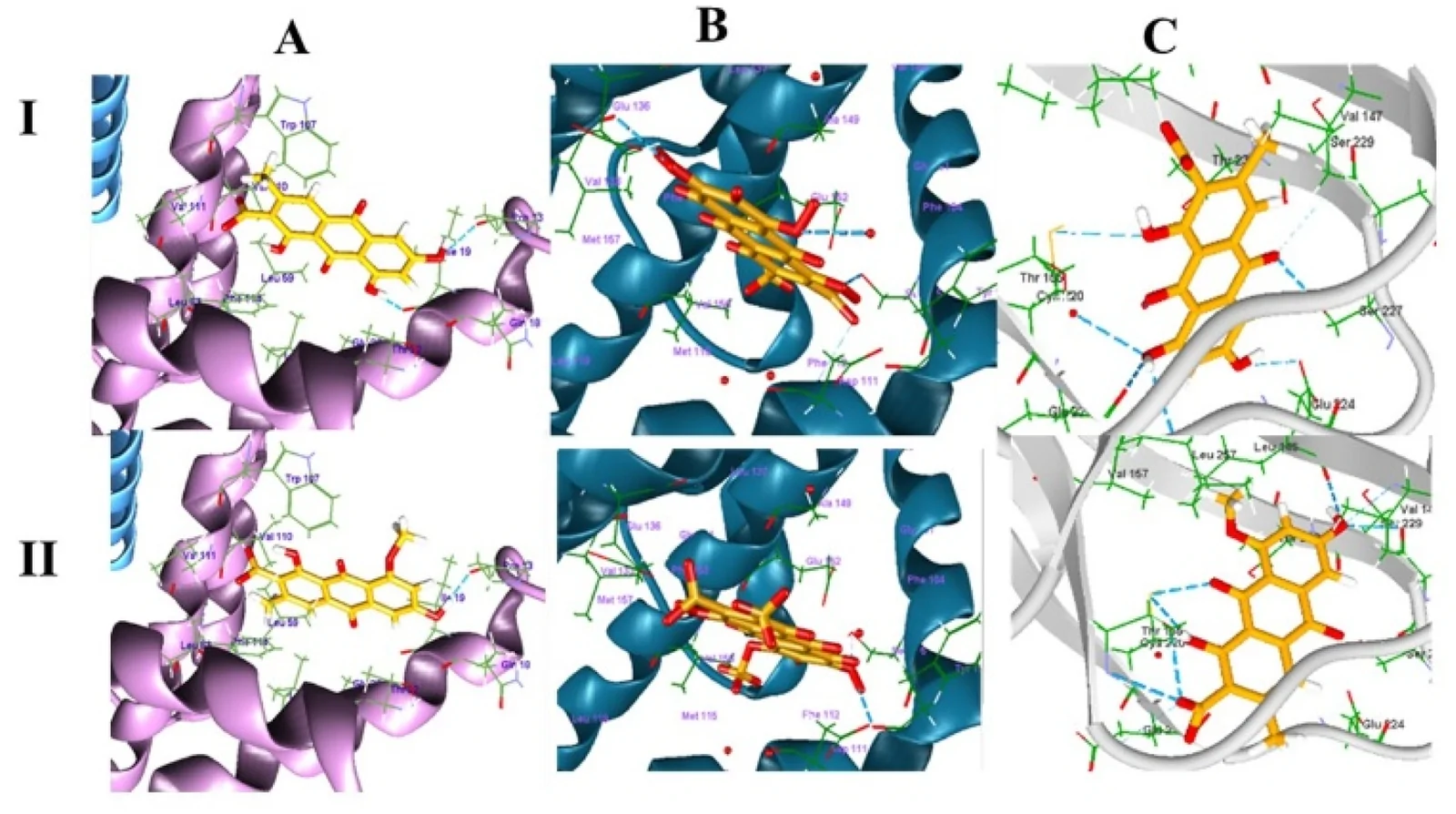
Molecular docking simulation of compounds (I) 5B and (II) 5A to apoptosis-related targets (A) *Bax*, (B) *Bcl-2*, and (C) *p53*.

Molecular docking showed predicted binding of all compounds toward *Bax*, *Bcl-2*, and *p53* through multiple hydrogen-bond interactions. Methotrexate was included only as a pharmacological cytotoxic reference for comparative computational assessment and was not considered a validated target-specific ligand for *Bax*, *Bcl-2* or *p53*. These findings agree with previous reports describing anthraquinone interactions with apoptosis-related proteins. However, this hypothesis requires further validation.^62,64–69^

The resulting redocking RMSD values were 1.78 Å and 1.93 Å for *Bcl-2*, and *p53*, respectively. For Bax (PDB ID: 4BD6), potential binding cavities were identified automatically using Molegro Virtual Docker, and docking was performed within the selected cavity. However, conventional redocking validation could not be performed because the selected structure contains a *Bax*-derived BH3 peptide rather than a suitable co-crystallized small-molecule reference ligand. Consequently, a reference-pose RMSD could not be calculated for *Bax*. To further assess the predicted *Bax*–ligand complex, we examined the interacting amino acid residues and the chemical plausibility of the predicted noncovalent interactions. Although this analysis provides a qualitative assessment of the predicted binding mode, it does not substitute for redocking validation. Accordingly, the *Bax* docking results are interpreted cautiously as exploratory computational predictions rather than experimentally validated binding poses.

#### 3.6.6 Prediction of drug-likeness and oral bioavailability

Following biological evaluation, the pharmacokinetic properties and drug-likeness of the isolated compounds were predicted using SwissADME online tool (Table S9, Fig. S54 and S55). Most anthraquinones complied with established drug-likeness criteria and showed predicted physicochemical and pharmacokinetic properties. However, these predictions do not verify oral bioavailability or pharmacokinetic performance.

These findings are consistent with previous studies demonstrating that anthraquinones generally satisfy standard *in silico* drug-likeness criteria and possess predicted pharmacokinetic characteristics, providing preliminary computational prediction for their physicochemical and pharmacokinetic properties.^69–72^

#### 3.6.7 Prediction of mutagenicity, hepatotoxicity and cardiotoxicity

Toxicity of the isolated compounds and methotrexate was predicted *in silico* using pkCSM (Table S10). 5A/5B showed no predicted hepatotoxicity, cardiotoxicity, or mutagenicity according to the pkCSM predictions, whereas compounds 1–4 exhibited predicted mutagenicity. Overall, the isolated metabolites as well as methotrexate exhibited different predicted toxicological profiles using the pkCSM model.

Previous reports showed that quinone scaffold is associated with toxicological liabilities, particularly following prolonged exposure.^56,73,74^ Therefore, although the isolated compounds showed predicted *in silico* pharmacokinetic profiles, comprehensive *in vivo* pharmacokinetic, toxicity, and efficacy studies are required.

## 4 Conclusion

The marine-derived fungus *Aspergillus amstelodami* AUMC 16413 (Am-10), isolated from the Egyptian Red Sea macroalga *Sargassum aquifolium*, represents a source of structurally diverse secondary metabolites. Comprehensive metabolomic profiling tentatively annotated 53 metabolites, including anthraquinones, dihydroisocoumarins, prenylated benzaldehydes, indole diketopiperazine alkaloids, and lactones. Several anthraquinone derivatives were successfully isolated, purified, and structurally characterized as danthron, catenarin, questin, rubrocristin, questinol, endocrocin, and dermolutein. Anthraquinones were preferentially targeted for isolation based on their detection in the extract and their relatively high signal intensities in the LC-MS profile. These characteristics, together with their amenability to chromatographic separation and purification, supported their selection for further isolation and structural characterization.

The EtOAc extract exhibited cytotoxic activity against MCF-7 breast cancer cells, with greater activity toward MCF-7 cells than the corresponding normal cells under the conditions tested. Flow-cytometric analysis indicated an alteration in cell-cycle distribution and an increase in apoptotic cell populations, while RT-qPCR analysis showed changes in the expression of apoptosis-related genes, including increased *Bax* and *p53* and decreased *Bcl-2* expression. These findings are consistent with the involvement of apoptosis-related signalling, although they do not by themselves establish a specific apoptotic pathway or molecular mechanism. Among the isolated compounds, the 5A/5B mixture showed cytotoxic activity; however, because 5A and 5B were not individually separated, their respective contributions to this activity cannot be distinguished. Molecular docking provided computational predictions of possible interactions between the isolated metabolites and *Bax*, *Bcl-2*, and *p53*, but these results should be considered hypothesis-generating rather than experimental evidence of mechanism.

*In silico* ADME and toxicity analyses provided predicted ADME properties and toxicological profiles for the investigated metabolites, which require experimental validation. Overall, the findings highlight *A. amstelodami* AUMC 16413 as a source of bioactive secondary metabolites and provide a basis for further investigation. Future studies using isolated individual compounds, protein-level and functional mechanistic assays, and appropriate *in vivo* models are needed to clarify the compounds responsible for the observed activity and to further evaluate their biological properties.

## Author contributions

Conceptualization: E. T. B., A. E. R., K. A. S., A. Z., methodology, E. T. B., M. A. F., D. F., A. B.-K.; software: E. T. B., M. A. F. validation, A. E. R., D. F., A. B.-K., K. A. S., A. Z.; formal analysis, E. T. B., M. A. F., D. F., A. B.-K.; investigation. E. T. B., A. E. R., D. F., A. B.-K., A. Z.; resources, A. E. R., K. A. S., A. Z.; data curation, E. T. B., A. E. R., K. A. S., A. Z.; writing – original draft editing.; E. T. B., A. E. R., A. Z.; writing—review and editing. E. T. B., A. E. R., M. A. F., K. A. S., A. Z., supervision, A. E. R., K. A. S., A. Z. All authors have read and approved the final submitted version of the manuscript.

## Conflicts of interest

The authors declare no conflict of interest.

## Supplementary Material

RA-OLF-D6RA07402B-s001

## Data Availability

No data were used other than those described in the main manuscript and supplementary information (SI) file. Supplementary information is available. See DOI: https://doi.org/10.1039/d6ra07402b.
