# Supplementary material for: Metabolomics-guided discovery of cytotoxic anthraquinones from the Red Sea-derived fungus Aspergillus amstelodami associated with Sargassum aquifolium
Source: RSC Adv. 2026 Nov 3. Online ahead of print. doi: 10.1039/d6ra07402b (PMC13729387; doi:10.1039/d6ra07402b)
Supplement: Supplementary file 1 [file RA-OLF-D6RA07402B-s001.pdf]

Supplementary Information (SI) for RSC Advances.
This journal is © The Royal Society of Chemistry

Supplementary Data

Metabolomics-guided discovery of cytotoxic anthraquinones from the Red Sea-derived fungus *Aspergillus amstelodami* associated with *Sargassum aquifolium*

Ebtisam T. Badawy[1], Amany E. Ragab[1], Mohamed A. Farag[2], Doris Finkelmeier[3], Anke Burger-Kentischer[3], Kamilia A. Abo El-Seoud[1], Ahmed Zayed[1*]

[1] Department of Pharmacognosy, Tanta University, College of Pharmacy, El-Geish Street (Medical campus), 31527 Tanta, Egypt

[2] Department of Pharmacognosy, Faculty of Pharmacy, Cairo University, Kasr El-Aini Street, Cairo, 11562, Egypt

[3] Department Biofabrication and Material Development, Fraunhofer Institute for Interfacial Engineering and Biotechnology, Nobelstrasse 12, 70569, Stuttgart, Germany

*Correspondence

Dr. Ahmed Zayed

Department of Pharmacognosy, Tanta University, College of Pharmacy, El-Geish Street (Medical campus), 31527 Tanta, Egypt
e-mail: ahmed.zayed1@pharm.tanta.edu.eg

List of Figures

Fig. S1: *Sargassum aquifolium* macroalgae sample. 13

Fig. S2: (I) *Aspergillus flavus* var. *columnaris* AUMC16405: yellowish-green colony on Czapek`s agar (A) and rough-walled conidiophore with columnar conidial head and chains of conidia (B). (II) *Aspergillus sydowii* AUMC16417: Blue colony on Czapek`s agar (A) and conidiophore with small conidial head (B). (III) TLC of three isolates extracts obtained from culturing on rice, wheat and potato dextrose broth (PDB); A) *A. flavus*, B) *A.amstelodami*, C) *A. sydowii,* using CH_2Cl_2: EtOAc (7:3) as solvent system. P....14

Fig. S3: Column chromatography purification scheme of compounds 1-5 from Am-10 EtOAc extract. 15

Fig. S4: Represented chemical classes of *A. amstelodami* metabolites from UPLC-HRMS/MS....15

Fig. S5: Chemical structures of some annotated compounds from UPLC-HRMS/MS of Am-10 EtOAc extract. 16

Fig. S6: Proposed MS/MS fragmentation pattern of emodin (45). 17

Fig. S7: Proposed MS/MS fragmentation of dihydro-isocoumarin polyketides (eurotiumide). 19

Fig. S8: Proposed MS/MS fragmentation tree of flavoglaucin obtained from SIRIUS software. 20

Fig. S9: LC-MS chromatogram of compound 1....21

Fig. S10: LR-MS spectrum (-ve) of compound 1. 22

Fig. S11: UV spectrum of compound 1 (MeOH). 23

Fig. S12: ^{13}C NMR spectrum of compound 1 (DMSO-d_6, 100.6 MHz). 25

Fig. S 13: ^{1}H NMR spectrum of compound 1 (DMSO-d_6, 400 MHz). 26

Fig. S14: COSY spectrum of compound 1 (DMSO-d_6). 27

Fig. S15: HSQC spectrum of compound 1 (DMSO-d_6). 28

Fig. S16: HMBC spectrum of compound 1 (DMSO-d_6). 29

Fig. S17: LC-MS total ion chromatogram of compound 2. (I) Diode array spectrum (left panel) and UV spectrum (right panel). (II) In negative ion mode (left panel) and mass spectrum (right panel). (III) In positive ion mode (left panel) and MS spectrum (right panel). 30

Fig. S18: HRESI-MS (-ve) of compound 2....31

Fig. S19: ^{13}C NMR spectrum of compound 2 (DMSO-d_6). 32

Fig. S20: ^{1}H NMR spectrum of compound 2 (DMSO-d_6, 600 MHz) after water suppression....33

Fig. S21: COSY spectrum of compound 2 (DMSO-d_6)....35

Fig. S22: HSQC spectrum of compound 2 (DMSO-d_6). 36

Fig. S23: HMBC spectrum of compound 2 (DMSO-d_6)....37

Fig. S24: Analytical LCMS (photodiode array detector) analysis of compound 3A (in blue color) and 3B (in orange color). 38

Fig. S25: LC-MS total ion chromatogram of compound 3A. (I) Diode array spectrum (left panel) and UV spectrum (right panel). (II) In negative ion mode (left panel) and mass spectrum (right panel). (III) In positive ion mode (left panel) and MS spectrum (right panel). 39

Fig. S26: HRESI-MS (-ve) of compound 3A. 40

Fig. S27: ^{13}C NMR spectrum (DMSO-d_6, 151 MHz) of compound 3A (in blue color) and 3B (in orange color). 41

Fig. S28: ^{1}H NMR spectrum (DMSO-d_6, 600 MHz) of compound 3A (in blue color) and 3B (in orange color)....42

Fig. S29: COSY spectrum (DMSO-d_6) of compounds 3A (in blue color) and 3B (in orange color). 43

Fig. S30: HSQC spectrum (DMSO-d_6) of compounds 3A (in blue color) and 3B (in orange color). 44

Fig. S31: HMBC spectrum (DMSO-d_6) of compounds 3A (in blue color) and 3B (in orange color). 45

Fig. S32: LC-MS total ion chromatogram of compound 3B. (I) Diode array spectrum (left panel) and UV spectrum (right panel). (II) In negative ion mode (left panel) and mass spectrum (right panel). (III) In positive ion mode (left panel) and MS spectrum (right panel). 46
Fig. S33: HRESI-MS (-ve) of compound 3B. 47
Fig. S34: LC-MS total ion chromatogram of compound **4**. (I) Diode array spectrum (left panel) and UV spectrum (right panel). (II) In negative ion mode (left panel) and mass spectrum (right panel). (III) In positive ion mode (left panel) and MS spectrum (right panel). 48
Fig. S35: HRESI-MS (-ve) of compound 4. 49
Fig. S36: ^{1}H NMR spectrum of compound 4 (DMSO-d_6). 50
Fig. S37: ^{1}H NMR spectrum of compound 4 after water suppression. 51
Fig. S38: ^{13}C NMR spectrum of compound 4 (DMSO-d_6). 52
Fig. S39: COSY spectrum (DMSO-d_6, 600 MHz) of compounds 4. 53
Fig. S40: HSQC spectrum (DMSO-d_6) of compound 4. 54
Fig. S41: HMBC spectrum (DMSO-d_6) of compound 4. 55
Fig. S42: Analytical LCMS (photodiode array) analysis of compound 5B in orange and 5A in blue. 56
Fig. S43: LC-MS total ion chromatogram of compound 5B. (I) Diode array spectrum (left panel) and UV spectrum (right panel). (II) In negative ion mode (left panel) and mass spectrum (right panel). (III) In positive ion mode (left panel) and MS spectrum (right panel). 57
Fig. S44: HRESI-MS (-ve) of compound 5B. 58
Fig. S45: ^{13}C NMR spectrum (DMSO-*d6,* 151 MHz) of compounds 5B (in orange color) and 5A (in blue color). 59
Fig. S46: ^{1}H NMR spectrum (DMSO-*d6*) of compound 5B (in orange color) and 5A (in blue color). 60
Fig. S47: COSY spectrum (DMSO-*d6*) of compound 5B (in orange color) and 5A (in blue color). 61
Fig. S48: HSQC spectrum (DMSO-d_6) of compound 5B (in orange color) and 5A (in blue color). 62
Fig. S49: HMBC spectrum (DMSO-d_6) of compound 5B (in orange color) and 5A (in blue color). 63
Fig. S50: LC-MS total ion chromatogram of compound 5A. (I) Diode array spectrum (left panel) and UV spectrum (right panel). (II) In negative ion mode (left panel) and mass spectrum (right panel). (III) In positive ion mode (left panel) and MS spectrum (right panel). 64
Fig. S51: HRESI-MS (-ve) of compound 5A. 65
Fig. S52: (I) Effect of DMSO, methotrexate and SDS on breast MCF-7 and colon Caco-2 cancer cell lines and (II) Effect of Am-10 on normal breast MCF10a and normal colon FHC cell lines. (III): Methotrexate dose response curve. 66
Fig. S53: Superimposable docked poses of methotrexate and metabolites 1-5 (all presented as sticks) to apoptosis genes (A): *Bax* (PDB ID:4BD6), (B): *Bcl-2* (PDB ID: 6O0K) and (C) *p53* (PDB ID: 8DC6). Methotrexate (olive green), compound 1 (white), compound 2 (sky blue), compound 3A (red), compound 3B (green), compound 4 (Light green) compound 5B (Yellow) and compound 5A (blue). 67
Fig. S54: Bioavailability radar of methotrexate and compounds 1-5 obtained from SWISSADME. The radar plot is based on six important physicochemical properties that influence oral bioavailability, namely lipophilicity (LIPO), molecular size (SIZE), polarity (POLAR), solubility (INSOLU), flexibility (FLEX) and saturation (INSATU). The pink-colored region in the radar represents the optimal physicochemical space associated with good oral bioavailability. Compound 1 (A), 2(B), 3A(C), 3B(D), 4(E), 5B(F), 5A(G), and methotrexate (H). 68
Fig. S55: Predicted Brain Or IntestinaL EstimateD permeation (BOILED-Egg) plot. BBB (blood brain barrier), HIA (human intestinal absorption), PGP- (not P-glycoprotein substrate). 68

List of Tables:

Table S1: Evaluation of crude extract production by the isolated fungi using different fermentation media (rice, wheat, and potato dextrose broth)......68
Table S2: R_f values and TLC detection results of purified compounds isolated from *A. amstelodami* (Am-10). 69
Table S3: Gene primers for *Bax*, *Bcl-2* and *p53* apoptotic genes. 69
Table S4: Tentatively annotated metabolites detected in *A. amstelodami* (Am-10) EtOAc extract by UPLC-HRMS/MS, including retention time, molecular formula, diagnostic fragment ions, and relative abundance. 70
Table S5: ^{1}H, ^{13}C NMR (DMSO-d_6, 600 MHz and 151 MHz) except 1 (400 MHz and 100.6 MHz) of compound **1-3** 73
Table S6: ^{1}H, ^{13}C NMR (DMSO-d_6, 600 MHz and 151 MHz) of **compound 4** and **5**......75
Table S7: Effect of Am-10 on cell cycle arrest and apoptosis of breast cancer cell line MCF-7......76
Table S8: Docking results of compounds 1-5 (kJ/mol) to apoptosis- related proteins *Bax* (PDB ID:4BD6), *Bcl-2* (PDB ID: 6O0K) and *p53* (PDB ID: 8DC6)......77
Table S9: Physicochemical properties methotrexate and compounds 1-5 for prediction of drug-likeness and oral bioavailability......78
Table S10: *In silico* toxicity prediction of purified anthraquinones using pkCSM......78

Structure elucidation of the isolated compounds

Seven compounds were chromatographically separated and identified from the EtOAc extract of Am-10 via ^{1}HNMR, ^{13}C NMR, 2D NMR (e.g., COSY, HSQC and HMBC), UV, LR-MS and HRESI-MS as well as physical properties (**Table S2**) as follows:

Compound 1 was isolated as yellow amorphous powder (0.01 g). It showed R_f values and TLC characterization as presented in **Table S2**. Analysis by low resolution-MS analysis (LR-MS, negative mode) showed a deprotonated molecular ion peak at *m/z* 239.3, corresponding to the molecular formula $C_{14}H_8O_4$ (**Figure S9** and **S10**). The UV absorption (MeOH) λ$_{max}$ at 223, 252, 282.8, and 428.1 nm (**Figure S11**) suggested an extended conjugated aromatic system, consistent with anthraquinone chromophore [1]. The ^{13}C NMR (DMSO-d_6, 100.6 MHz; **Table S5** and **Figure S12**) spectrum revealed 8 signals corresponding to 14 carbons, including two conjugated carbonyl carbons at δ$_C$ 192.5 and 181.8, supporting the presence of an anthraquinone core. The presence of only eight carbon resonances for fourteen carbons indicated a highly symmetrical structure with several pairs of magnetically equivalent carbon. Signals at δ$_C$ 124.9, 137.9, and 119.8 represented two magnetically equivalent carbons for each, which further supported the molecular symmetry. The signal at δ$_C$ 161.8 suggested two magnetically equivalent oxygenated aromatic carbons assigned for (C-1 and C-8). The remaining aromatic carbons appeared at δ$_C$ 116.4 (C-12 and C-13) and 133.6 (C-11 and C-14), consistent with a substituted polycyclic aromatic system. The ^{1}H NMR (DMSO-d_6, 400 MHz; **Figure S13**) spectrum showed six aromatic protons forming two symmetrical groups. Signals at δ$_H$ 7.29 (2H, d, J = 8.0 Hz) corresponded to H-2 and H-7. Signal at δ$_H$ 7.74 (2H, t) was assigned to H-3 and H-6. In contrast, protons at δ$_H$ 7.60 (2H, d, J = 4 Hz) were proposed for H-4 and H-5. A downfield singlet at δ$_H$ 11.82 indicated intramolecular hydrogen bonding of hydroxyl groups. These chemical shifts indicated a typical anthraquinone proton pattern with two identical aromatic rings. The coupling constants and splitting patterns suggested ortho and meta correlations between the aromatic protons, which were further confirmed by ^{1}H–^{1}H COSY (**Figure 6** and **S14**). Correlations between protons and corresponding carbons were confirmed by HSQC (**Figure S15**). HMBC correlations (**Figures 6** and **S16**) further established the substitution pattern. The proton H-2 showed long-range correlations to C-1, C-4, and C-13. H-3 correlated with C-1 and C-14. The

aromatic protons H-4 and H-5 exhibited correlations to the carbonyl carbon C-10, confirming the position of the quinone carbonyl groups. Additionally, correlations from H-6 and H-7 to C-8 and from H-2, H-3 to C-1 supported the symmetrical dihydroxy substitution at C-1 and C-8.

Based on the combined spectroscopic data (^1H NMR, ^{13}C NMR, COSY, HSQC and HMBC), as well as the molecular weight, compound-**1** was identified as danthron (1,8-dihydroxyanthraquinone) in agreement with reported literature for danthron isolated from the marine fungus *Chromolaenicola* sp. [2]. Danthron is known for its antiangiogenic, antioxidant, and antiproliferative activity on murine B16-F10 melanoma cells [2,3]. This is the first report for isolation of danthron from *A. amstelodami*.

Compound 2 was isolated as a red amorphous powder (0.008 g). It showed R_f values and TLC characterization as presented in **Table S2**. It showed UV λ$_{max}$ in MeOH at 226.1 and 487.1 (**Figure S17**) characteristic of anthraquinones. [1] The molecular formula of **2** was determined as $C_{15}H_{10}O_6$ based on the analysis of negative-ion mode HRESI-MS (**Figure S18**). It exhibited [M–H]⁻ ion peak at *m/z* 285.0405 (calculated *m/z* 285.0399, difference +2.11 ppm), corresponding to 11 degrees of unsaturation.

^{13}C NMR spectrum of **2** (DMSO-d_6, 151 MHz; **Table S5** and **Figure S19**) showed 15 signals including two conjugated carbonyl carbons at δ$_C$ 187.0 and 186.2, characteristic of the anthraquinones core. It also showed three signals for aromatic methine carbons, one methyl carbon at δ$_C$ 15.8, four oxygenated aromatic quaternary carbons, together with methyl substitution.

The ^1H NMR data of compound **2** (DMSO-d_6, 600 MHz) showed seven different peaks corresponding to nine protons (**Table S5** and **Figure S20**). Three singlets in the aromatic region, one methyl peak at δ$_H$ 2.26 and three intramolecularly hydrogen-bonded hydroxyl protons appeared as downfield broad singlets.

COSY spectrum supported the assignment of protons (**Figure S21**). The HSQC spectrum (**Figure S22**) confirmed the assignment of protons. Analysis of HMBC spectrum (**Figures 6** and **S23**) revealed the correlation from methyl group to C-3, C-2 and C-1, confirming its position at C-2. The proton at C-3 was correlated to C-14 and C-1.

These data established the structure of **2** as a substituted anthraquinone. Compound-**2** was elucidated as 1,4,5,7-tetrahydroxy-2-methylanthracene-9,10-dione (catenarin). It is a naturally occurring anthraquinone pigment previously isolated from various *Aspergillus* species with antioxidant, antimicrobial and anti-inflammatory [4,5]. This is the first report of isolation of catenarin from *A. amstelodami*.

3A/3B mixture was obtained as orange-red amorphous powder. It was analyzed using LC-MS analysis and PDA detection (**Figure S24**). It revealed that the fraction contained two closely related compounds with retention times of 6.76 and 7.18 min, described as **3A** and **3B**, respectively. Their individual structures were assigned based on HRESI-MS and NMR analyses.

Compound 3A eluted at RT 6.76 min (**Figure S24**). It exhibited UV λmax in MeOH at 223.1, 283.1 and 436.1 nm (**Figure S25**). The molecular formula was deduced as C16H12O5 based on HRESI-MS spectrum (negative ion mode) which revealed a deprotonated molecular ion peak at *m/z* 283.0594 [M–H]⁻ (calcd. for $C_{16}H_{12}O_{5}$, 283.0602, difference +2.83 ppm) as presented in **Figure S26**.

The ^{13}C NMR spectrum (DMSO-d6, 151 MHz) of compound **3A** showed 16 signals corresponding to 16 distinct carbons, including two downfield carbonyl carbon signals at δ$_C$ 186.3 and 182.4, suggesting the presence of a quinone moiety (**Table S5**, **Figure S27**). Moreover, three oxygenated quaternary aromatic carbons, three quaternary aromatic carbons and four methine aromatic carbons were observed. The presence of methyl carbon at δ$_C$ 21.4 and a methoxy carbon at δ$_C$ 56.3 further supported the substituted anthraquinone nucleus.

The ^{1}H NMR spectrum (DMSO-d$_6$, 600 MHz) showed four peaks corresponding to 4 aromatic protons, three doublets and one singlet, consistent with a highly substituted anthraquinone nucleus. Moreover, a singlet at δ$_H$ 2.40 (3H, s) was assigned to a methyl group, while a singlet at δ$_H$ 3.91 (3H, s) was ascribed to a methoxy group. A highly downfield singlet at δ$_H$ 13.10 (1H, s) was assigned to an intramolecularly hydrogen-bonded hydroxyl group (**Table S5**, **Figure S28**).

COSY, HSQC, and HMBC experiments (**Figures S29–S31**) established the substitution pattern. HMBC revealed correlations between the methyl protons and C-2, C-3, and C-4, confirming its position at C-3. The methoxy protons showed HMBC correlation with C-8,

confirming their attachment at this position. Additionally, H-4 showed correlations to the carbonyl carbon C-10. The combined spectroscopic data identified compound **3A** as questin (1,6-dihydroxy-8-methoxy-3-methylanthracene-9,10-dione). The data were in full agreement with previously reported literature data [6,7].

Questin is a fungal anthraquinone pigment previously isolated from *A. rubrum*, *A. cristatus*, *A. chevalieri* and *A. flavipes* with antibacterial activity [5,6,8,9]. This is the first report of isolation of questin from *A. amstelodami*.

Compound **3B** eluted at RT 7.18 min (**Figures S24**), showing UV spectrum $\square_{max}$ at 226.1, 276.1, and 487.1 nm, characteristic of a highly conjugated anthraquinone chromophore (**Figure S32**). It exhibited a molecular formula of $C_{16}H_{12}O_6$, as established by HRESI-MS in the negative-ion mode (**Figure S33**). It showed a deprotonated molecular ion peak at *m/z* 299.0555 [M–H]⁻ (calcd. 299.0549, difference -2.01 ppm).

The ^{13}C NMR spectrum (DMSO-d_6, 151 MHz; **Table S5, Figure S27**) exhibited 16 carbon signals, including two quinone carbonyl carbons, four oxygenated aromatic quaternary carbons, three aromatic methines, and signals corresponding to one methyl and one methoxy group, consistent with a substituted anthraquinone skeleton.

The ^{1}H NMR spectrum (DMSO-d_6, 600 MHz; **Table S5**, **Figure S28**) displayed three aromatic protons of which two meta-coupled protons appeared at $\square_H$ 6.90 (d, *J* = 1.8 Hz, H-6) and 7.32 (d, *J* = 1.8 Hz, H-8). Singlets at $\square_H$ 2.28 and 3.92 were assigned to methyl and methoxy groups, respectively. The COSY, HSQC, and HMBC spectra (**Figures S29-S31**) established the substitution pattern of the molecule. HMBC correlations of the methyl protons with C-1, C-2, and C-3 confirmed methyl substitution at C-2.

Comparison of the spectroscopic data of compound **3B** with those of questin **3A** [6,7] revealed the presence of an additional hydroxyl group, as indicated by the 16 Da increase in molecular mass. The disappearance of one aromatic proton signal and the shifts of aromatic carbons C-1, C-2, C-3, C-4, C-13 and C-14 further supported this assignment. The NMR differences further support the conclusion that compound **3B** is a hydroxylated form of questin. The combined HRESI-MS and

NMR data together with comparison to questin, compound **3B** was identified as rubrocristin (4-hydroxyquestin).

Rubrocristin is a fungal anthraquinone pigment previously reported from *A. cristatus* and *A. variecolor*, and has been shown to possess immunomodulatory activity. [5,10,11] This is the first report of isolation of rubrocristin from Am-10.

Compound 4 was purified as yellow-orange amorphous powder. The UV absorption λmax in MeOH at 222.1, 284.1 and 435.1 nm supported an extended conjugated aromatic chromophore, characteristic of anthraquinone derivatives (Figure S34).

Moreover, HRESI-MS (**Figure S35**) showed a deprotonated molecular ion peak at *m/z* 299.0555 [M-H]- (calculated *m/z* 299.0556, difference +0.33 ppm), which corresponds to the molecular formula $C_{16}H_{12}O_6$. The ^{1}H NMR (DMSO-d_6, 600 MHz) spectrum (**Table S6**, **Figures S36** and **S37**) exhibited four aromatic proton peaks, one methoxy at δ$_H$ 3.90 (3H, s) and one hydroxymethyl group at δ$_H$ 4.58 (2H, s).

The ^{13}C NMR (DMSO-d_6, 151 MHz) spectrum (**Table S6**, **Figure S38**) displayed 16 carbons including two carbonyl carbons, three oxygenated aromatic carbons, and four methine aromatic carbons. Moreover, two oxygenated aliphatic carbons at δ$_C$ 56.3 and 62.0 for the methoxy and hydroxymethyl group were observed, respectively.

COSY spectrum (**Figure S39**) supported the proximity of aromatic protons. The hydroxymethyl protons exhibited correlation to H-2 and H-4. The HSQC spectrum (**Figure S40**) further confirmed the correlation between protons and corresponding carbons. HMBC correlations (**Figure S41**) further established the structure. The methoxy protons correlated to C-8. The hydroxymethyl protons (CH_2OH) correlated with C-4, C-2, and C-3, thereby establishing the positions of the methoxy and hydroxymethyl groups at C-8 and C-3, respectively.

The combined data are in good agreement with those reported for questinol (1,6-dihydroxy-3-(hydroxymethyl)-8-methoxyanthracene-9,10-dione) [12].

Questinol is a well-known hydroxy anthraquinone metabolite produced by several marine-derived fungi species such as *A. variecolor* and *A. amstelodami.* It was reported to possess anti-inflammatory and anti-obesity activity [10,12].

LC-MS/PDA of **5A/5B mixture** revealed that it consisted of a mixture of two compounds (**Figure S42**). They were eluted at retention times of 4.85 and 5.43 min, corresponding to compounds **5A** and **5B**, respectively. Furthermore, various spectroscopic analyses were conducted to determine the chemical structures of the two compounds. Compound **5B** was discussed first.

Compound 5B eluted at 5.43 min by analytical LC-MS. It showed UV absorption λ_{max} at 222.1, 286.1, and 442.1 nm characteristic of anthraquinone (**Figure S43**). The HRESI-MS spectrum (negative ion mode) displayed a deprotonated molecular ion peak at *m/z* 313.0328 $[M–H]^-$ (calculated 313.0338, difference -3.19 ppm) , corresponding to the molecular formula $C_{16}H_{10}O_7$, which is consistent with an oxygenated anthraquinone (**Figure S44**).

The ^{13}C NMR (**Table S6** and **Figure S45**) spectrum (DMSO-d_6, 151 MHz) revealed 16 signals including two conjugated carbonyls, three oxygenated aromatic carbons, three quaternary aromatic carbons and three methine aromatic carbons. A methyl carbon appeared at δ_C 22.0. Moreover, a carboxylic carbon signal at δ_C 167.5 confirmed the presence of a-COOH group, a diagnostic feature of endocrocin-type anthraquinones [5].

The ^1H NMR (DMSO-d_6, 600 MHz) spectrum (**Table S6** and **Figure S46**) showed three aromatic protons, consistent with a highly substituted anthraquinone nucleus. A methyl singlet at δ_H 2.56 confirmed the presence of a methyl substituent on the aromatic ring. Two strongly deshielded singlets at δ_H 13.09 (1-OH) and 13.33 (8-OH) corresponded to intramolecularly hydrogen-bonded hydroxy protons. One carboxylic acid proton resonated at δ_H 11.14, a standard signal for endocrocin-type anthraquinones [5].

The COSY, HSQC and HMBC spectrum (**Figures S47-S49**) further established the assignment of the protons to their corresponding carbons. HMBC correlations (**Figures 6** and **S49**) further verified the substitution pattern. H-5 showed long-range correlations to C-7, C-12, and the quinone carbonyl C-10. H-7 exhibited correlations with C-5, C-12, and the oxygenated carbon C-6, supporting the position of hydroxyl groups at C-6 and C-8. The methyl protons showed HMBC correlation to C-4, confirming methyl substitution at C-3. The carboxyl group at C-2 was supported by the downfield carbon resonance at δ_C 167.5.

Based on the combined spectroscopic data, compound **5B** was identified as endocrocin, a known oxygenated anthraquinone commonly reported from *Aspergillus* (*Eurotium* species). The obtained NMR data were in good agreement with previously reported literature values for endocrocin isolated from *A. cristatus* [5]. This finding further confirms the ability of *A. amstelodami* to biosynthesize structurally diverse anthraquinones. This is the first report of endocrocin from *A. amstelodami*.

Compound 5A eluted at RT of 4.85 min by LC-MS and showed UV absorption maxima at 223.1, 285.1, and 442.1(**Figure S50**).

The HRESI-MS spectrum showed [M–H]⁻ at *m/z* 327.0464, (calculated 327.0454, difference -2.14 ppm) corresponding to the molecular formula $C_{17}H_{12}O_7$ (**Figure S51**). Comparison with that of the de-O-methylated analogue, endocrocin, clearly showed O-methylation at C-8 in compound **5A** results in an increase in molecular weight by 14 Da. Moreover, this was supported by the appearance of a methoxy signal in both ^{1}H and ^{13}C NMR spectra (**Table S6** and **Figures S45** and **S46**), and slight chemical shift changes in the aromatic region, particularly for C-11, C-12, C-7 and C-8.

Based on comprehensive spectroscopic analysis HRESI-MS, ^{1}H NMR, ^{13}C NMR, COSY, HSQC, and HMBC (**Figure**s **S45-S49**), compound **5A** was identified as dermolutein (O-methyl endocrocin). The obtained data are in good agreement with the reported literature for dermolutein [5]. It is a naturally occurring anthraquinone found in fungi, especially *Cortinarius* species [11] and recently isolated from *A. cristatus* [5]. It contributes to the yellow coloration of fungal fruiting bodies. Xie et al. reported that dermolutein and endocrocin exhibited moderate antioxidant and anti-inflammatory activities [5]. This result further highlights the structural diversity of anthraquinones produced by *A. amstelodami*, particularly in their hydroxylation and *O*-methylation patterns. To the best of our knowledge, this is the first report of the isolation of dermolutein from *A. amstelodami*. The relative chromatographic purity of the isolated compounds was estimated from photodiode array (PDA) chromatograms using the peak-area normalization method. The relative area percentage was calculated by dividing the integrated area of the peak assigned to the target compound by the sum of the integrated areas of all detected chromatographic peaks at the same detection wavelength and

multiplying by 100. The estimated relative chromatographic purities were 88.00%, 80.89%, and 85.70% for compounds 1, 2, and 4, respectively. For the mixtures containing compounds 3A/3B and 5A/5B, the corresponding relative peak-area percentages were 58.15% and 41.85%, and 13.38% and 86.62%, respectively. These values represent relative chromatographic area percentages under the applied analytical conditions rather than absolute chemical purity, because differences in UV absorptivity among components and the possible presence of co-eluting or undetected impurities may affect the estimates. Accordingly, the cytotoxicity results should be interpreted in the context of the estimated relative chromatographic compositions, and a contribution from minor impurities cannot be completely excluded.

Spectral properties of purified compounds

Danthron (**1**). Yellow amorphous powder, soluble in petroleum ether, CH_2Cl_2, MeOH and DMSO. UV (MeOH) λ$_{max}$ (nm): 223, 252, 282.8, and 428.1 nm. LR-MS (*m/z*) 239.3 Da $[M-H]^-$.^{1}H NMR (400 MHz, DMSO-d_6) and ^{13}C NMR (100.6 MHz, DMSO-d_6) as shown in **Table S5**.

Catenarin (2). Red amorphous powder, soluble in petroleum ether, chloroform, CH_2Cl_2, MeOH and DMSO. UV (MeOH) λ$_{max}$ (nm): 226.1 and 487.1. HRESI-MS *m/z* 285.0405 Da [M-H]. ^{1}H NMR (600 MHz, DMSO-d_6) and ^{13}C NMR (151 MHz, DMSO-d_6) as shown in **Table S5**.

Questin (3A): Orange amorphous powder. Slightly soluble in CH_2Cl_2, very soluble in MeOH and DMSO. UV (MeOH) λ$_{max}$ (nm): 223.1, 283.1, 436.1. HRESI-MS *m/z* 283.0594 [M–H]⁻.^{1}H NMR (600 MHz, DMSO-d_6) and ^{13}C NMR (151 MHz, DMSO-d_6) as shown in **Table S5**.

Rubrocristin (3B): Red amorphous powder. Slightly soluble in CH_2Cl_2, very soluble in MeOH and DMSO. UV (MeOH) λ$_{max}$ (nm): 226.1, 276.1, 487.1 HRESI-MS *m/z* 299.0550 [M–H]⁻. ^{1}H NMR (600 MHz, DMSO-d_6) and ^{13}C NMR (151 MHz, DMSO-d_6) as shown in **Table S5**.

Questinol (4): Yellow-orange amorphous powder. Slightly soluble in CH_2Cl_2, and soluble in MeOH, and DMSO. UV (MeOH) λ$_{max}$ (nm): 222.1, 284.1, 435.1. HRESI-MS *m/z* 299.0555 [M–H]⁻. ^{1}H NMR (600 MHz, DMSO-d_6) and ^{13}C NMR (151 MHz, DMSO-d_6) as shown in **Table S6**.

Endocrocin (5B): Brownish yellow amorphous powder, soluble in MeOH, and DMSO. UV (MeOH) λ$_{max}$ (nm): 222.1, 286.1, 442.1, HRESI-MS *m/z* 313.0328 [M–H]⁻ (calcd for $C_{16}H_9O_7$, 313.0297).^{1}H NMR (600 MHz, DMSO-d_6) and ^{13}C NMR (151 MHz, DMSO-d_6) as shown in **Table S6**.

Dermolutein (5A): Brownish-yellow amorphous powder, soluble in MeOH, and DMSO. UV (MeOH) λ$_{max}$ (nm): 226.1, 276.1, 487.1. HRESI-MS *m/z* 327.0464 [M – H]⁻. ^{1}H NMR (600 MHz, DMSO-d_6) and ^{13}C NMR (151 MHz, DMSO-d_6) as shown in **Table S6**.

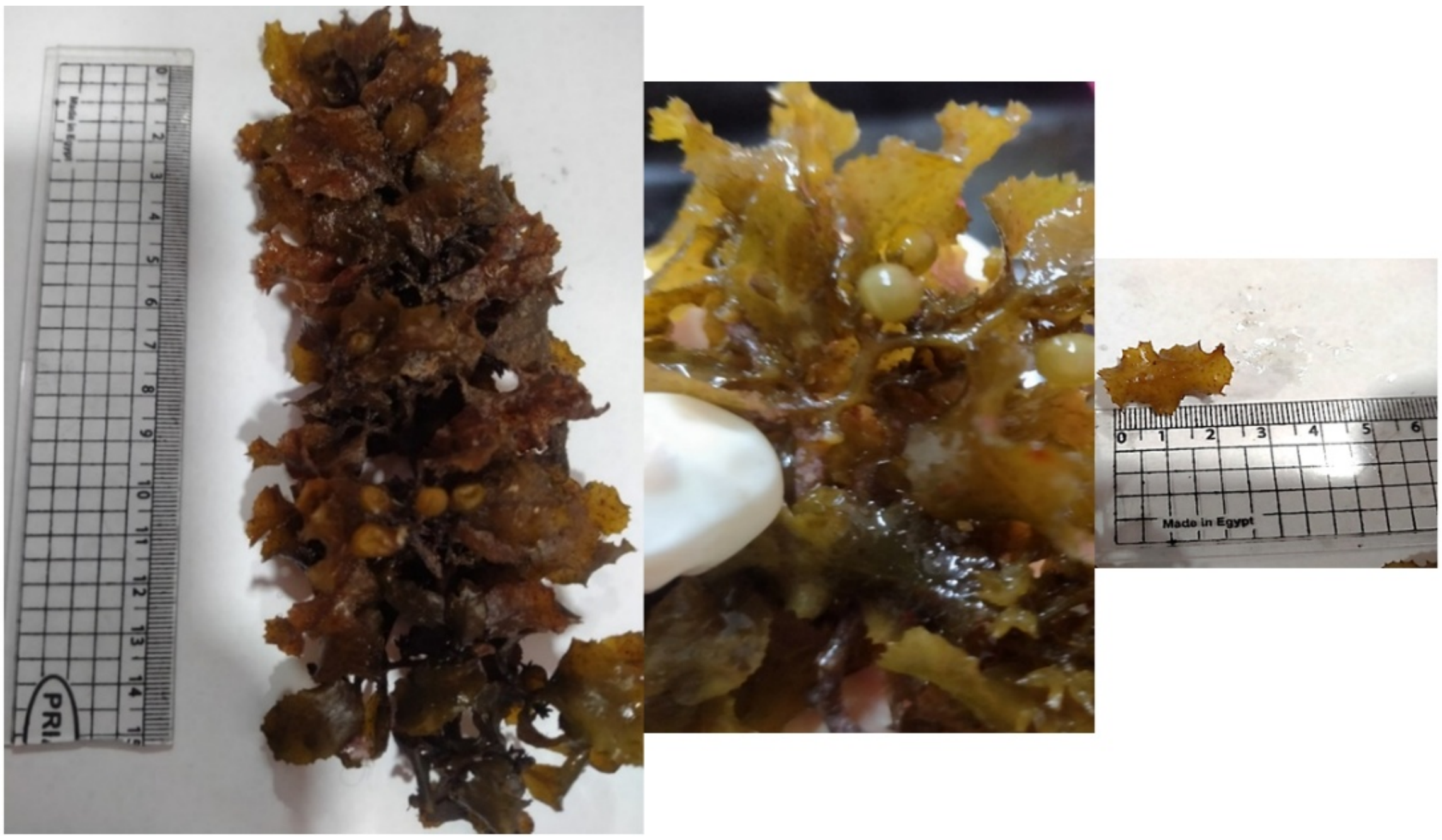

Fig. S1: *Sargassum aquifolium* macroalgae sample.

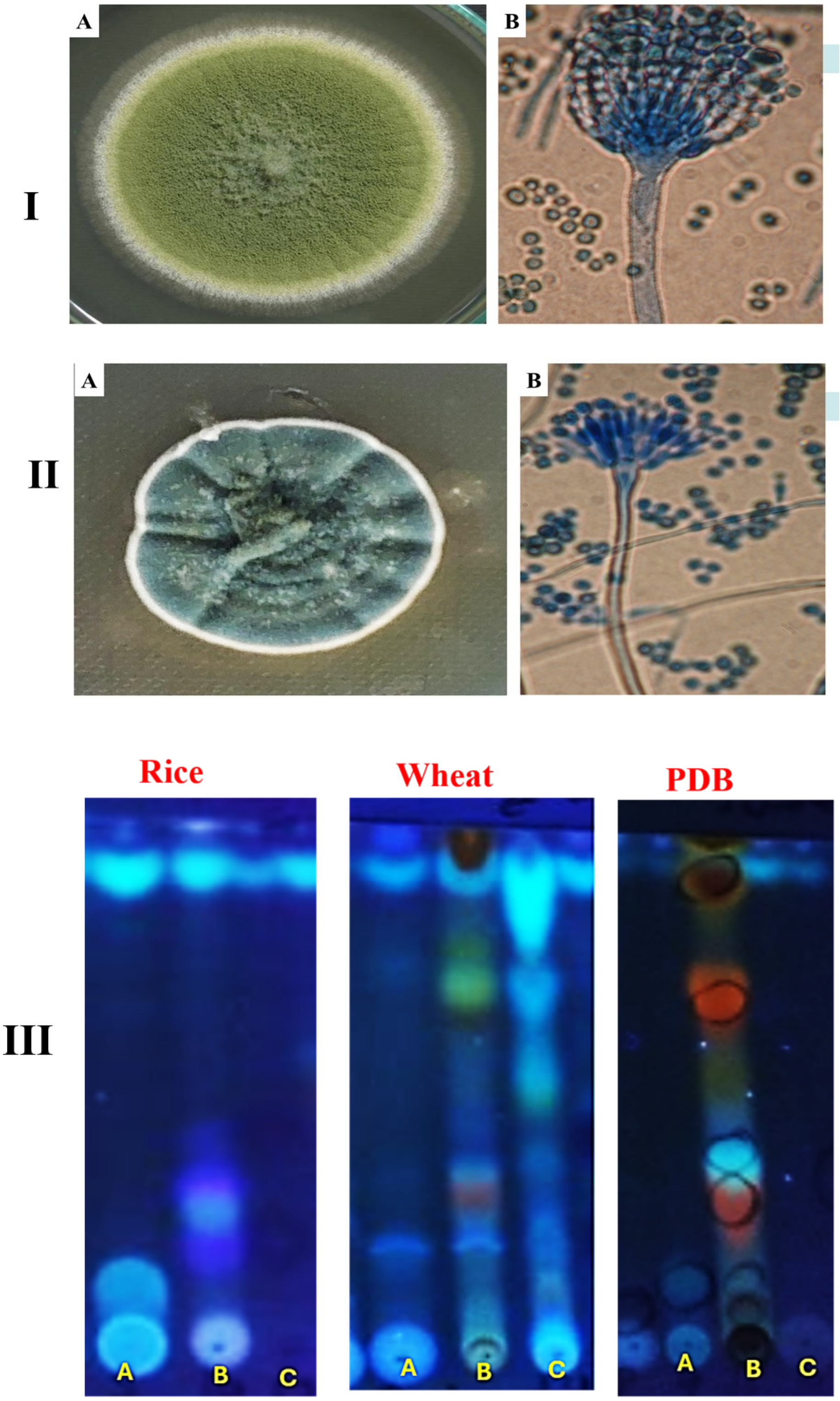

Fig. S2: (I) *Aspergillus flavus* var. *columnaris* AUMC 16405: yellowish-green colony on Czapek`s agar (A) and rough-walled conidiophore with columnar conidial head and chains of conidia (B). **(II)** *Aspergillus sydowii* AUMC 16417: Blue colony on Czapek`s agar (A) and conidiophore with small conidial head (B). **(III)** TLC of three isolates extracts obtained from culturing on rice, wheat and potato dextrose broth (PDB); A) *A. flavus*, B) *A.amstelodami*, C) *A. sydowii,* using CH_2Cl_2: EtOAc (7:3) as solvent system.

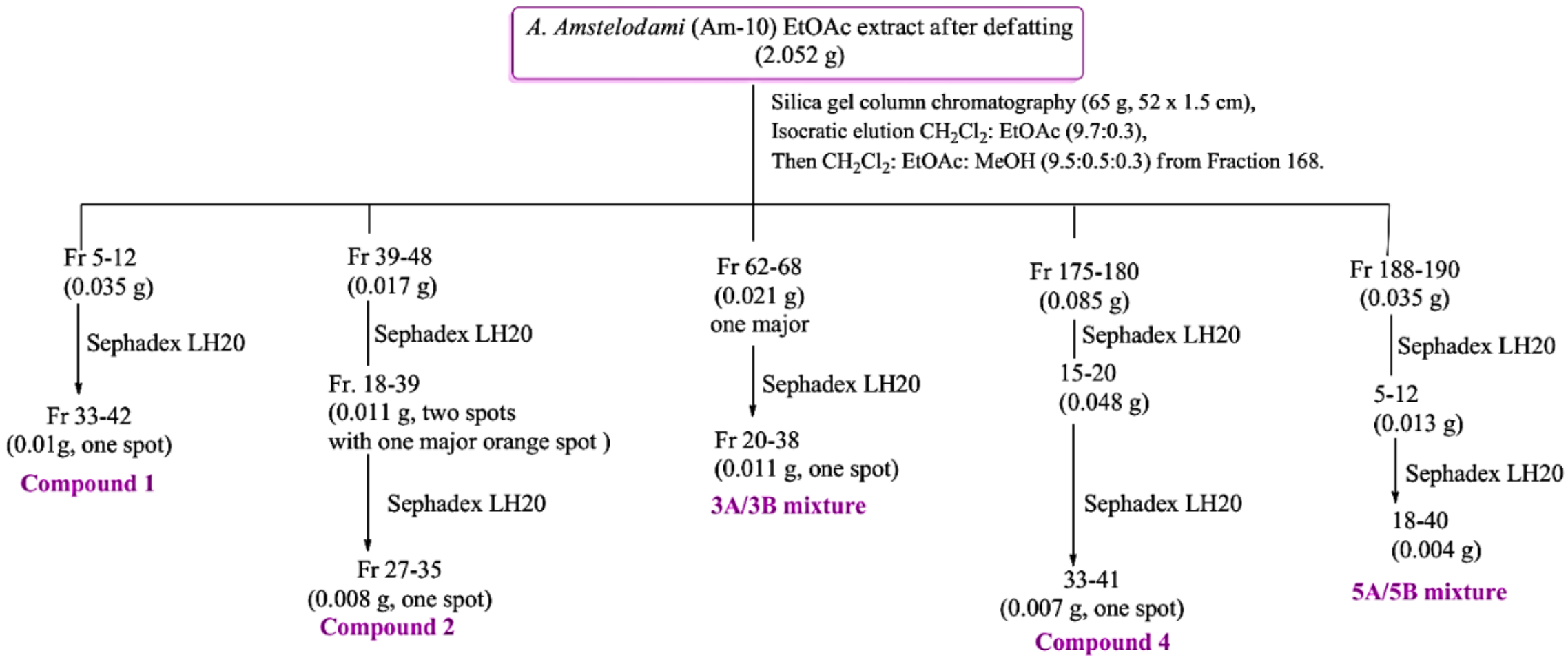

Fig. S3: Column chromatography purification scheme of compounds **1-5** from Am-10 EtOAc extract

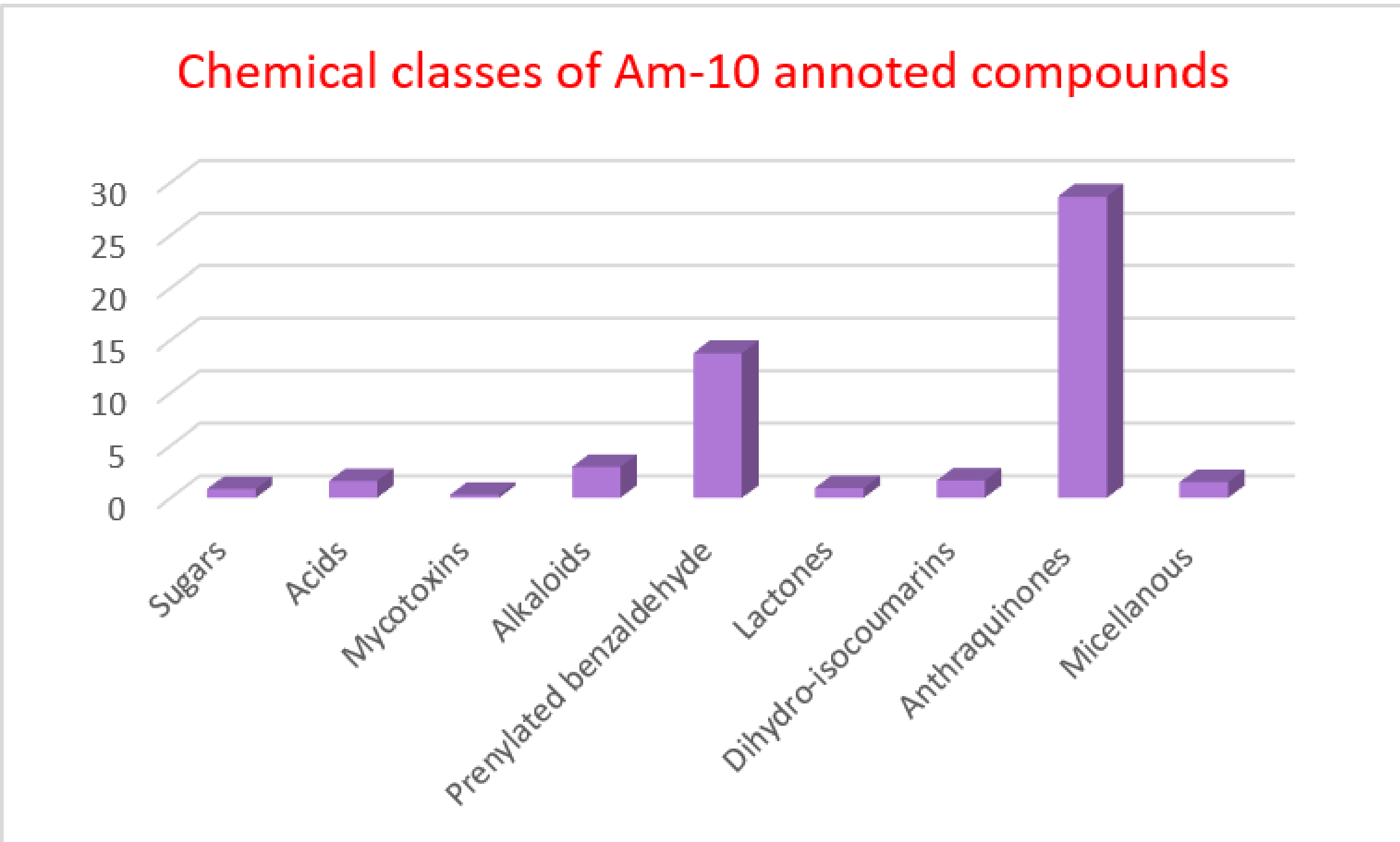

Fig. S4: Represented chemical classes of *A. amstelodami* metabolites from UPLC-HRMS/MS

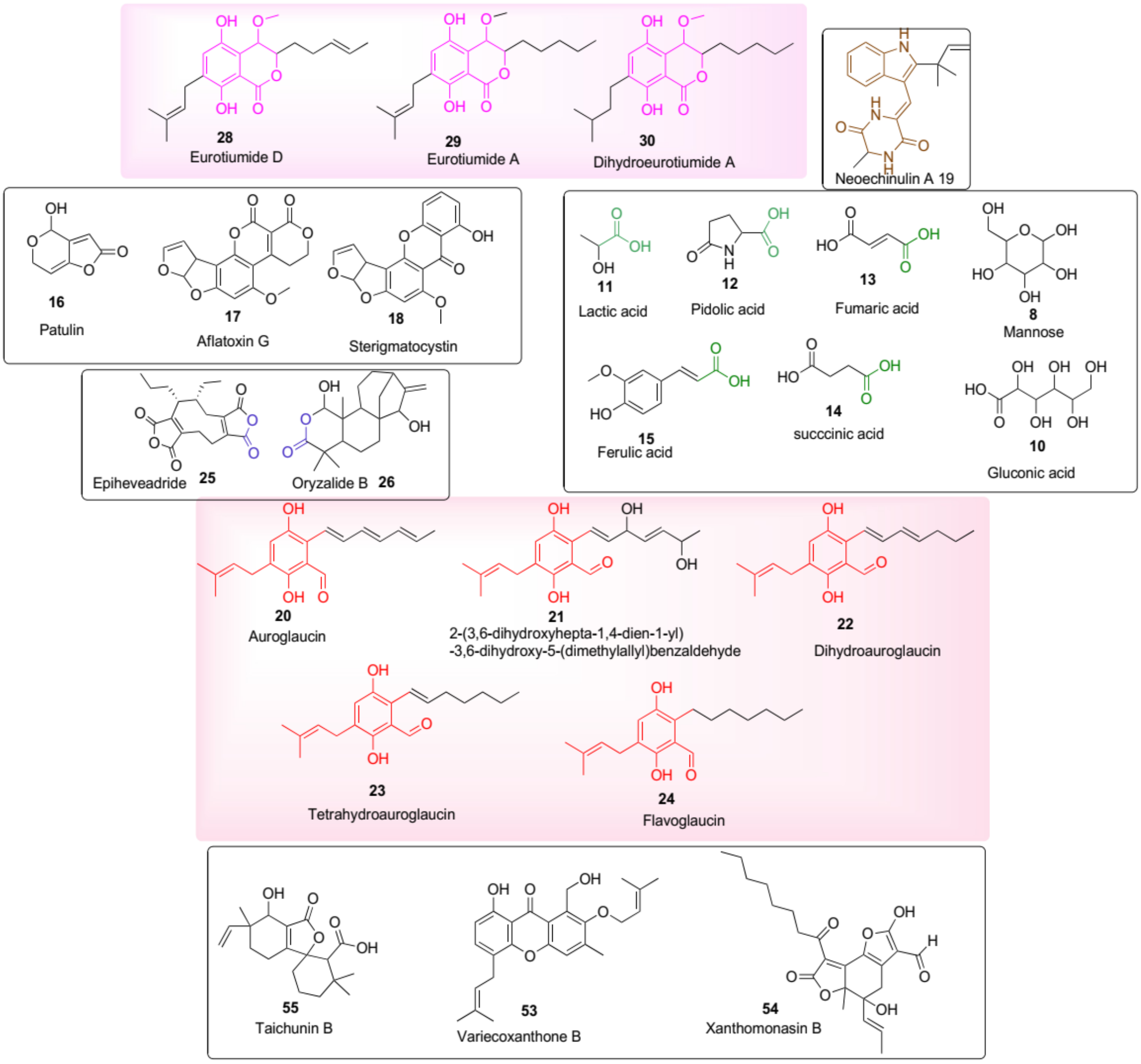

Fig. S5: Chemical structures of some annotated compounds from UPLC-HRMS/MS of Am-10 EtOAc extract.

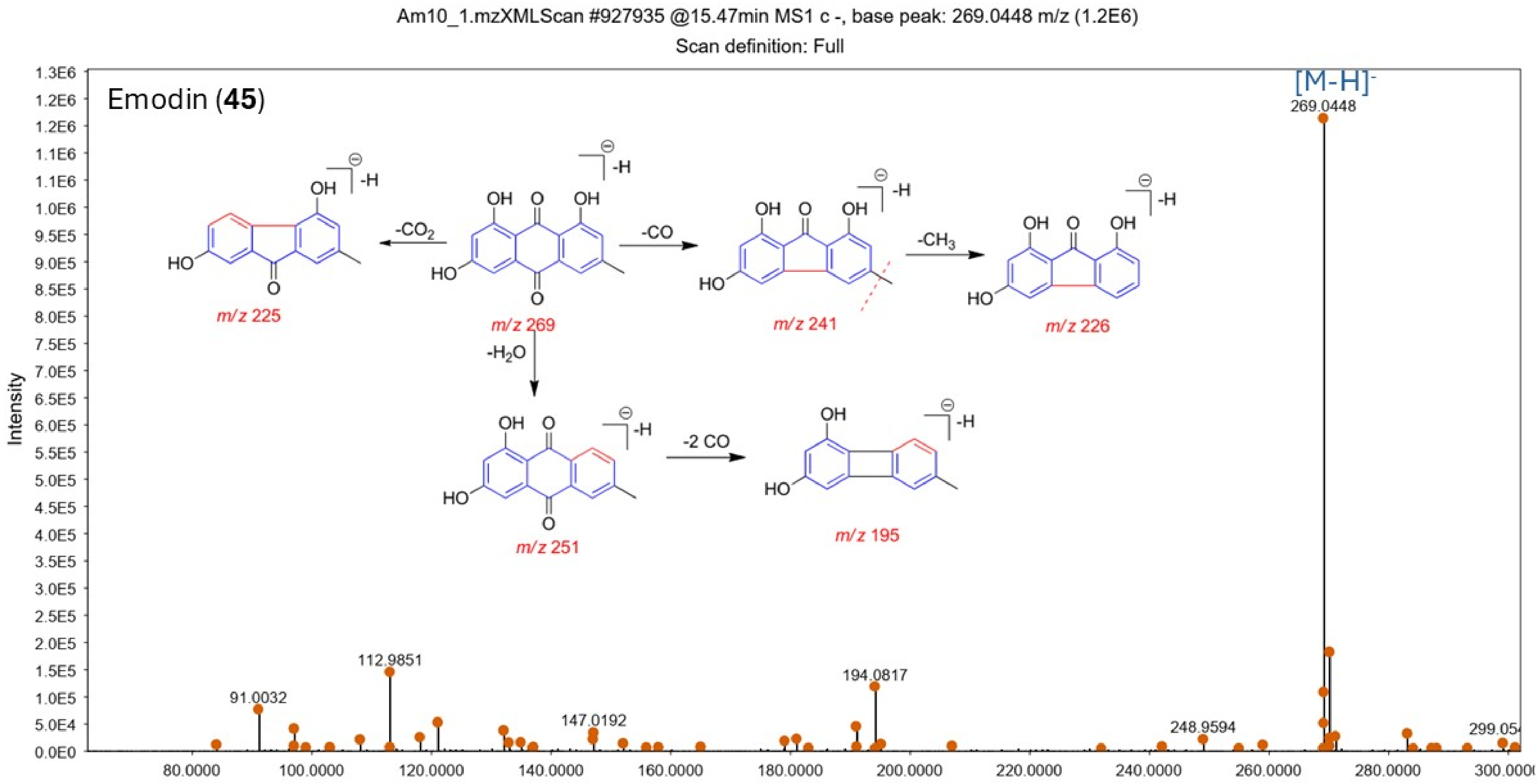

Fig. S6: Proposed MS/MS fragmentation pattern of emodin (45).

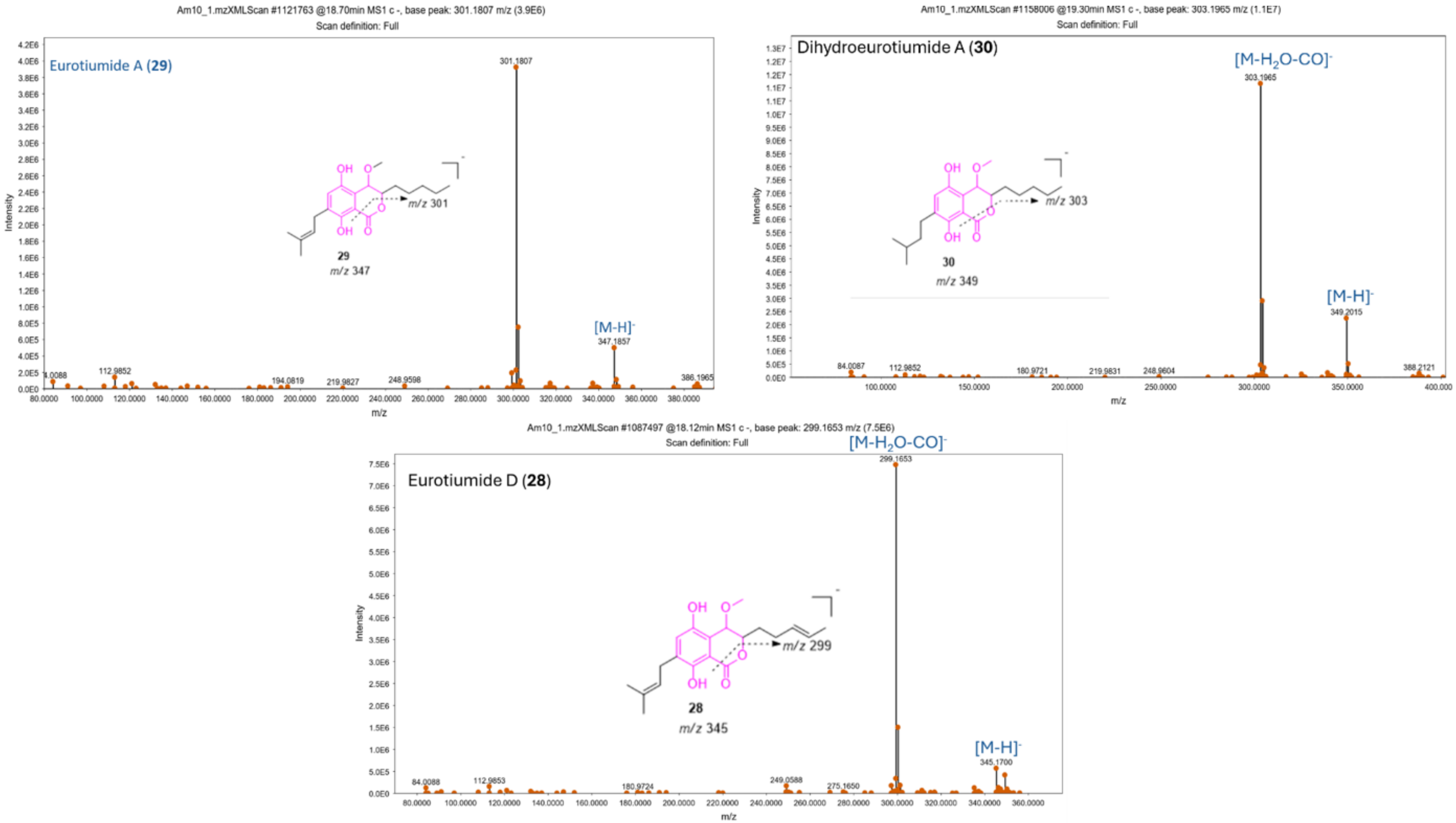

Fig. S7: Proposed MS/MS fragmentation of dihydro-isocoumarin polyketides (eurotiumide).

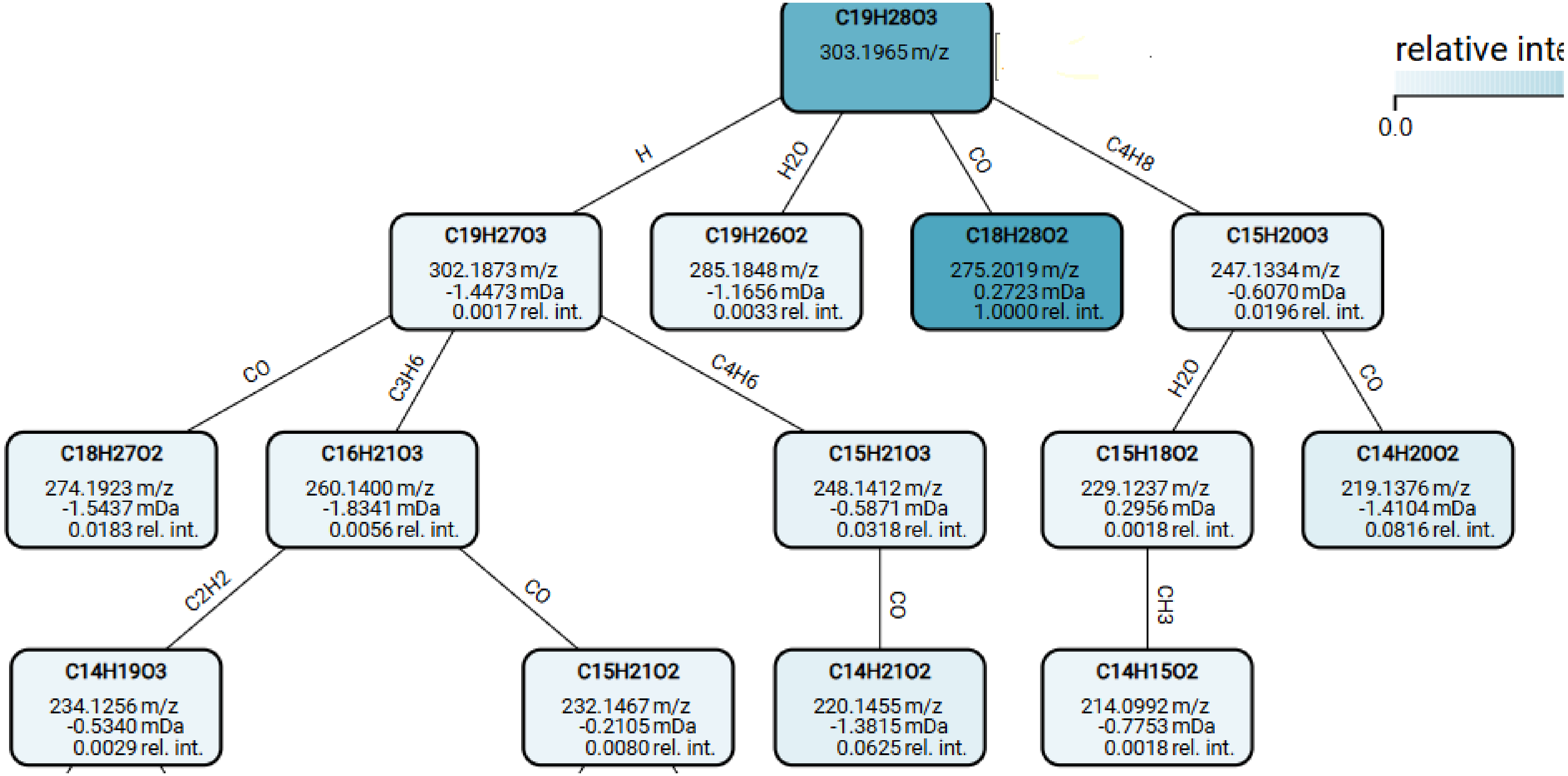

Fig. S8: Proposed MS/MS fragmentation tree of flavoglaucin obtained from SIRIUS software.

Compound 1:

Sample Report:

Sample 1281 Date 29-Dec-2025 Time 11:24:45 Description TA

1: MS ES- :BPI

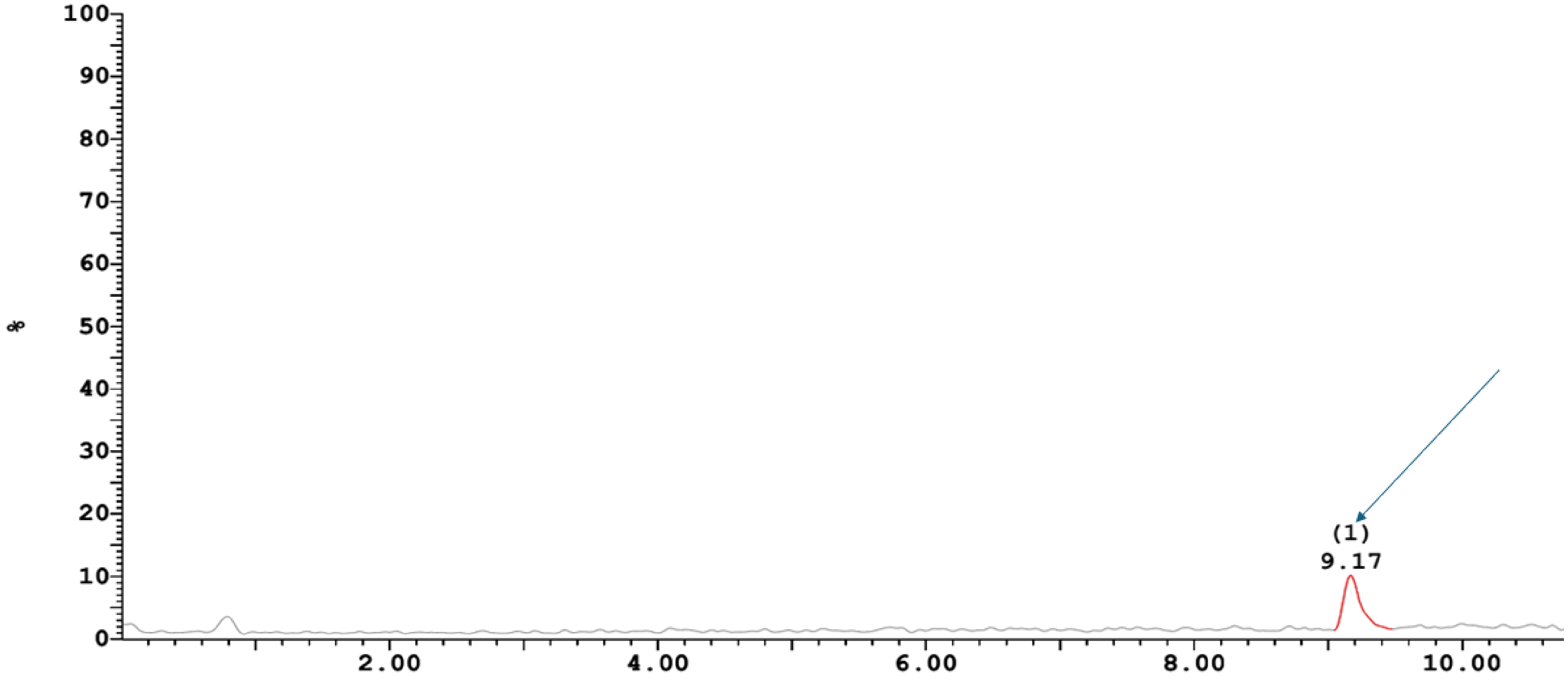

Fig. S9: LC-MS chromatogram of compound 1.

Sample Report (continued):

Peak ID	Time
1	9.17

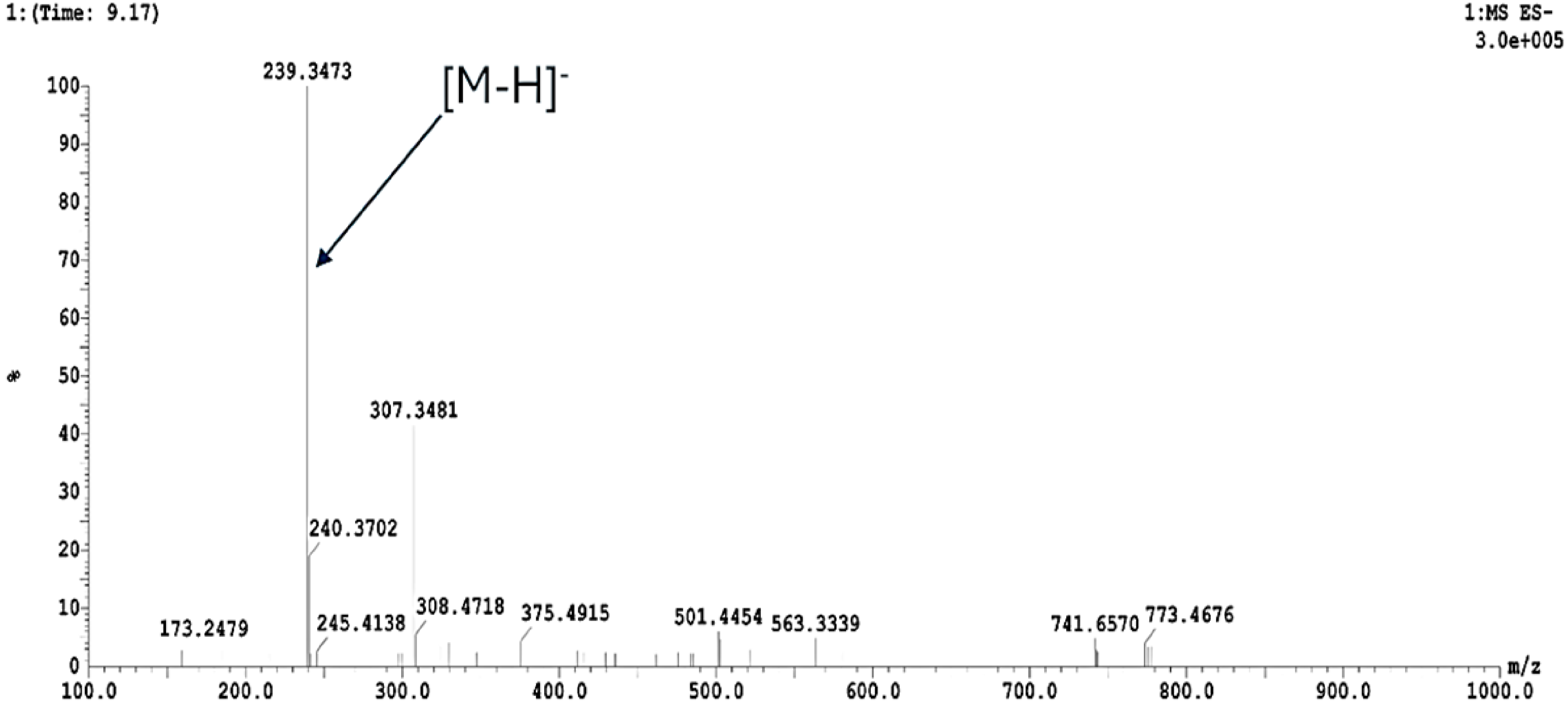

Fig. S10: LR-MS spectrum (-ve) of compound 1.

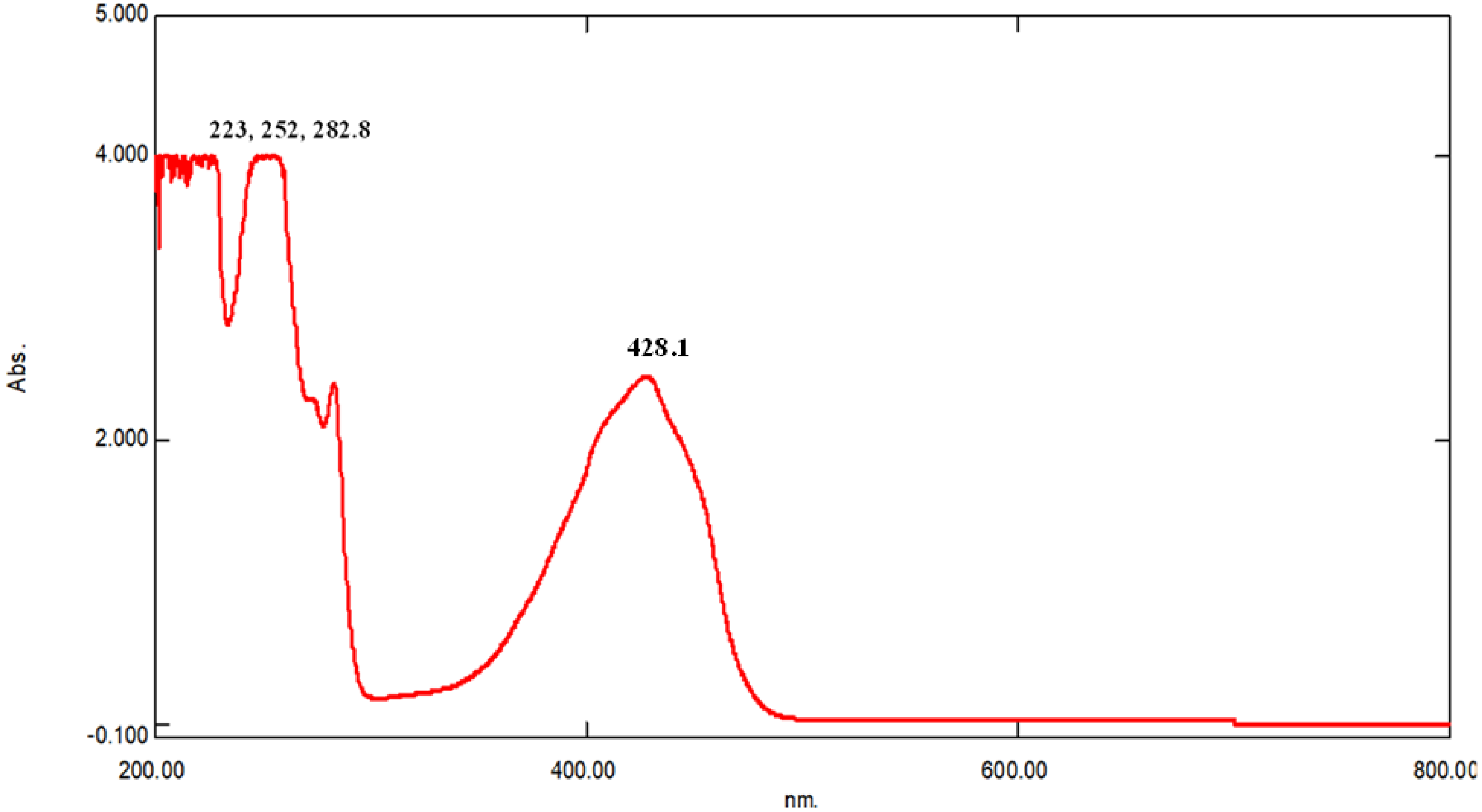

Fig. S11: UV spectrum of compound 1 (MeOH).

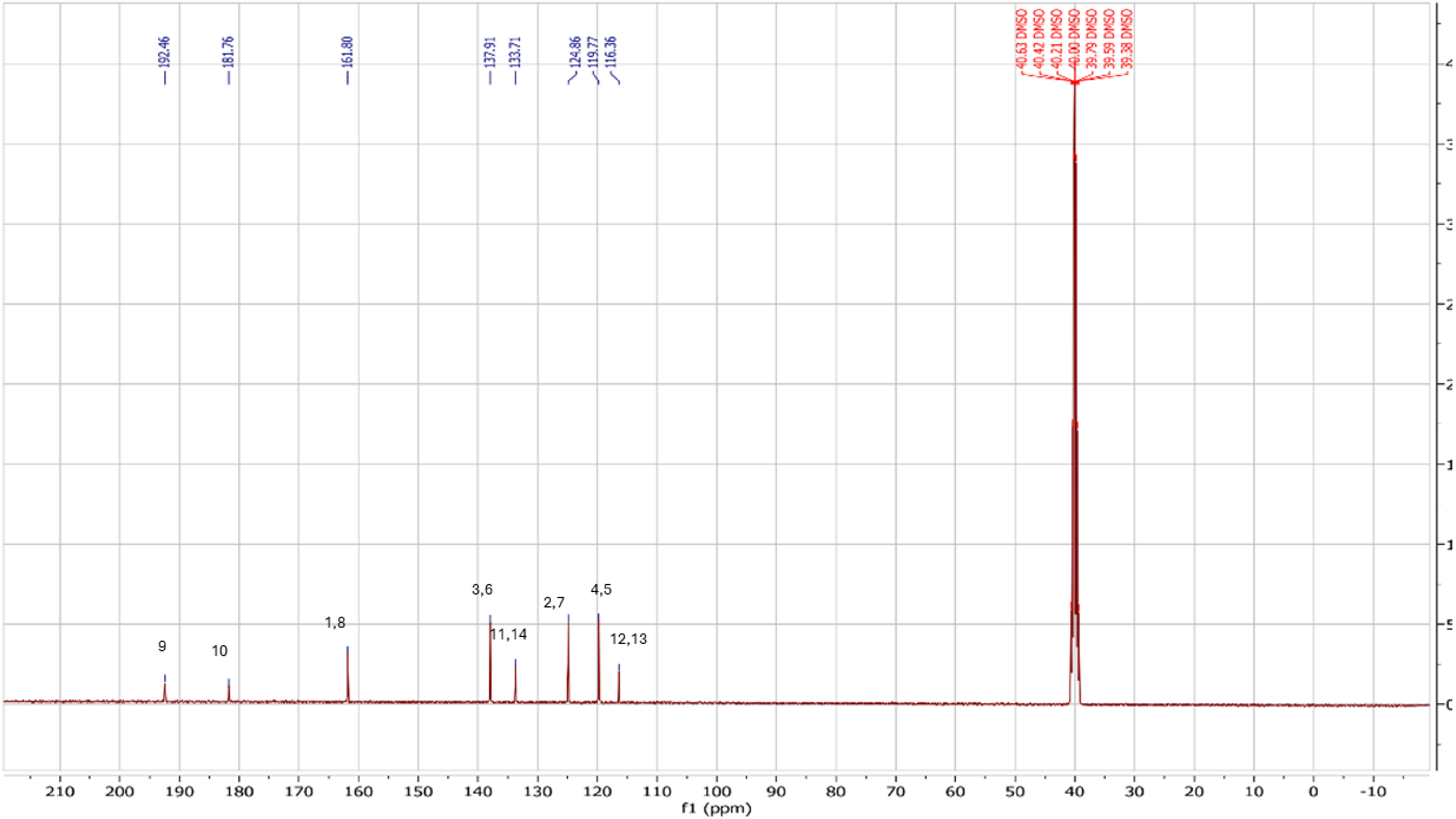
192.46
181.76
161.80
137.91
133.71
124.86
119.77
116.36
40.63 DMSO
40.42 DMSO
40.21 DMSO
40.00 DMSO
39.79 DMSO
39.59 DMSO
39.38 DMSO
9
10
1,8
3,6
11,14
2,7
4,5
12,13
210 200 190 180 170 160 150 140 130 120 110 100 90 80 70 60 50 40 30 20 10 0 -10
f1 (ppm)

Fig. S12: ^{13}C NMR spectrum of compound 1 (DMSO-d_6, 100.6 MHz).

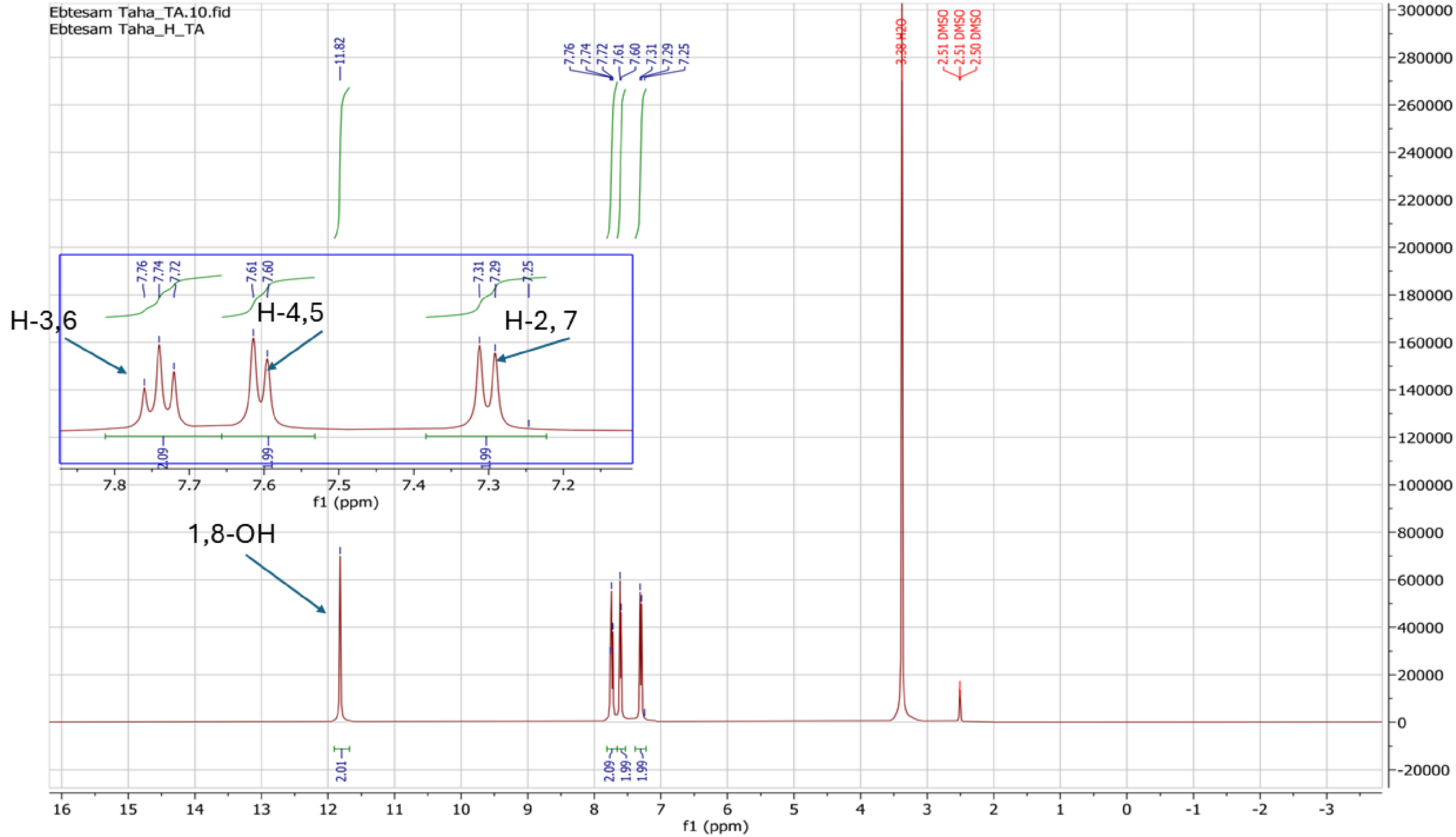

Fig. S 13: ^{1}H NMR spectrum of compound 1 (DMSO-d_6, 400 MHz).

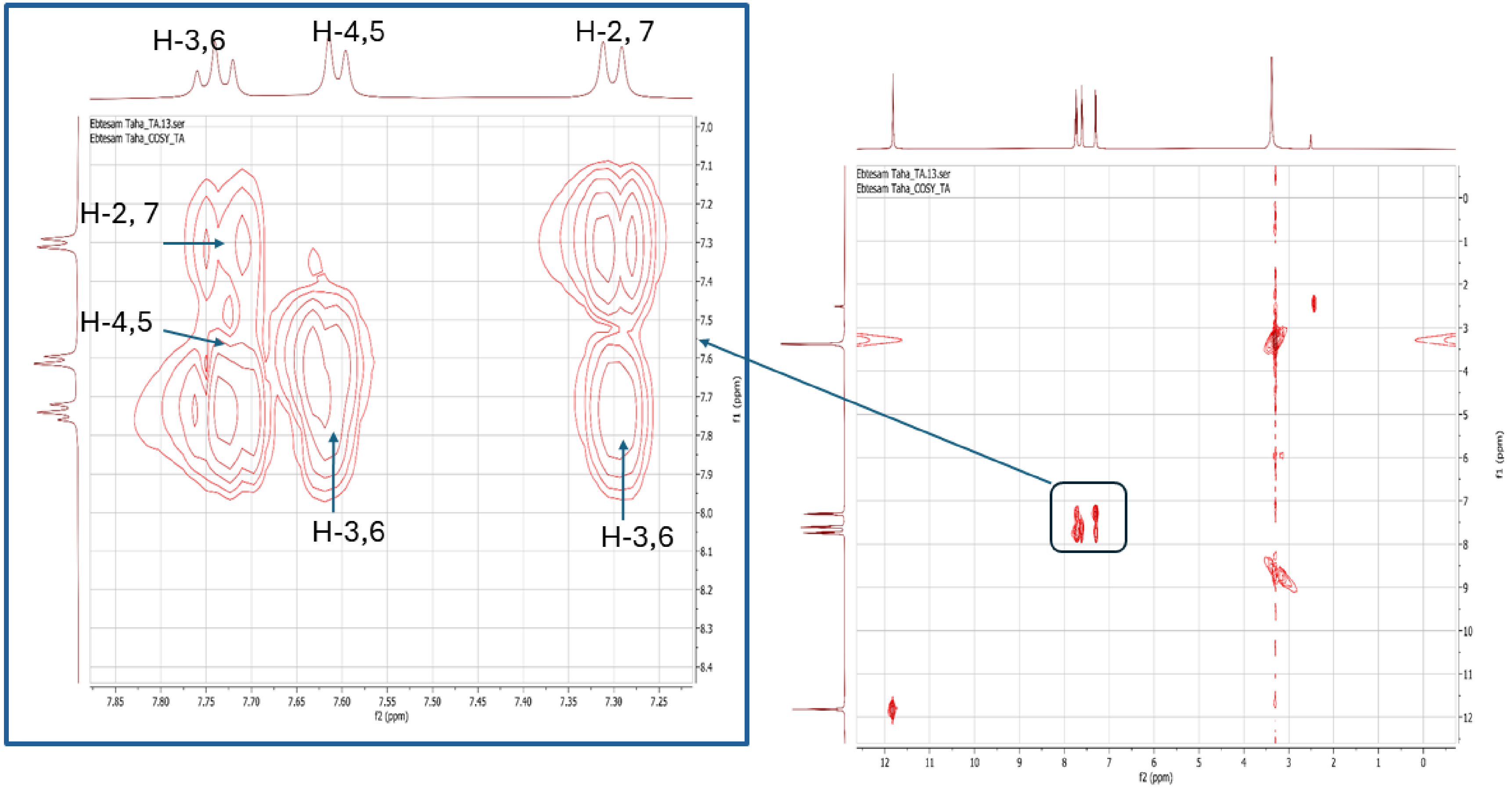

Fig. S14: COSY spectrum of compound 1 (DMSO-d_6).

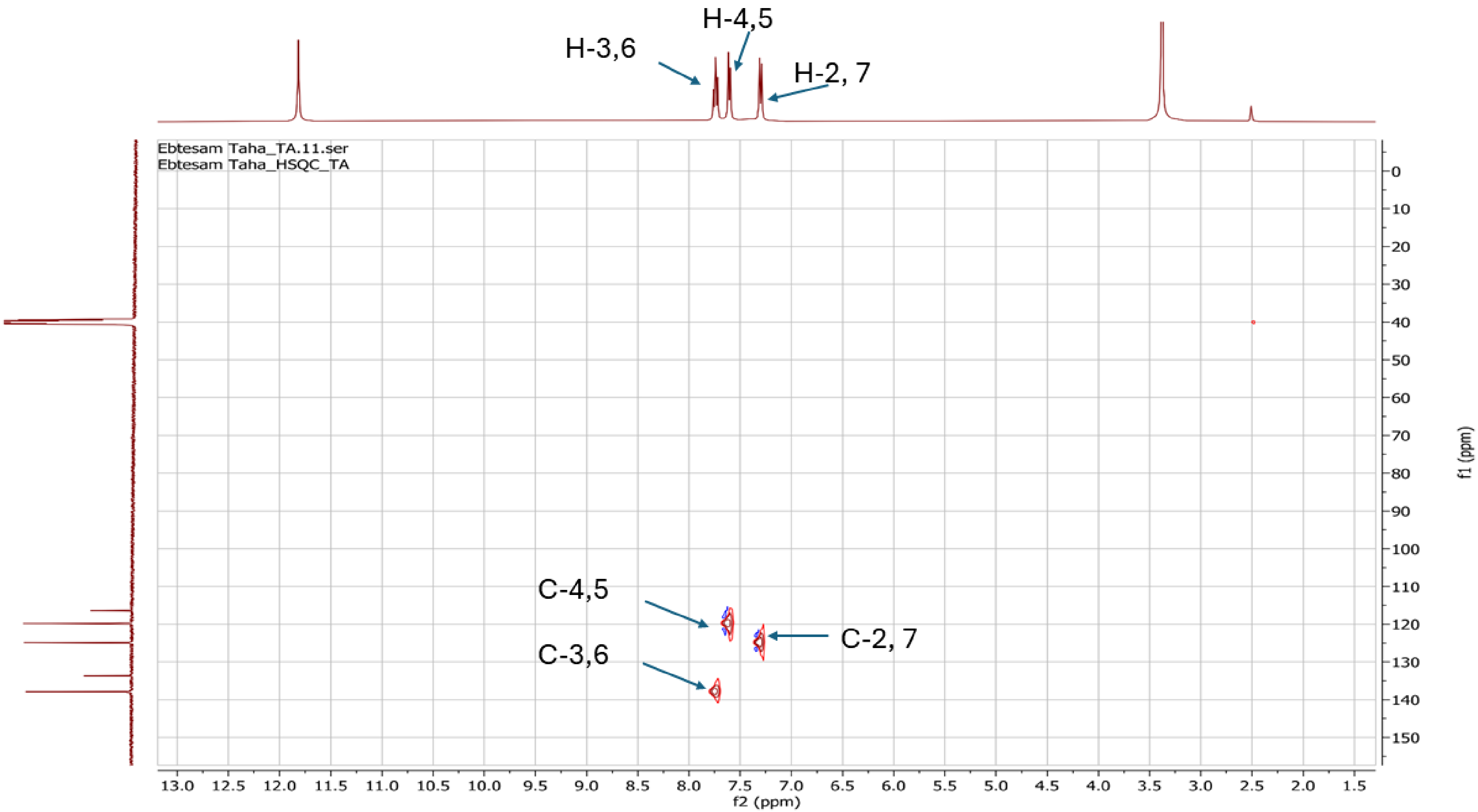

Fig. S15: HSQC spectrum of compound 1 (DMSO-d_6).

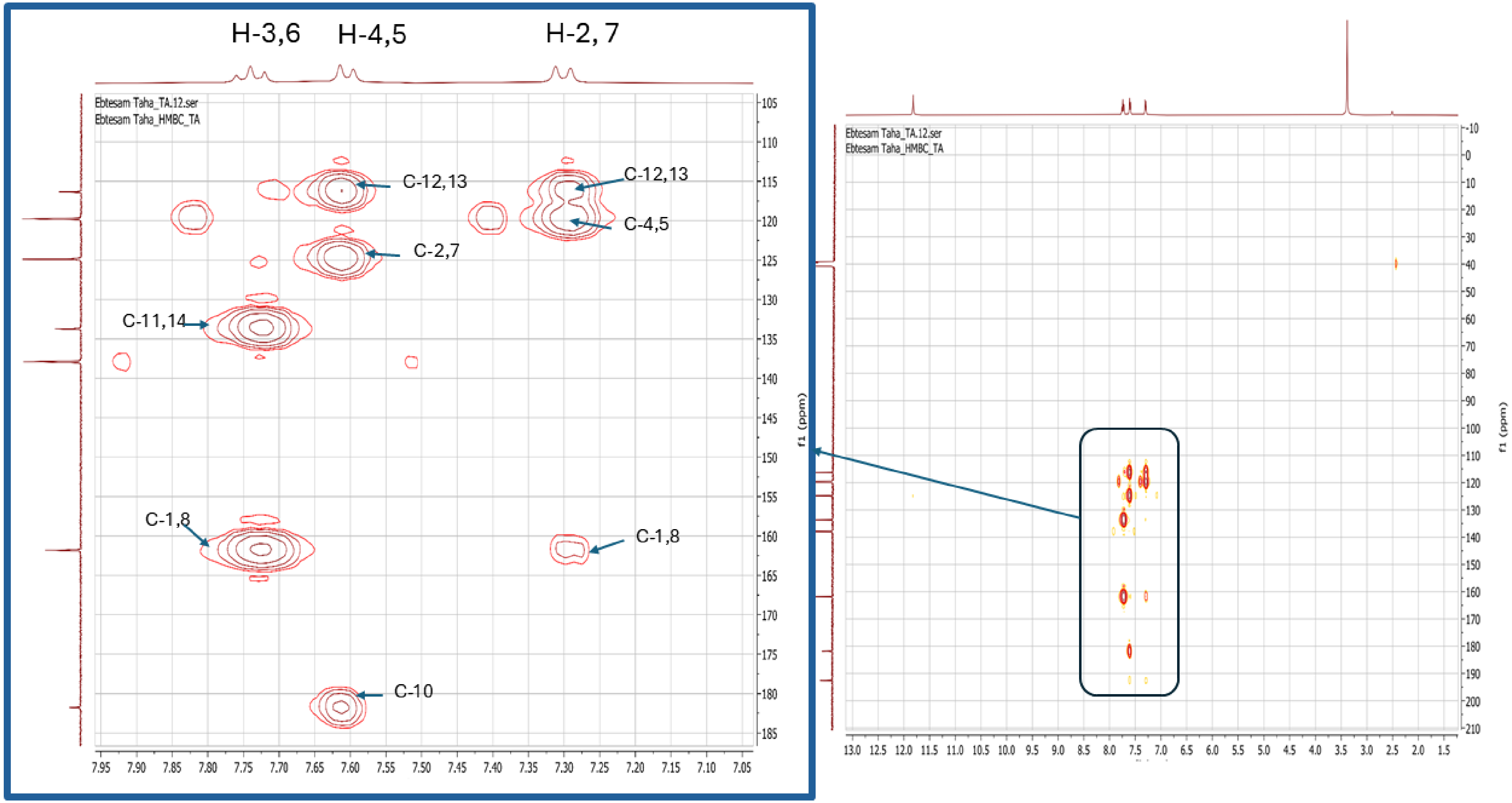

Fig. S16: HMBC spectrum of compound 1 (DMSO-d_6).

Compound 2:

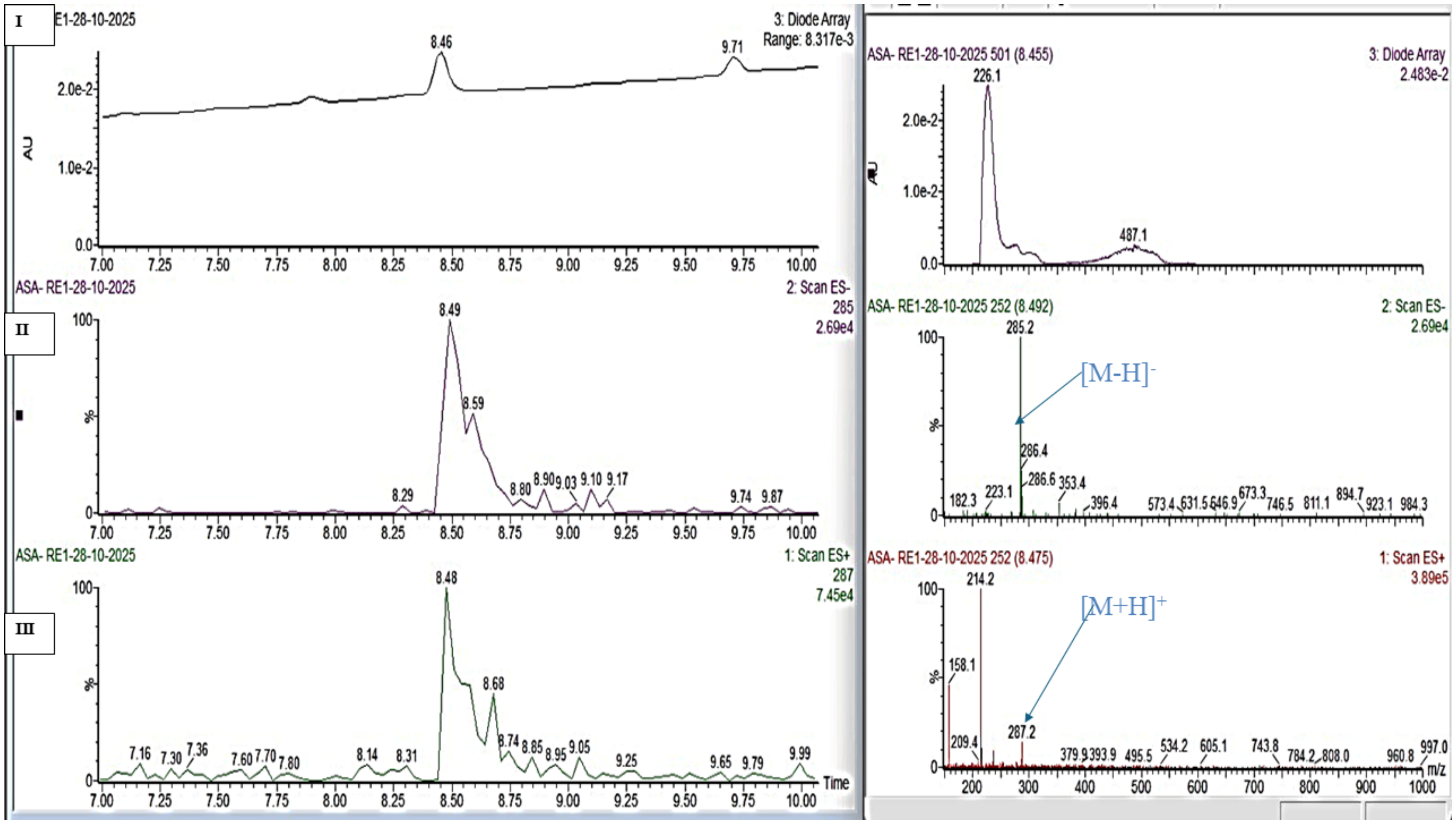

Fig. S17: LC-MS total ion chromatogram of compound 2. (I) Diode array spectrum (left panel) and UV spectrum (right panel). (II) In negative ion mode (left panel) and mass spectrum (right panel). (III) In positive ion mode (left panel) and MS spectrum (right panel).

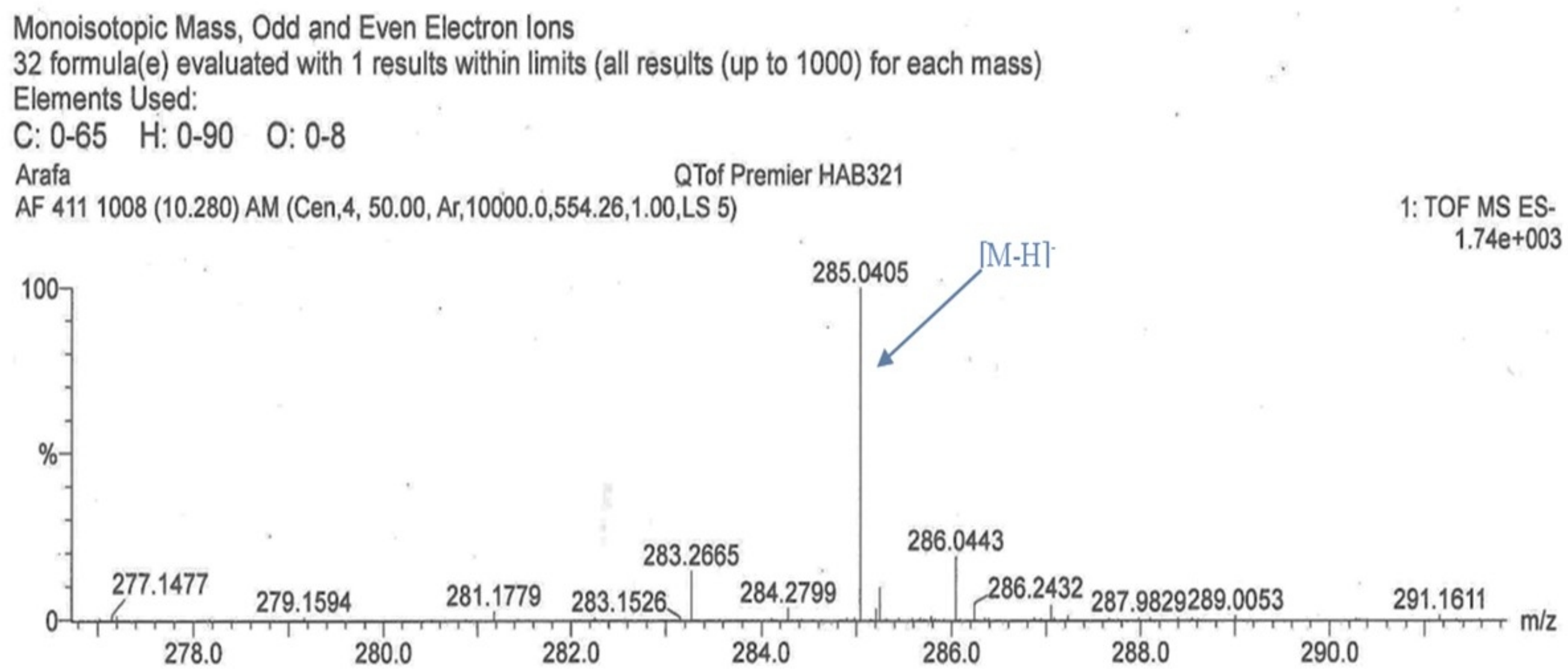

Fig. S18: HRESI-MS (-ve) of compound 2.

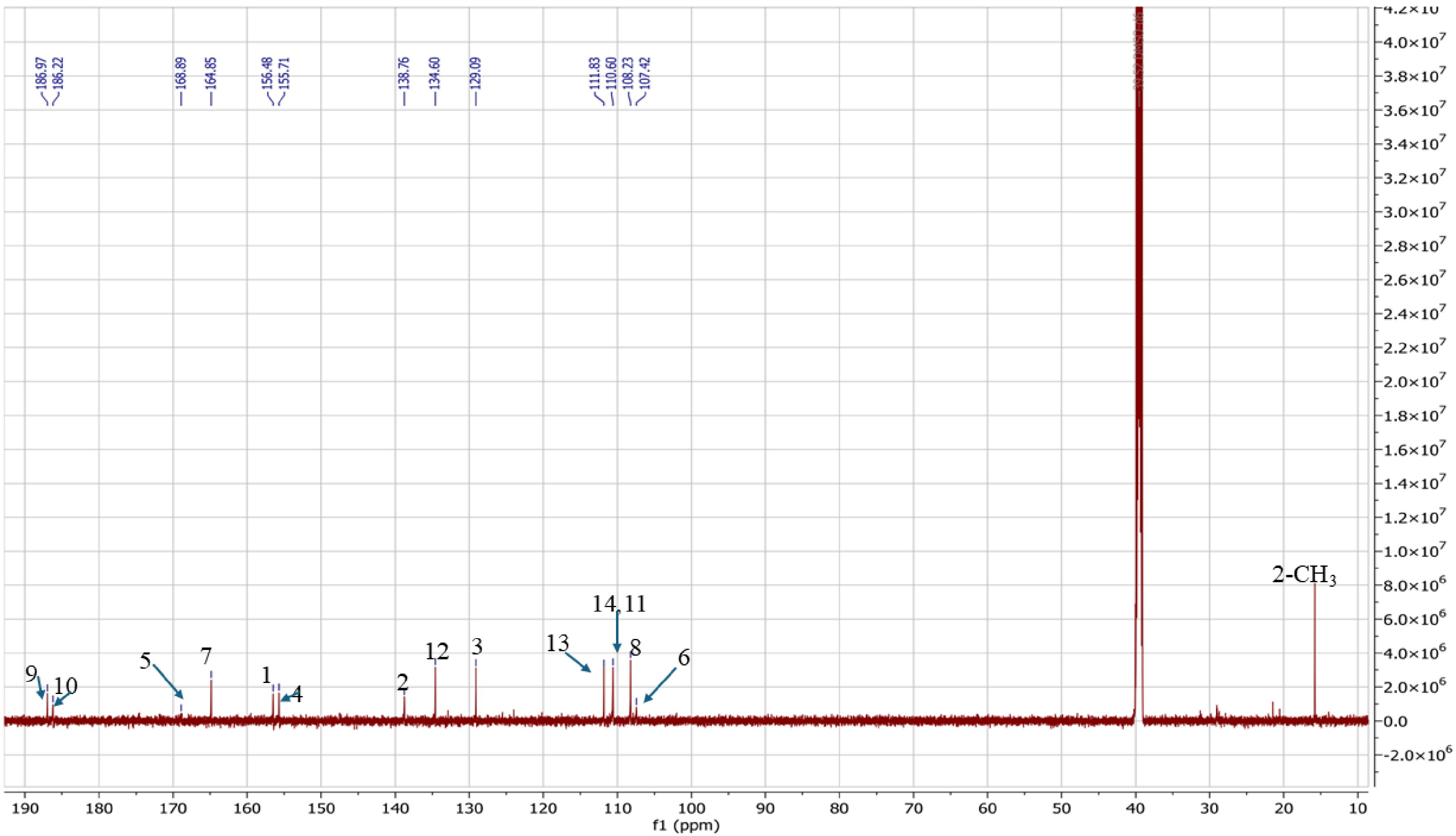

Fig. S19: ^{13}C NMR spectrum of compound 2 (DMSO-d$_6$).

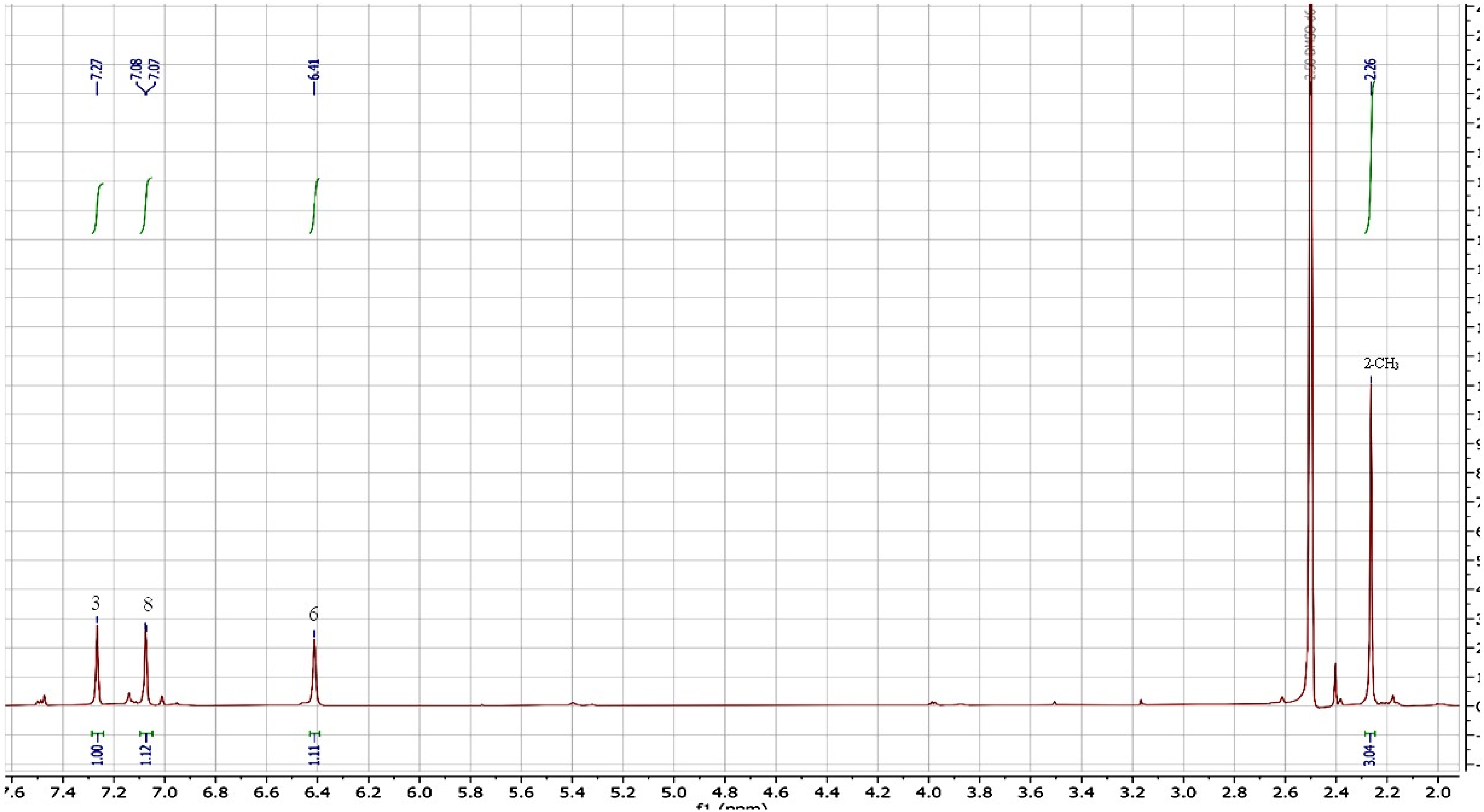

Fig. S20: ^{1}H NMR spectrum of compound 2 (DMSO-d_6, 600 MHz) after water suppression.

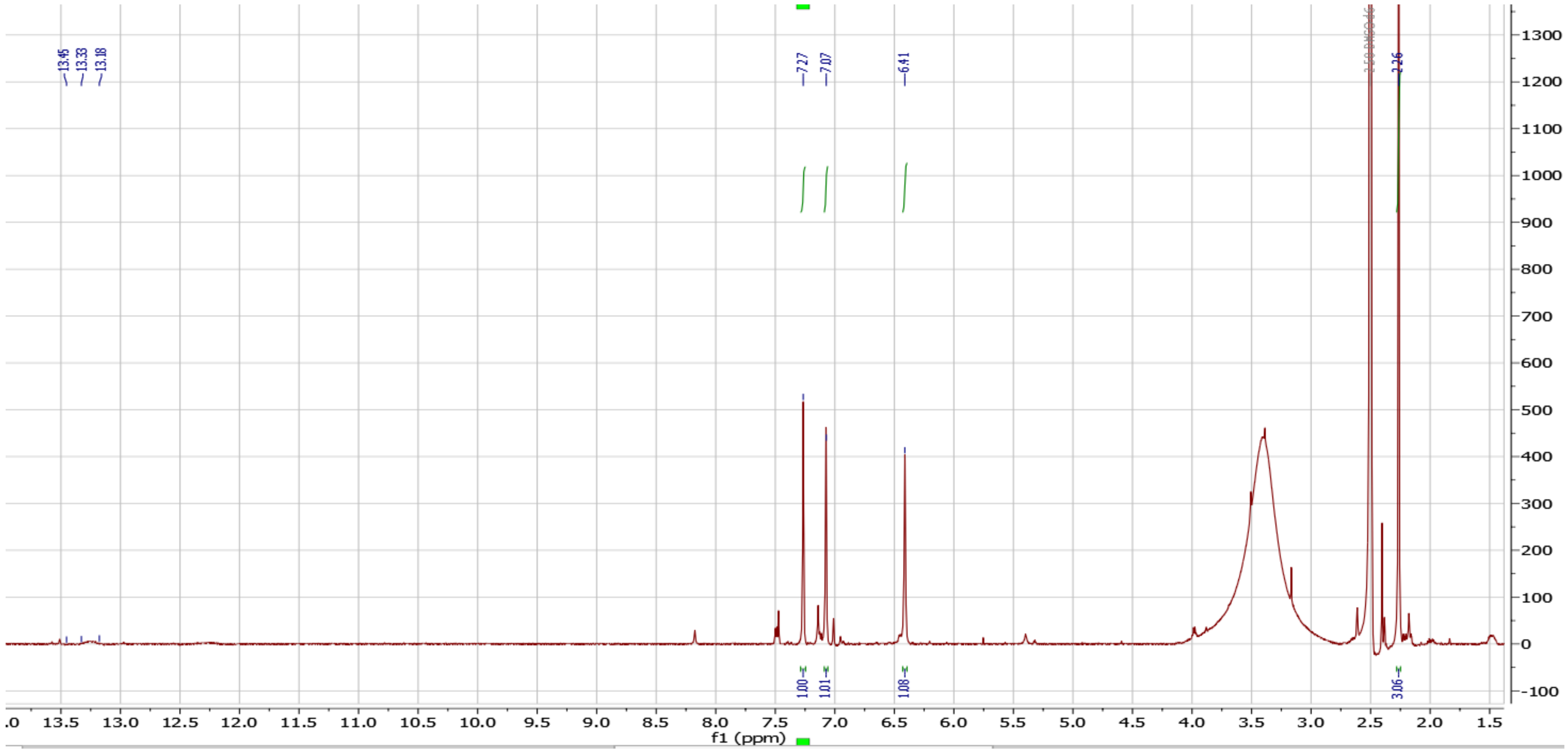

Figure S20 (Continue): ^{1}H NMR spectrum of compound 2 (DMSO-d_6, 600 MHz) before water suppression.

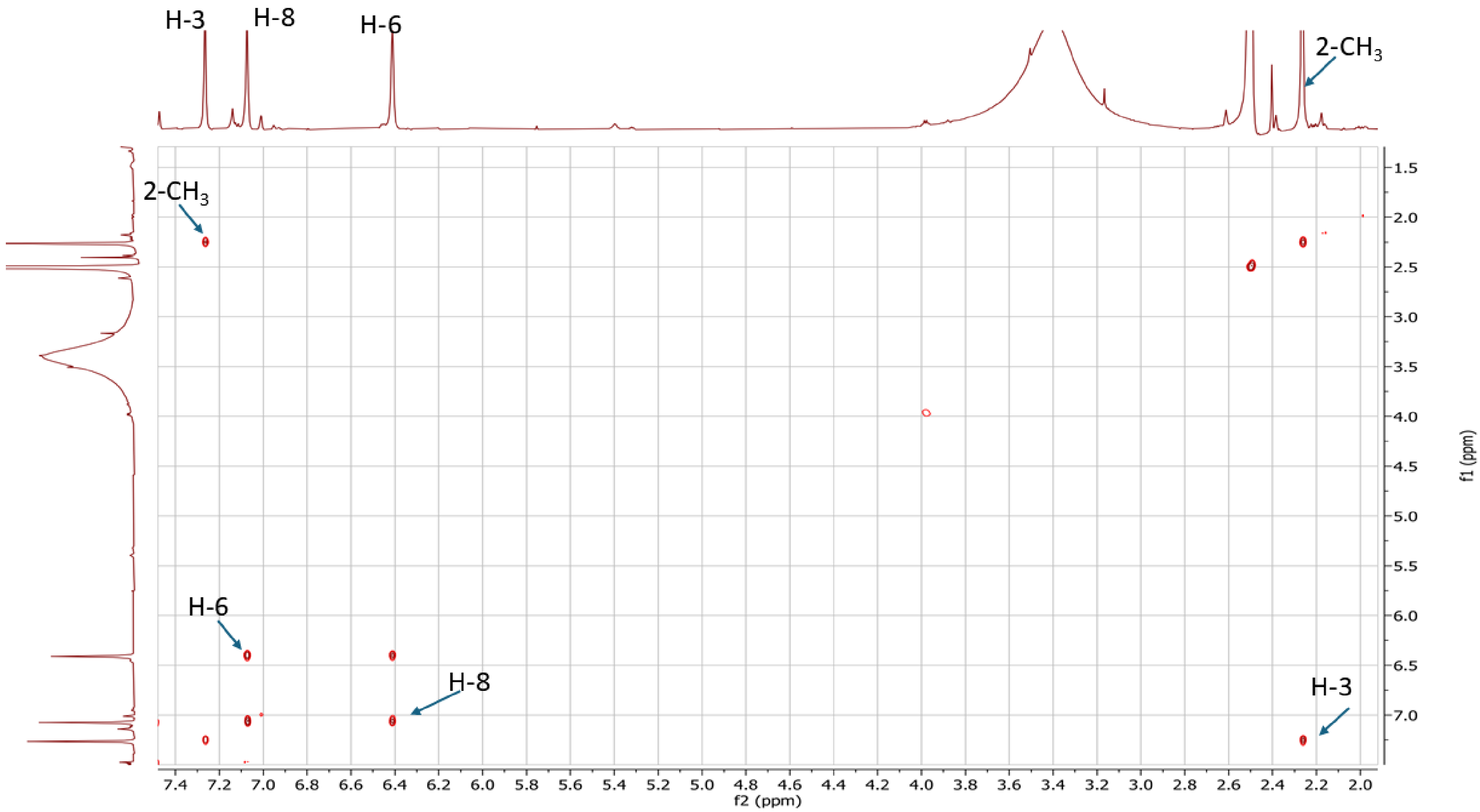

Fig. S21: COSY spectrum of compound 2 (DMSO-d_6).

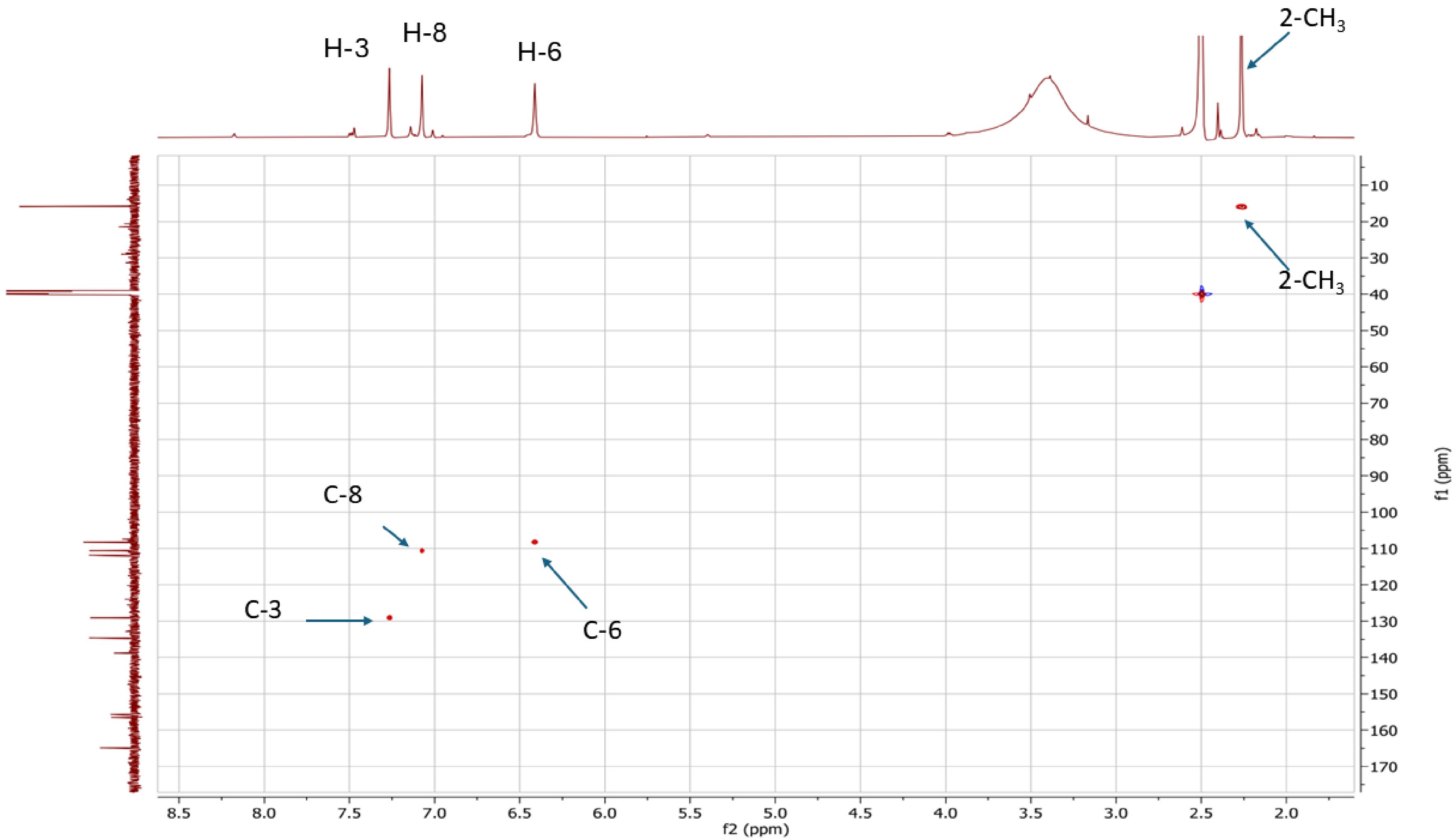

Fig. S22: HSQC spectrum of compound 2 (DMSO-d_6).

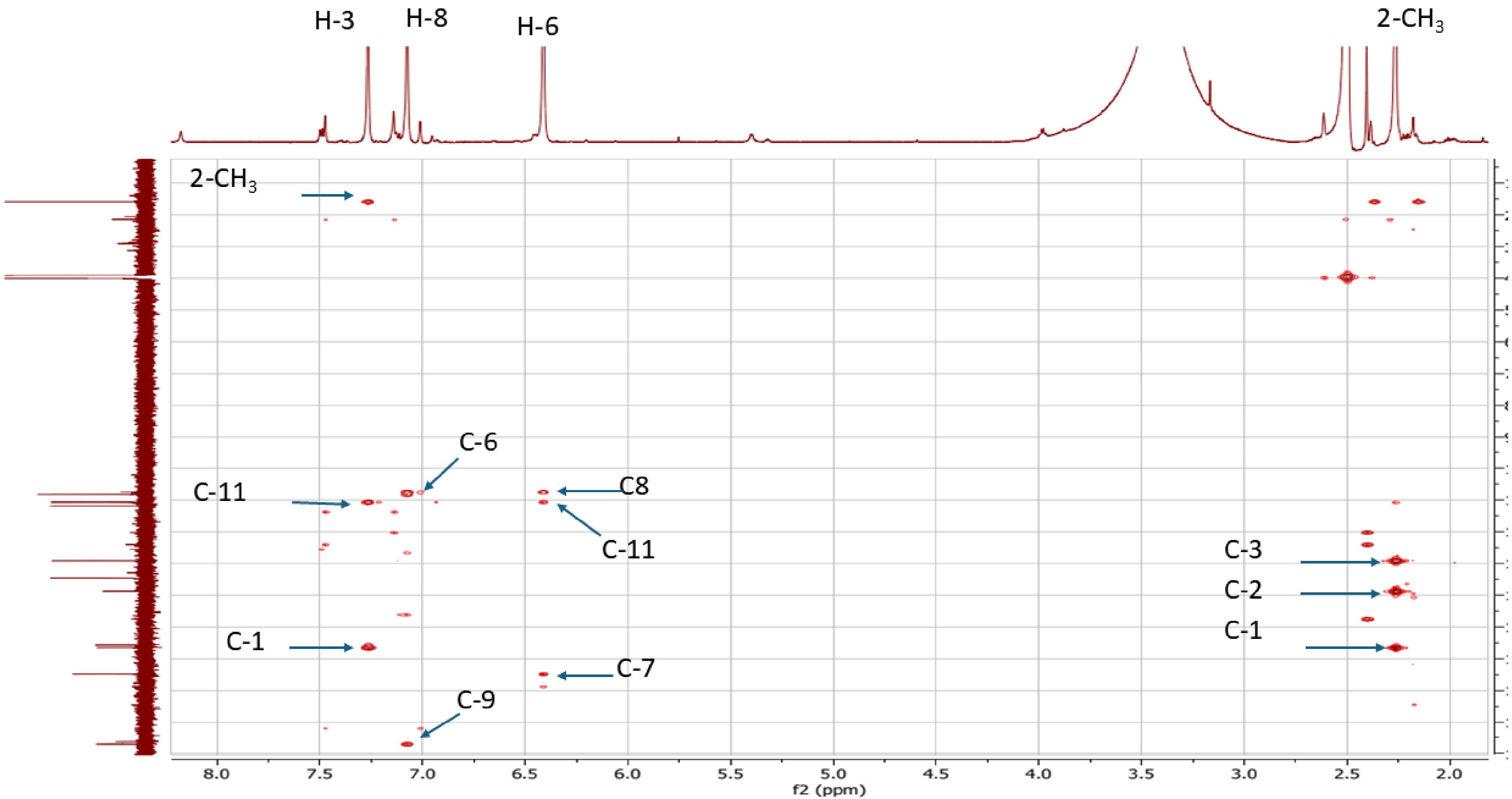

Fig. S23: HMBC spectrum of compound 2 (DMSO-d_6).

3A/3B Mixture

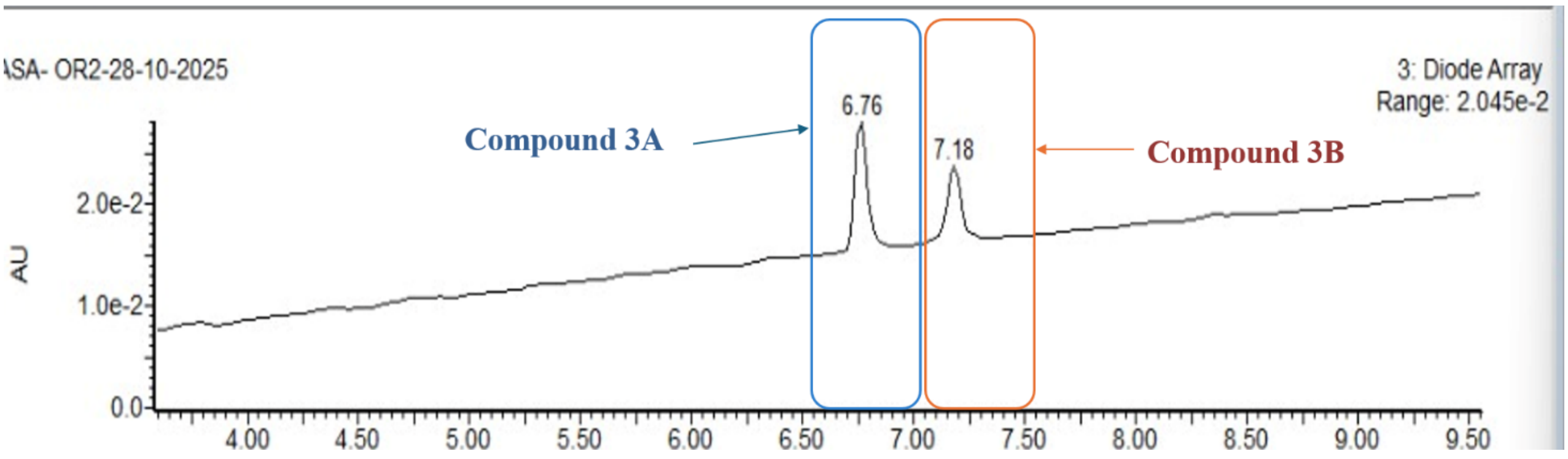

Fig. S24: Analytical LCMS (photodiode array detector) analysis of compound 3A (in blue color) and 3B (in orange color).

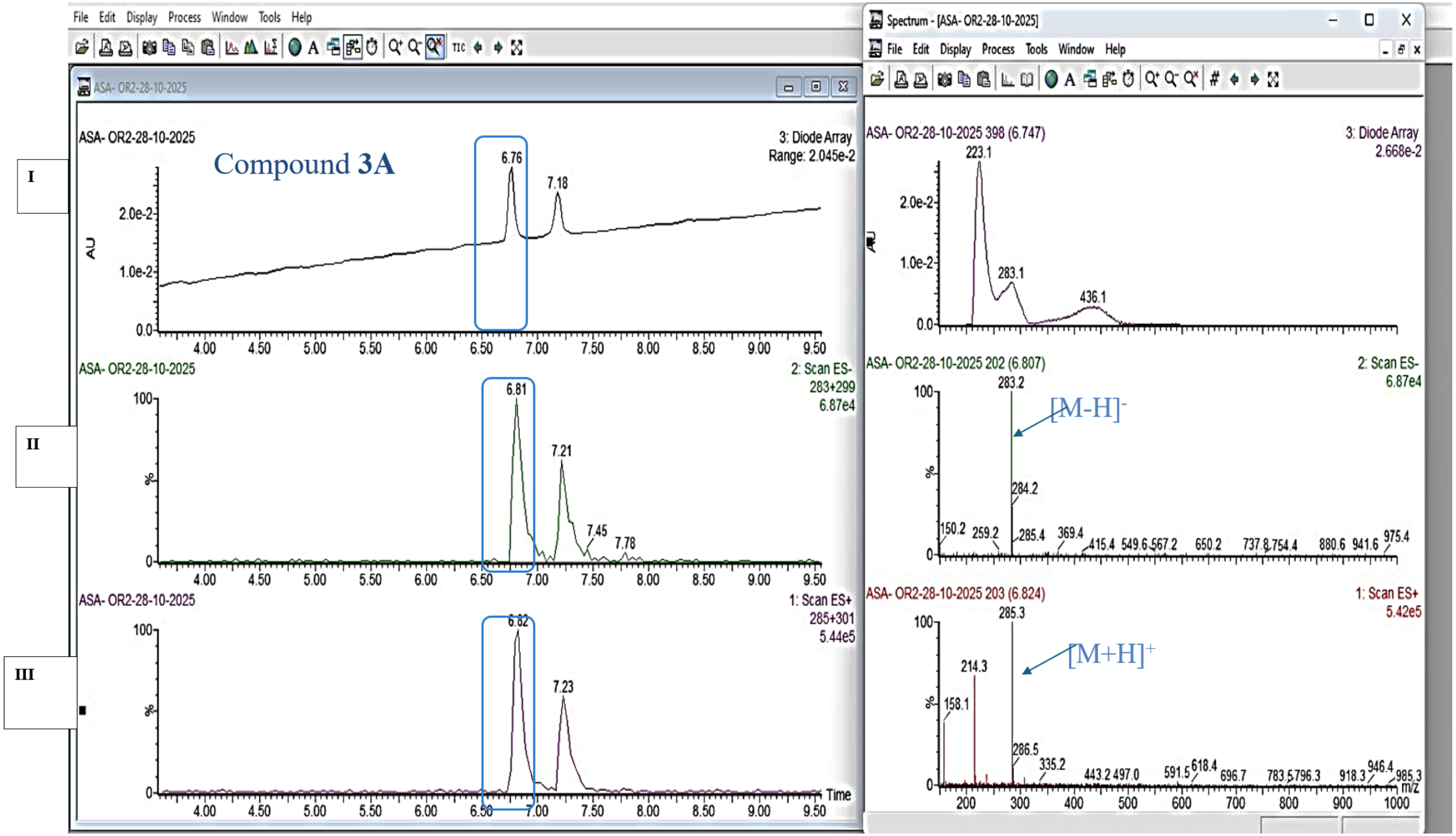

Fig. S25: LC-MS total ion chromatogram of compound 3A. (I) Diode array spectrum (left panel) and UV spectrum (right panel). (II) In negative ion mode (left panel) and mass spectrum (right panel). (III) In positive ion mode (left panel) and MS spectrum (right panel).

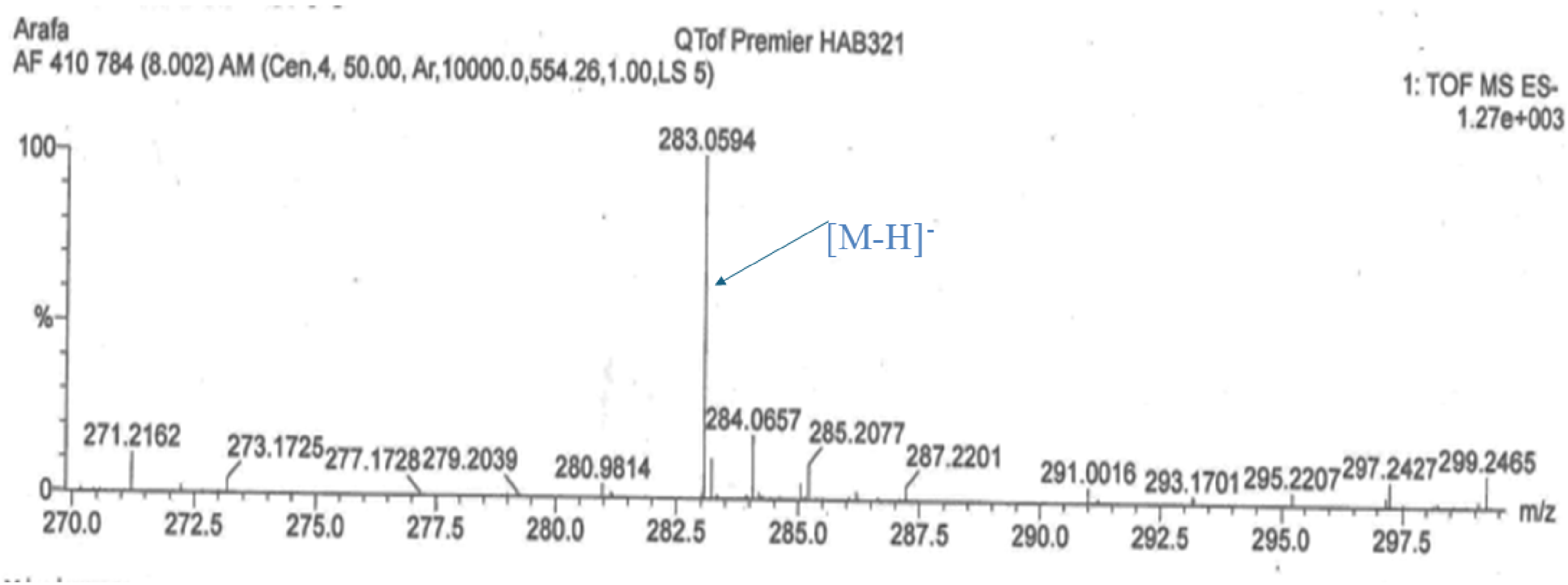

Fig. S26: HRESI-MS (-ve) of compound 3A.

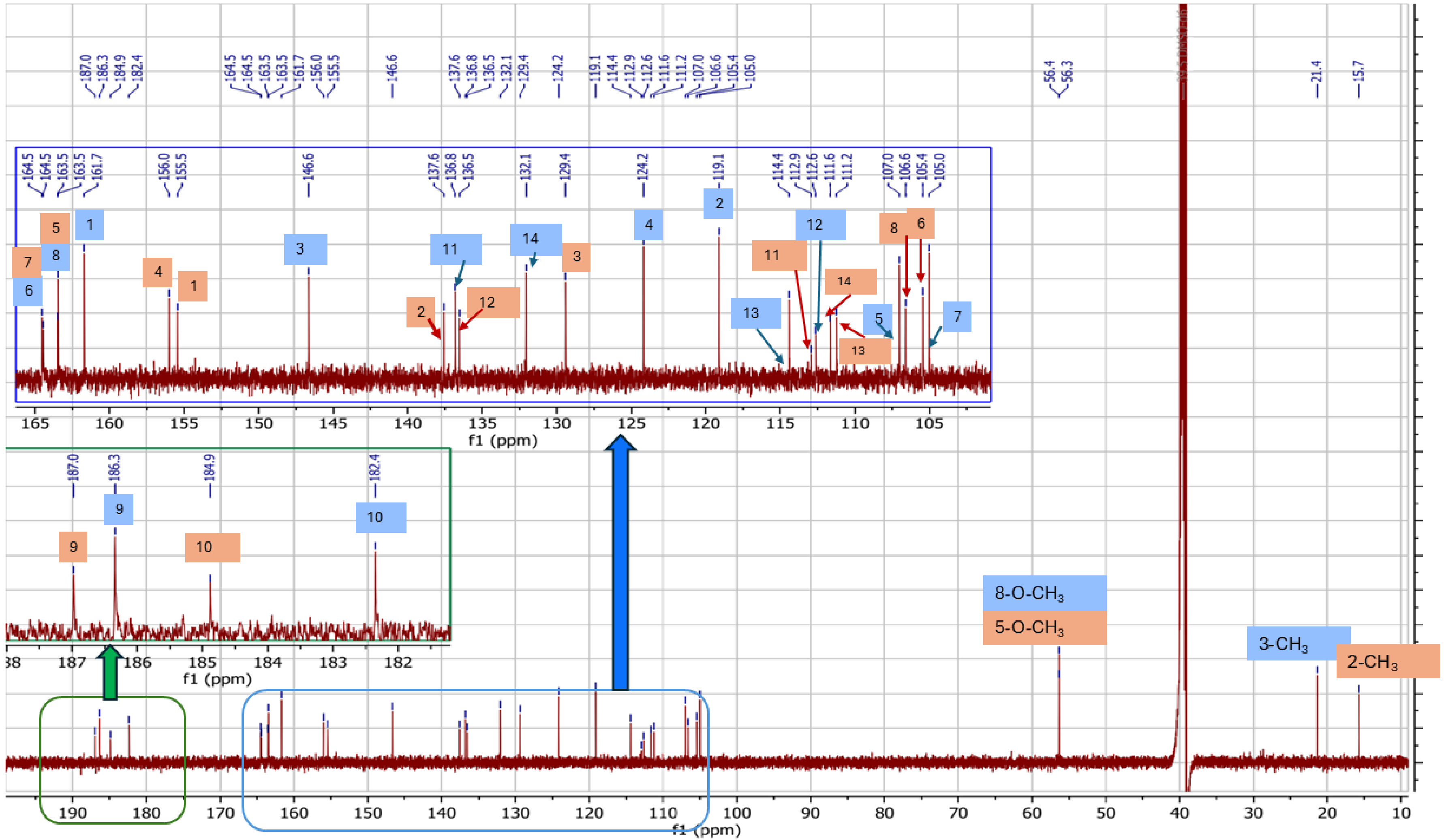

Fig. S27: ^{13}C NMR spectrum (DMSO-d$_6$, 151 MHz) of compound 3A (in blue color) and 3B (in orange color).

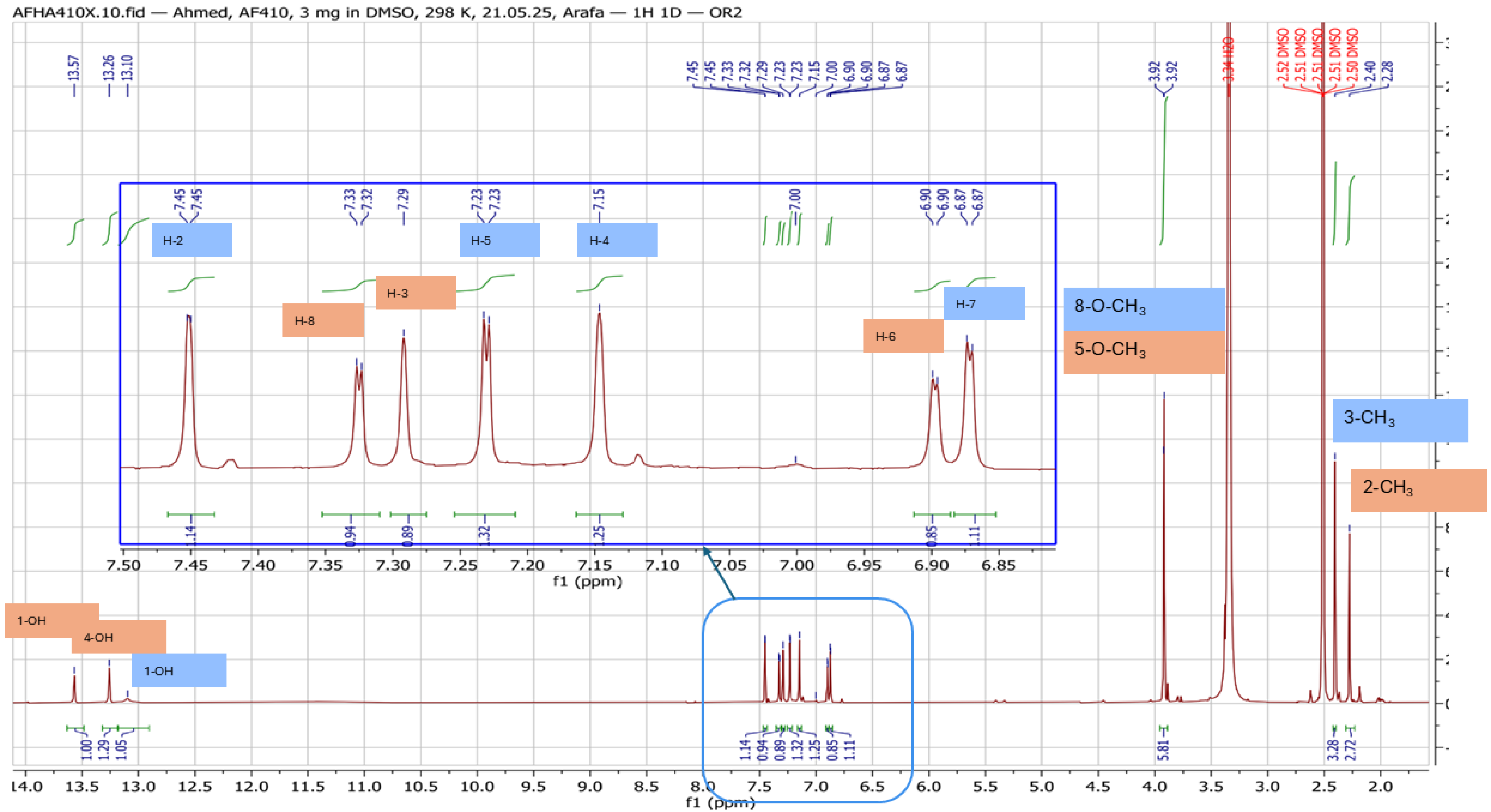

Fig. S28: ^{1}H NMR spectrum (DMSO-d_6, 600 MHz) of compound 3A (in blue color) and 3B (in orange color).

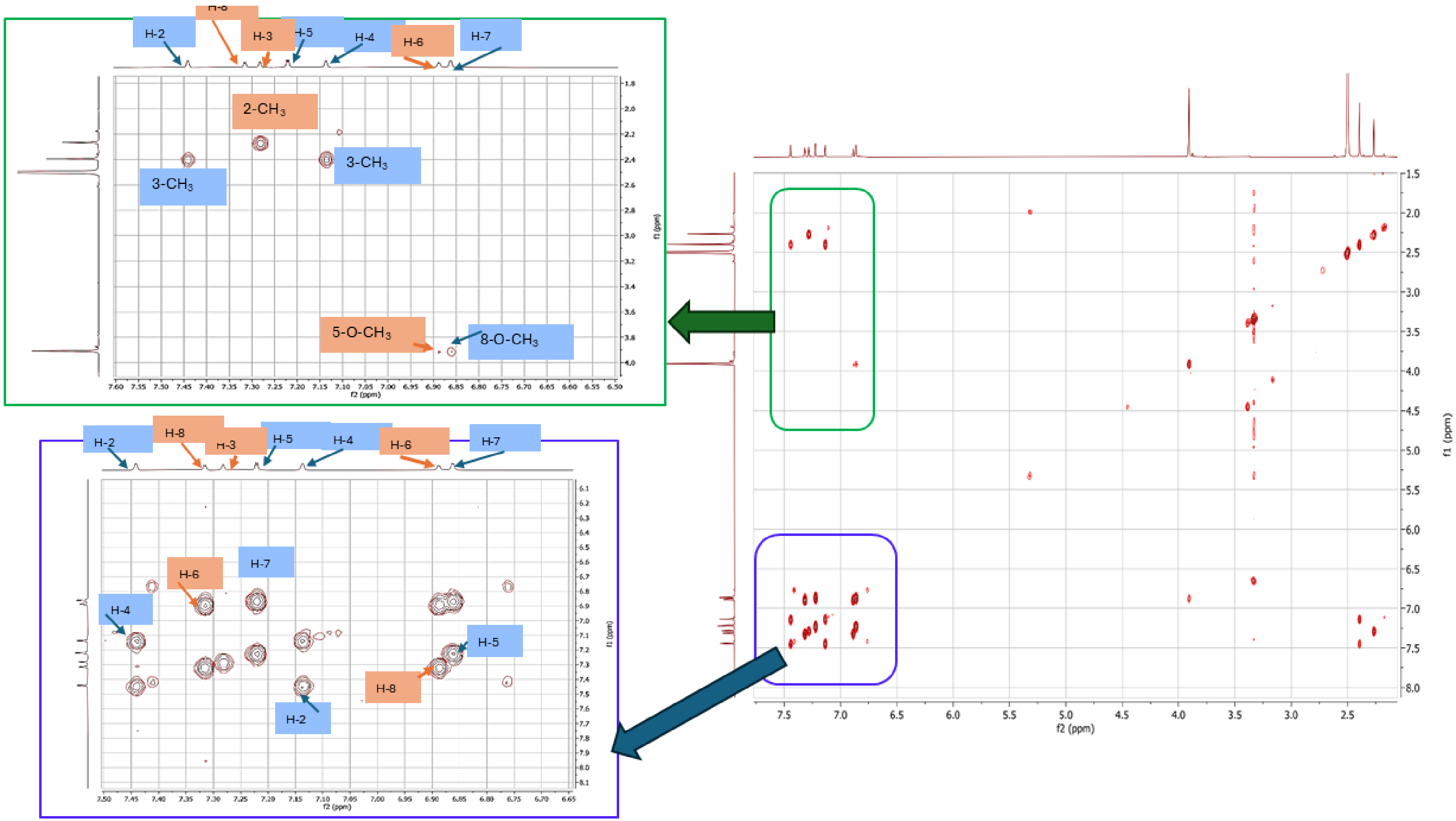

Fig. S29: COSY spectrum (DMSO-d_6) of compounds 3A (in blue color) and 3B (in orange color).

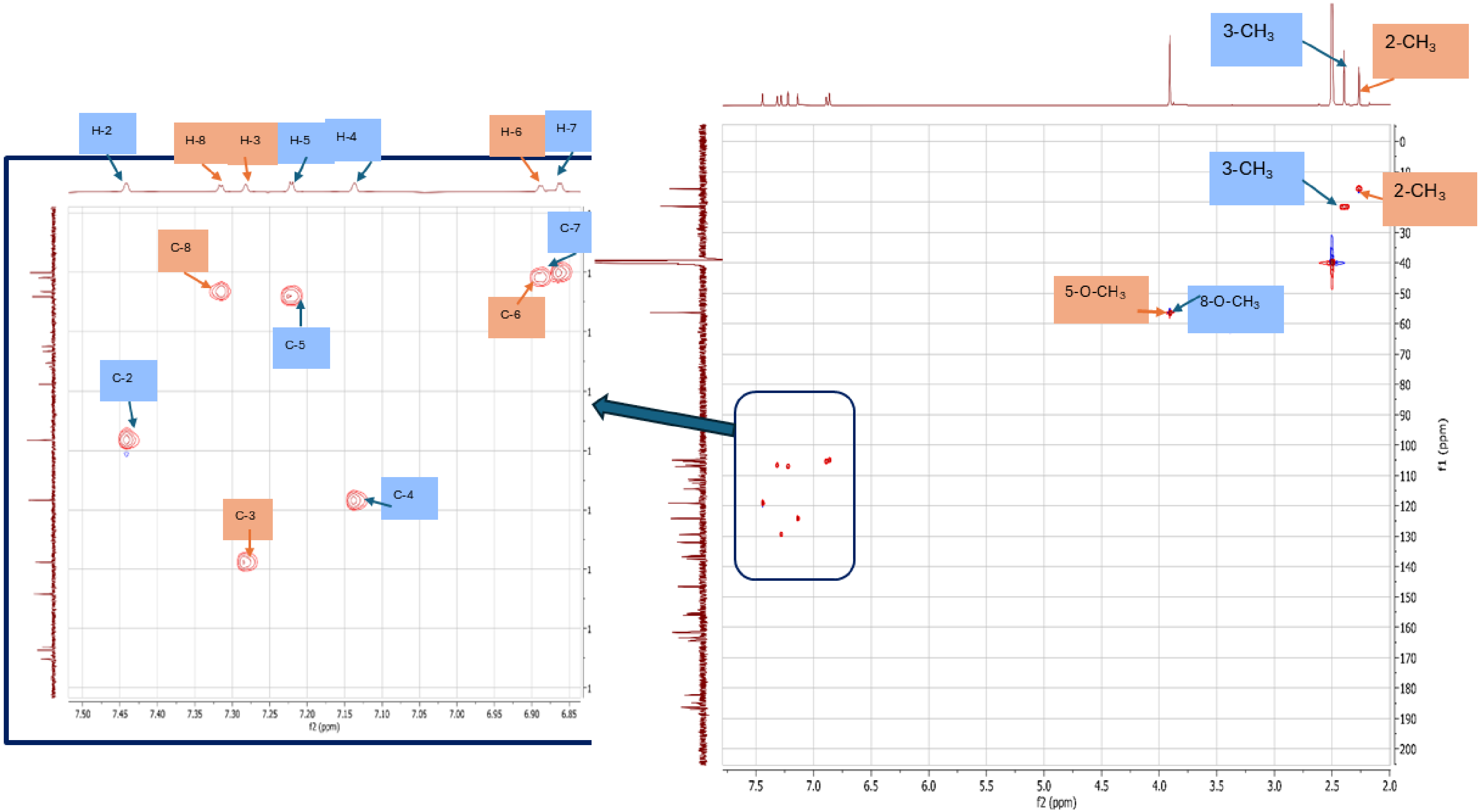

Fig. S30: HSQC spectrum (DMSO-d$_6$) of compounds 3A (in blue color) and 3B (in orange color).

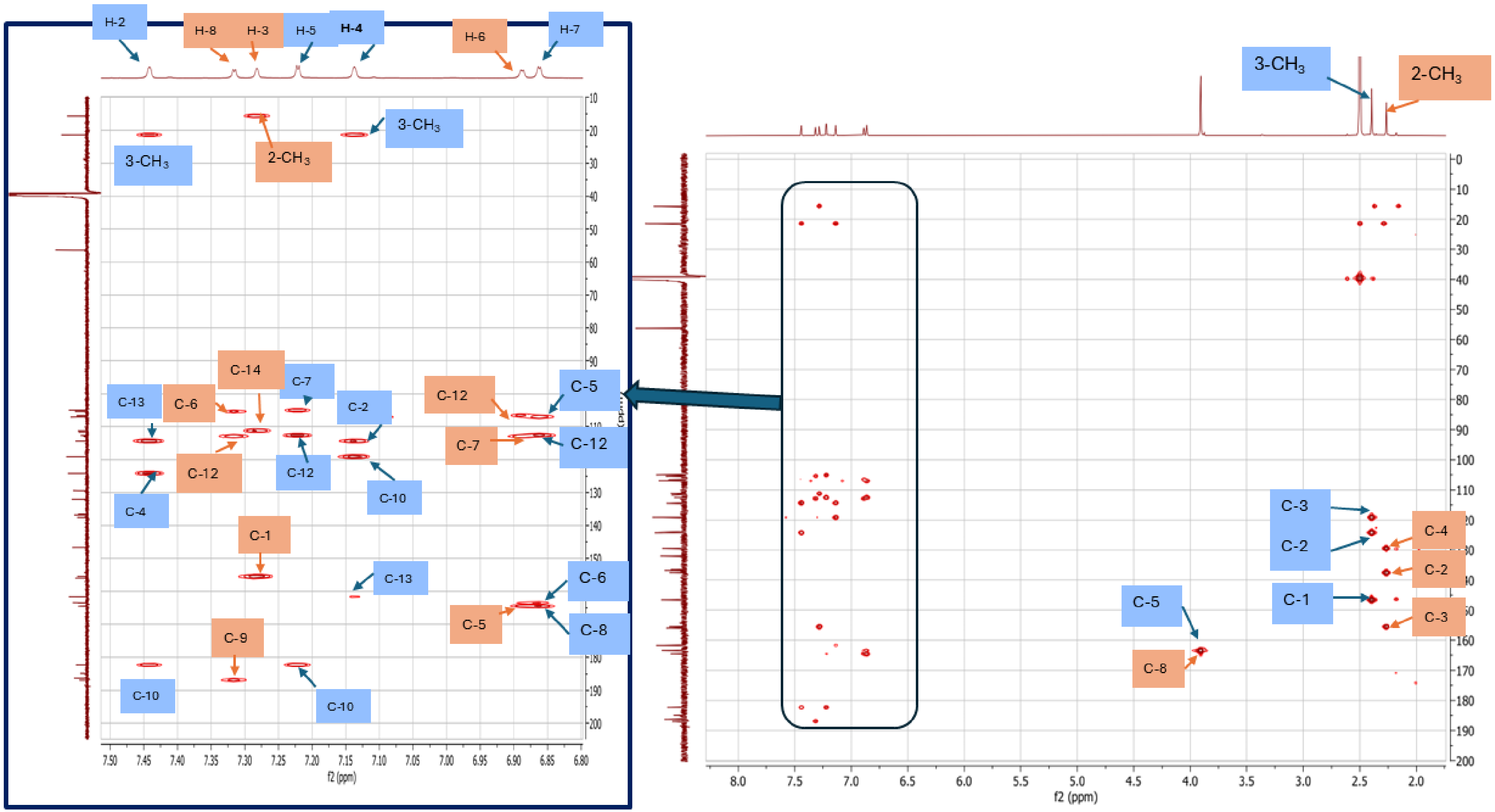

Fig. S31: HMBC spectrum (DMSO-d$_6$) of compounds 3A (in blue color) and 3B (in orange color).

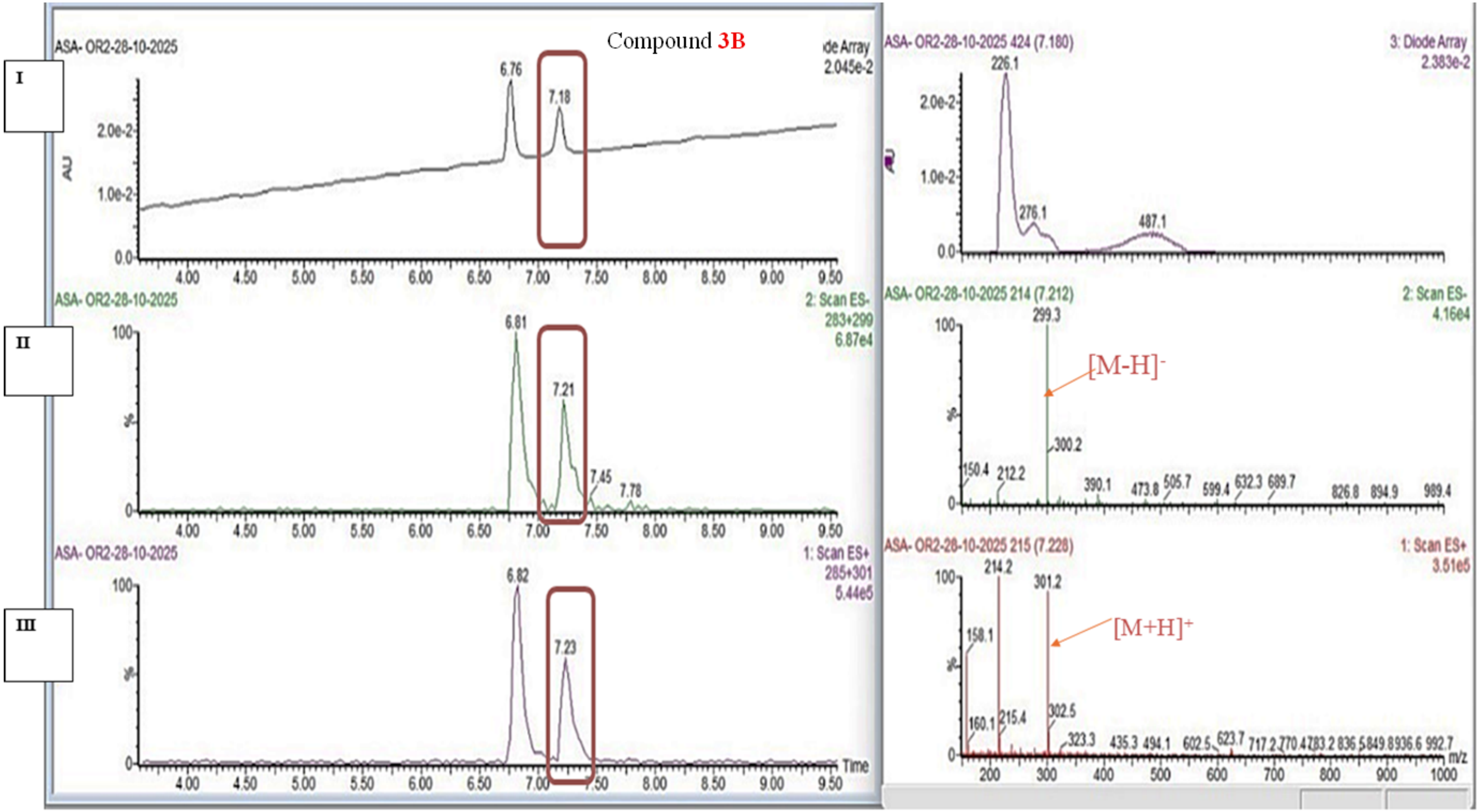

Fig. S32: LC-MS total ion chromatogram of compound 3B. (I) Diode array spectrum (left panel) and UV spectrum (right panel). (II) In negative ion mode (left panel) and mass spectrum (right panel). (III) In positive ion mode (left panel) and MS spectrum (right panel).

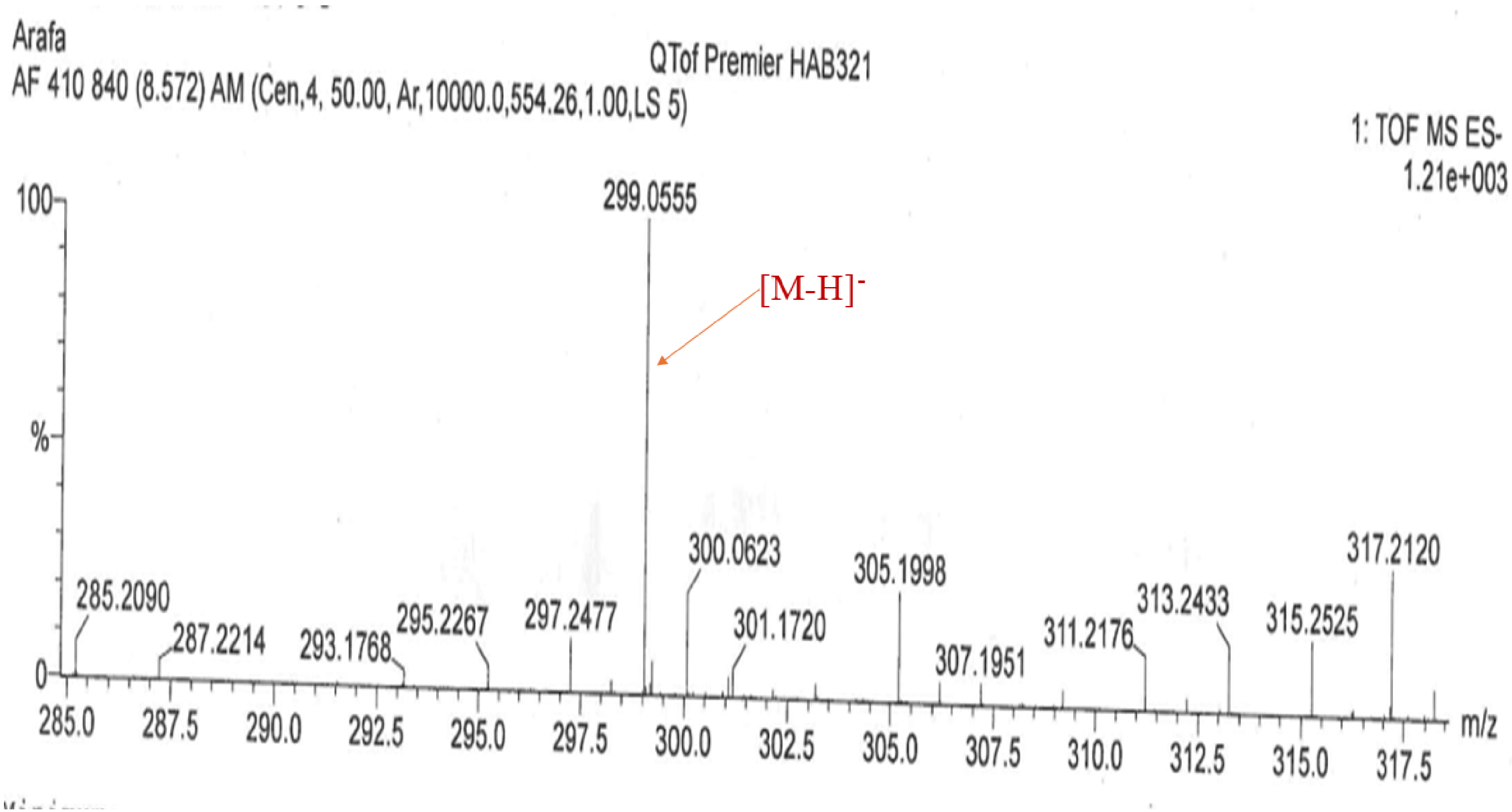

Fig. S33: HRESI-MS (-ve) of compound 3B.

Compound 4

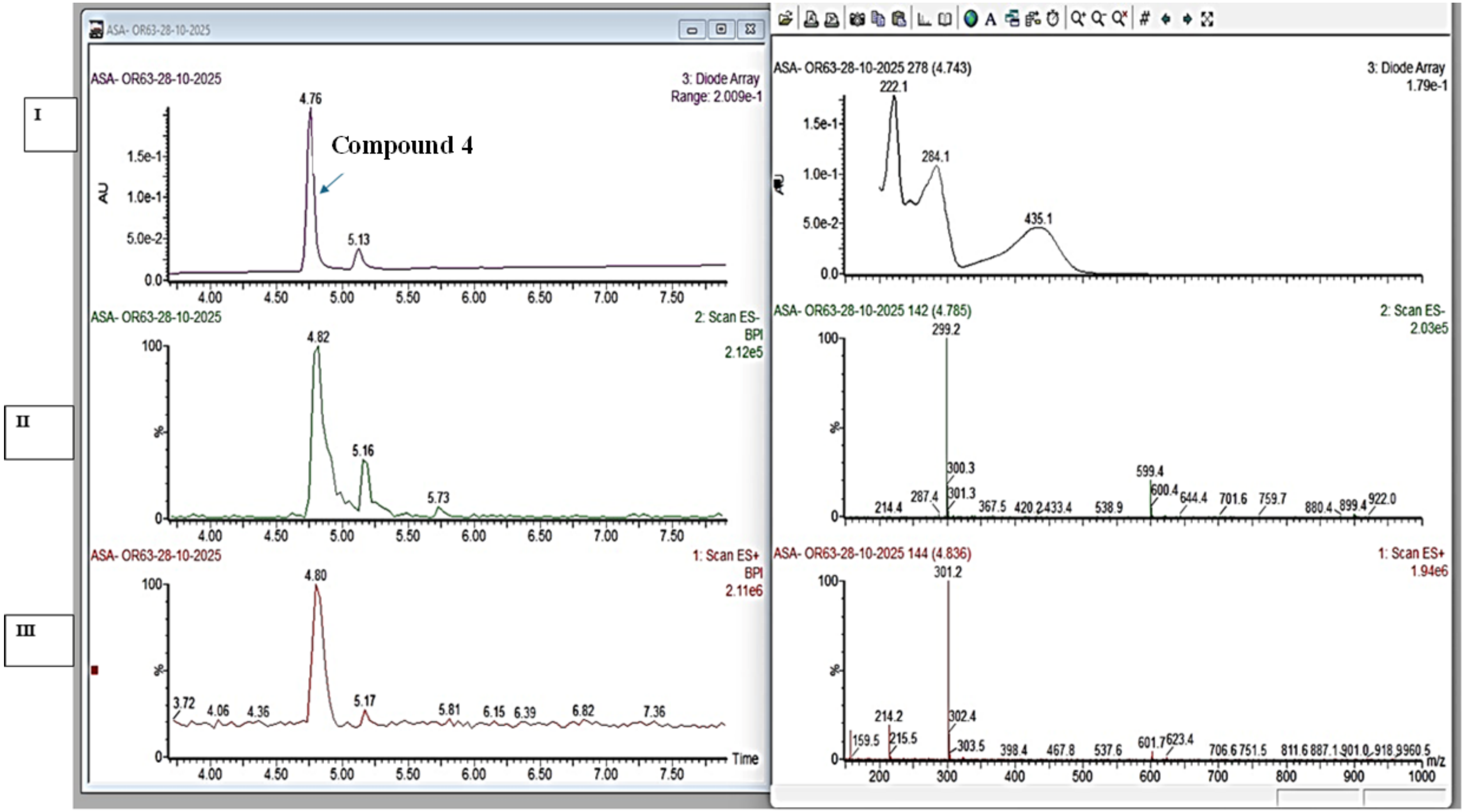

Fig. S34: LC-MS total ion chromatogram of compound 4. (I) Diode array spectrum (left panel) and UV spectrum (right panel). (II) In negative ion mode (left panel) and mass spectrum (right panel). (III) In positive ion mode (left panel) and MS spectrum (right panel).

Single Mass Analysis
Tolerance = 20.0 PPM / DBE: min = -1.5, max = 50.0
Element prediction: Off
Number of isotope peaks used for i-FIT = 3

Monoisotopic Mass, Odd and Even Electron Ions
25 formula(e) evaluated with 1 results within limits (all results (up to 1000) for each mass)
Elements Used:
C: 0-45 H: 0-60 O: 0-5
Arafa
AF 410 840 (8.572) AM (Cen,4, 50.00, Ar,10000.0,554.26,1.00,LS 5)
QTof Premier HAB321

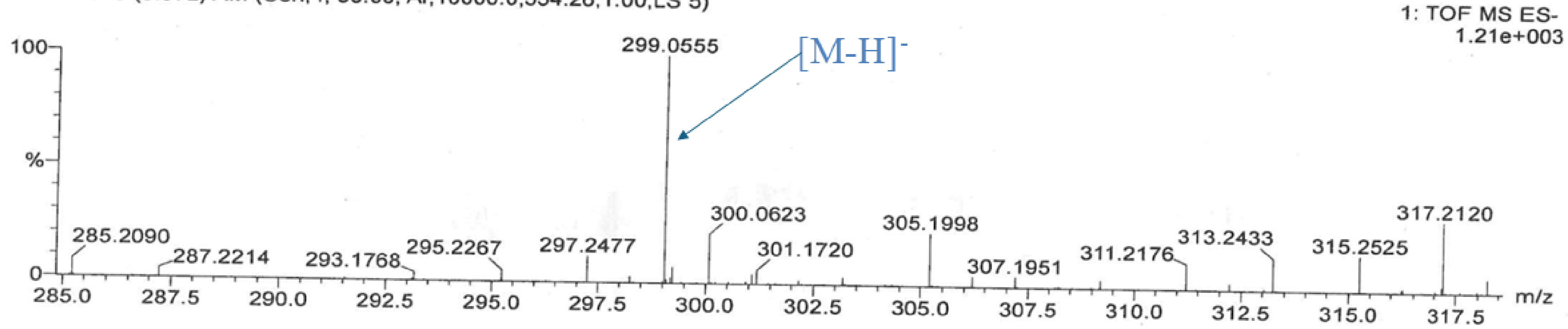

Fig. S35: HRESI-MS (-ve) of compound 4.

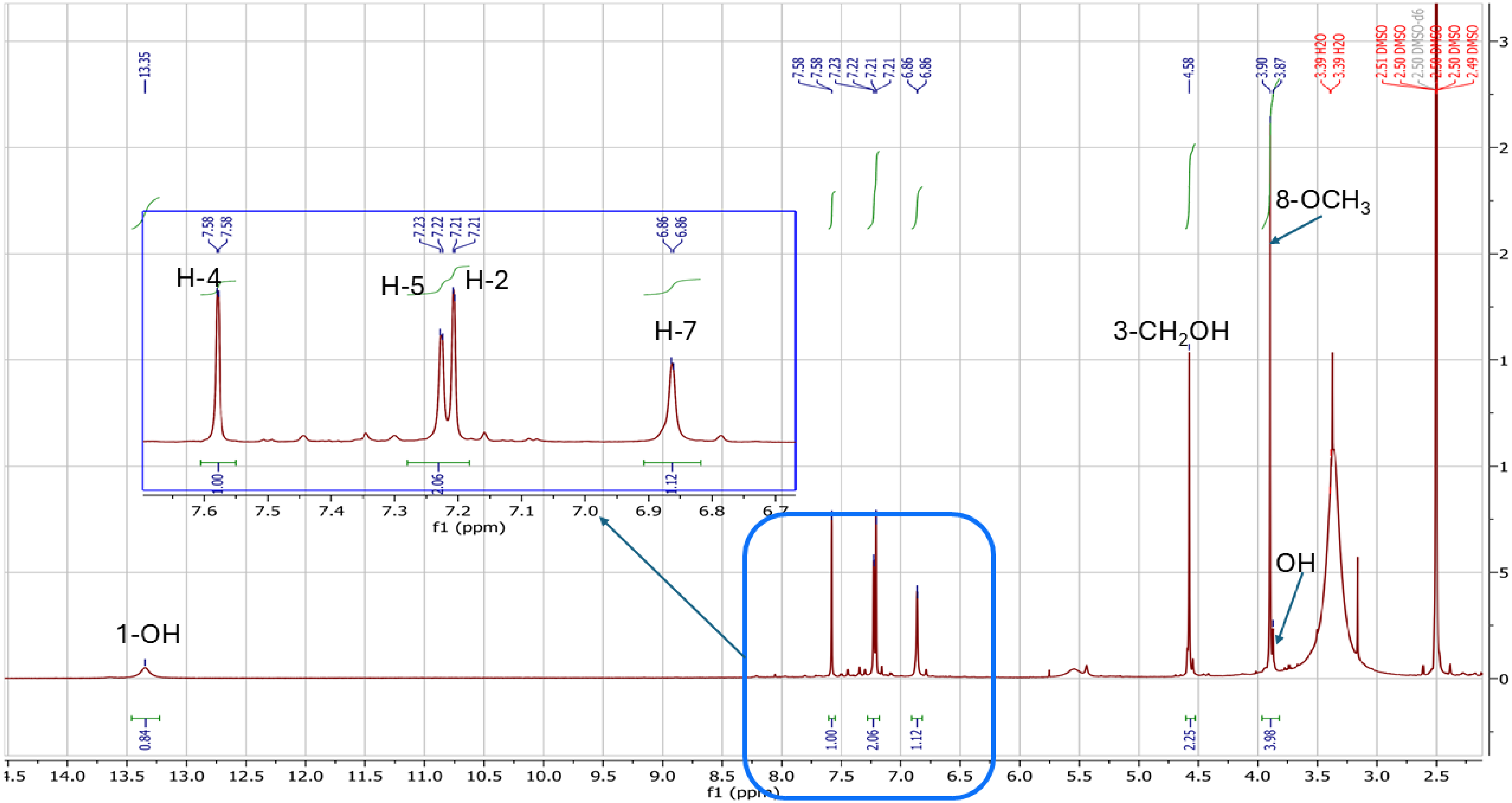

Fig. S36: ^{1}H NMR spectrum of compound 4 (DMSO-d_6).

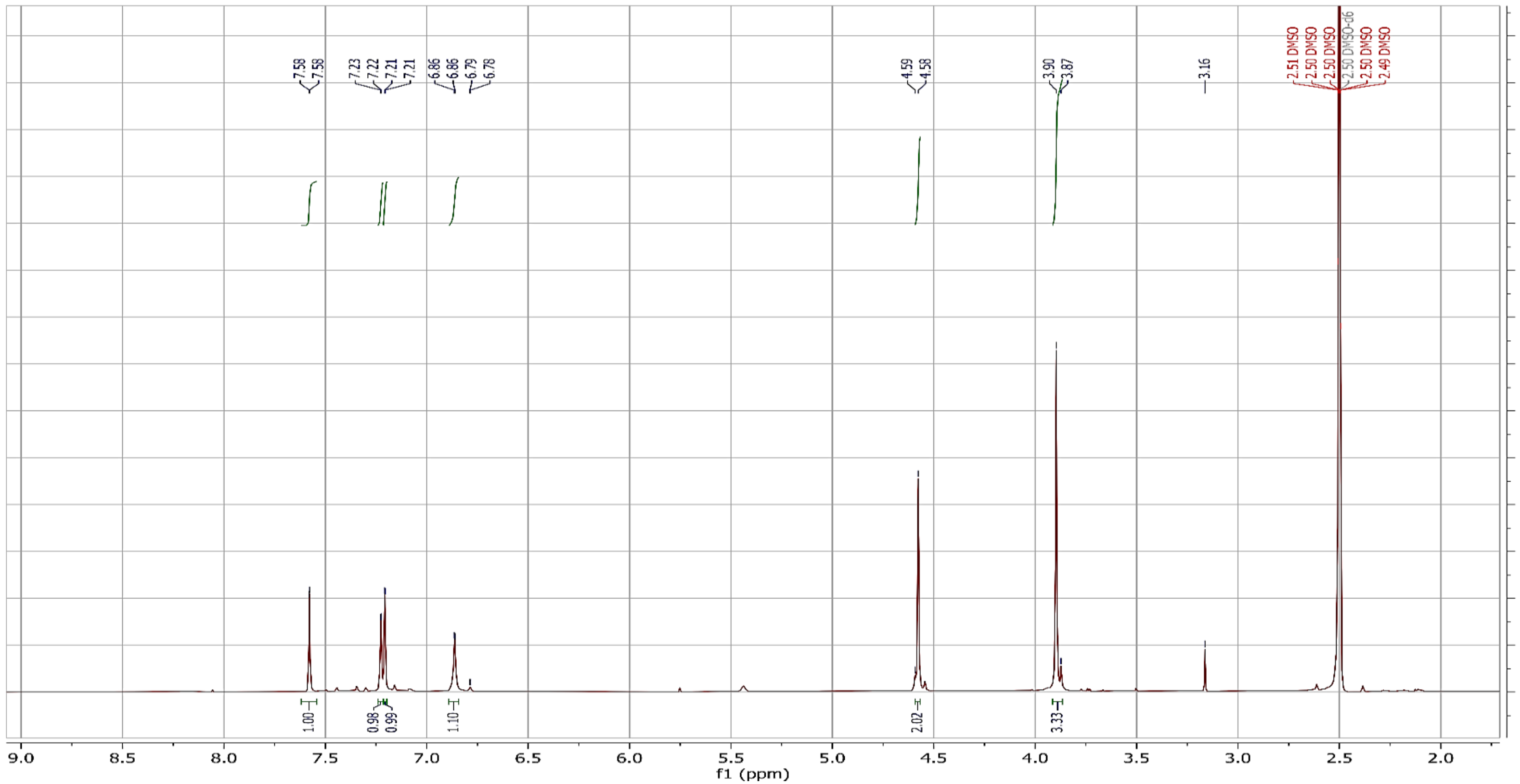

Fig. S37: ^{1}H NMR spectrum of compound 4 after water suppression.

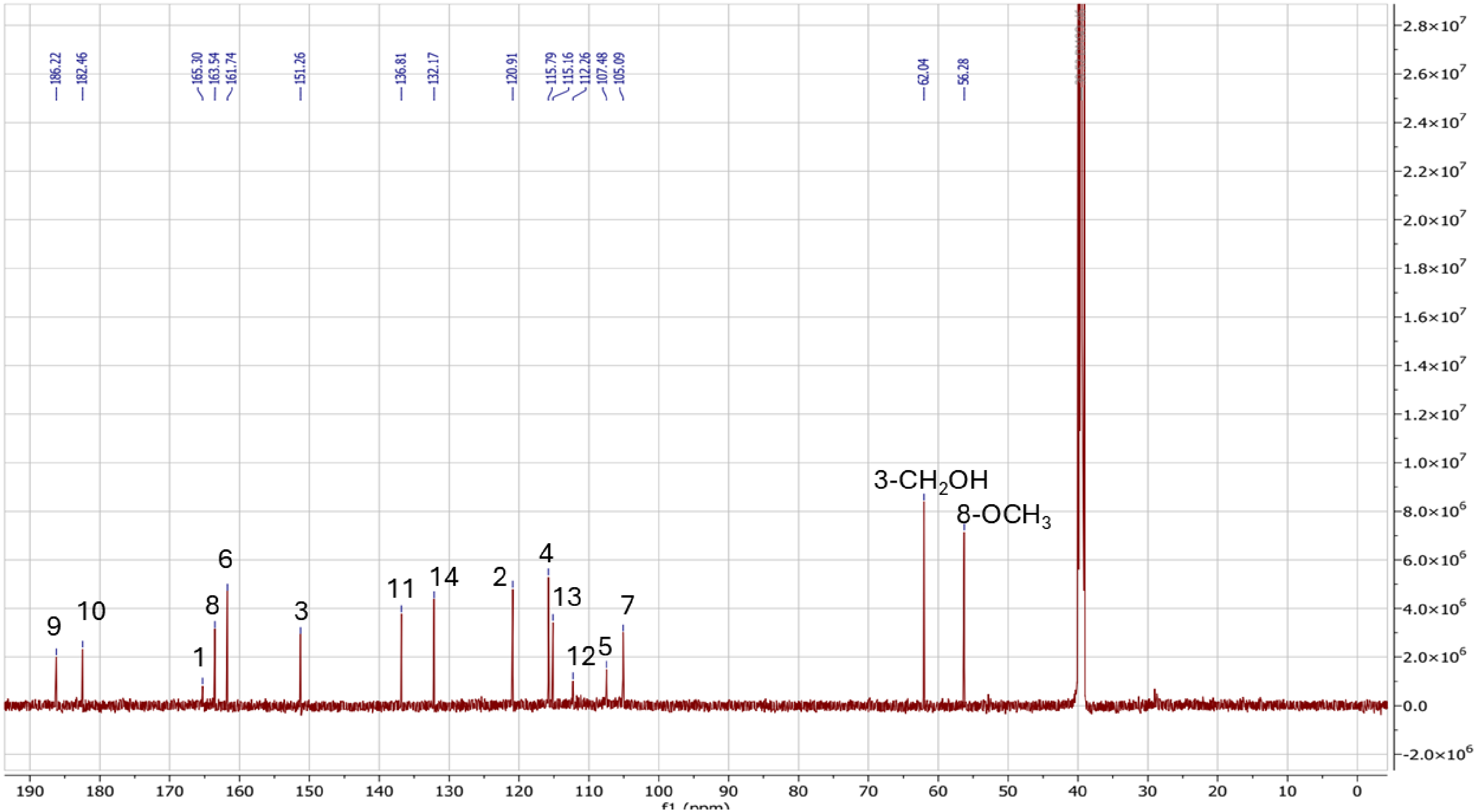

Fig. S38: ^{13}C NMR spectrum of compound 4 (DMSO-d_6).

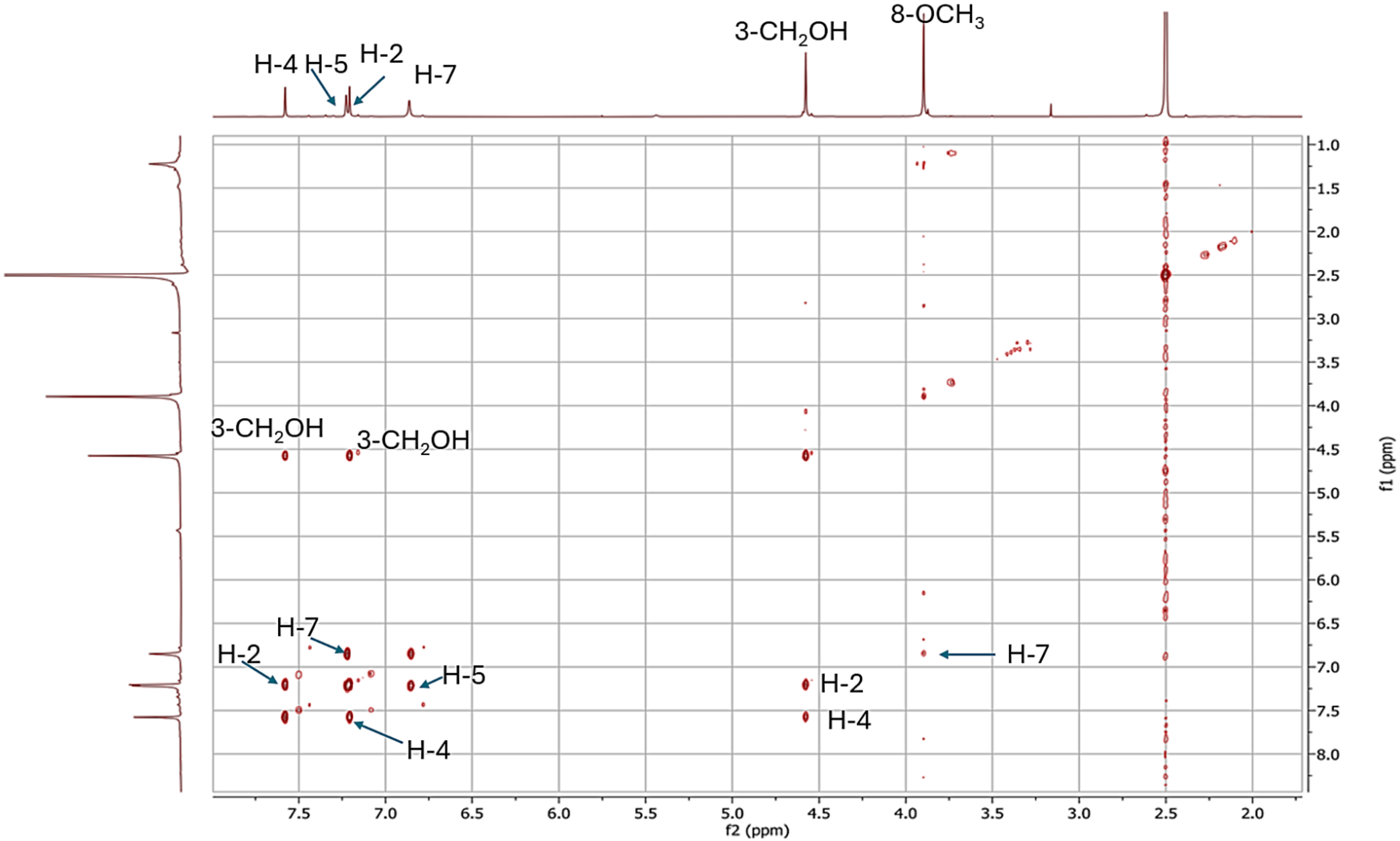

Fig. S39: COSY spectrum (DMSO-d_6, 600 MHz) of compounds 4.

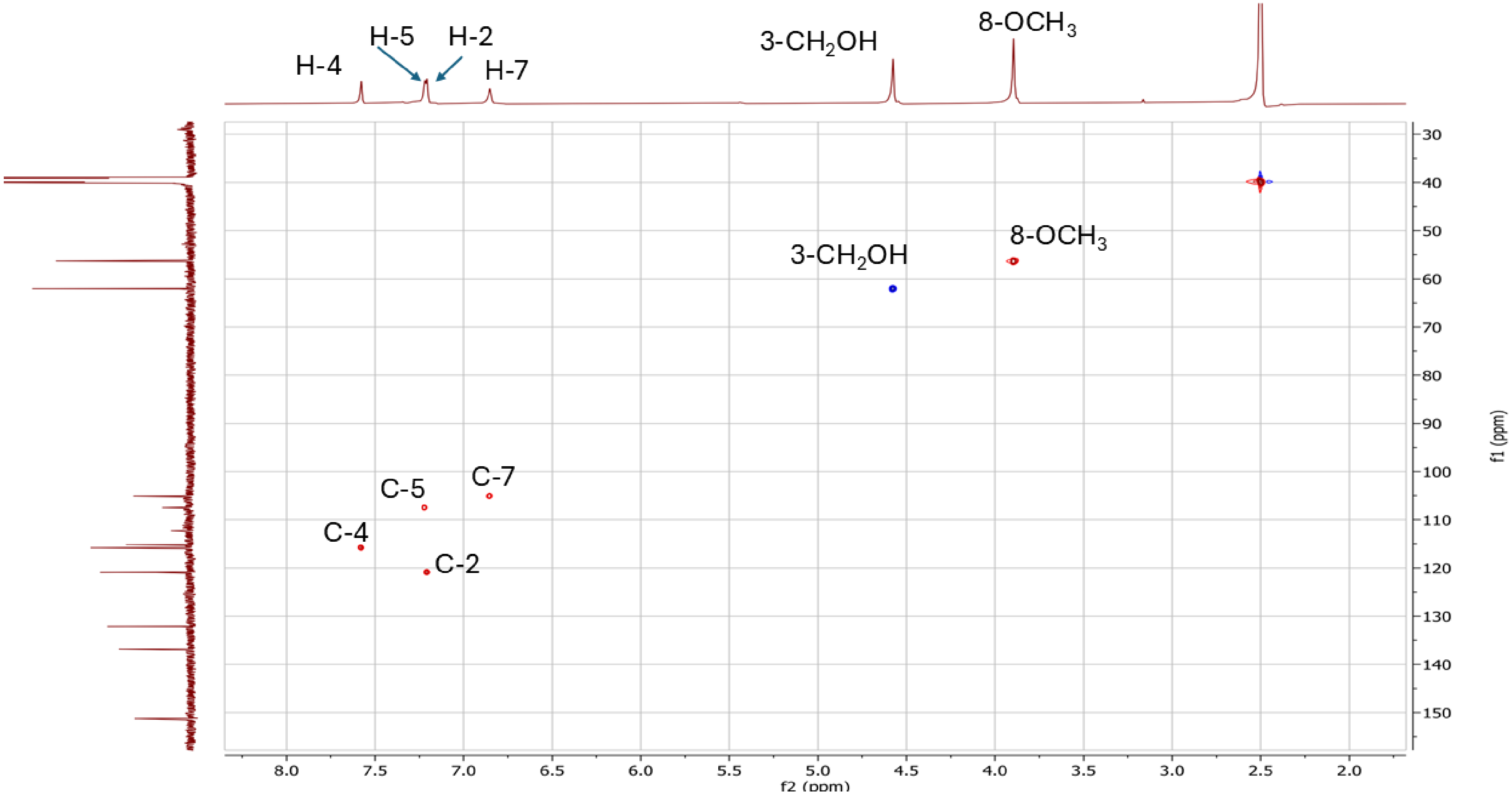

Fig. S40: HSQC spectrum (DMSO-d_6) of compound 4.

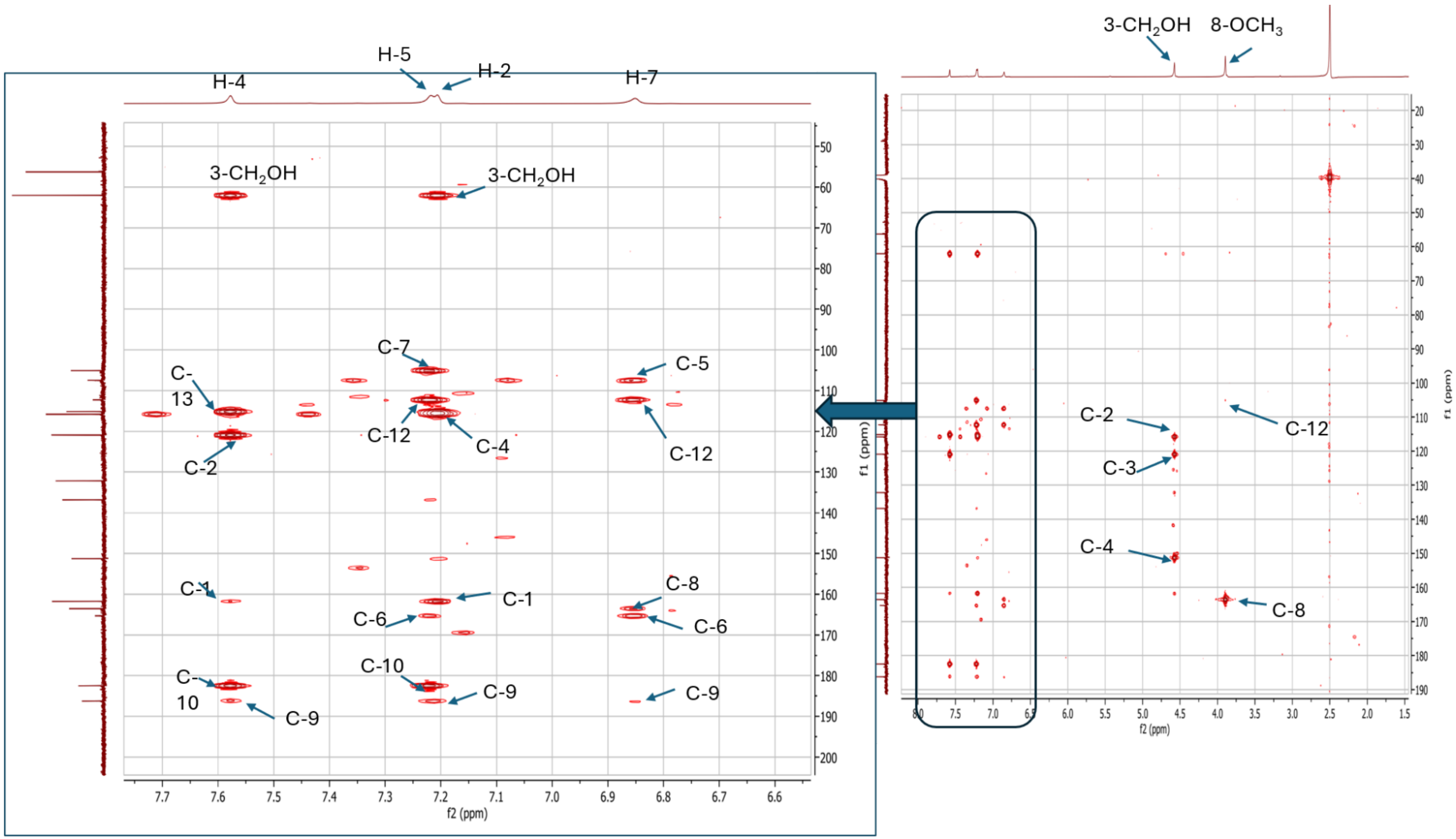

Fig. S41: HMBC spectrum (DMSO-d$_6$) of compound 4.

Compound 5

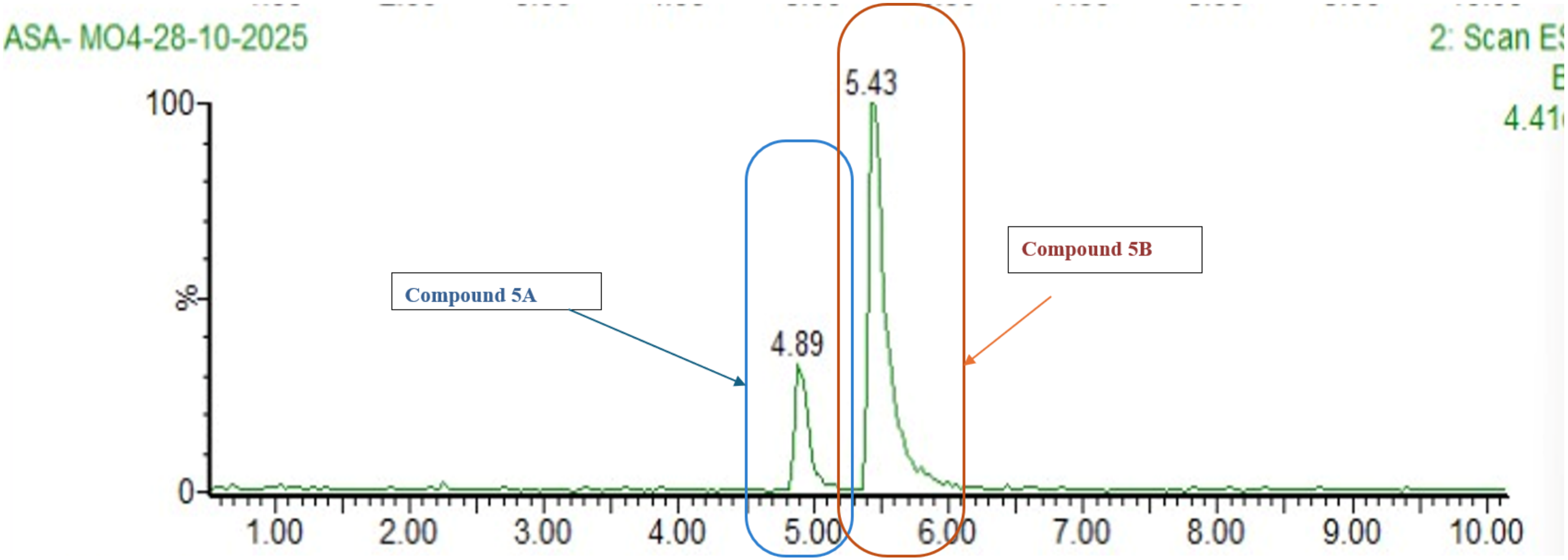

Fig. S42: Analytical LCMS (photodiode array) analysis of compound 5B in orange and 5A in blue.

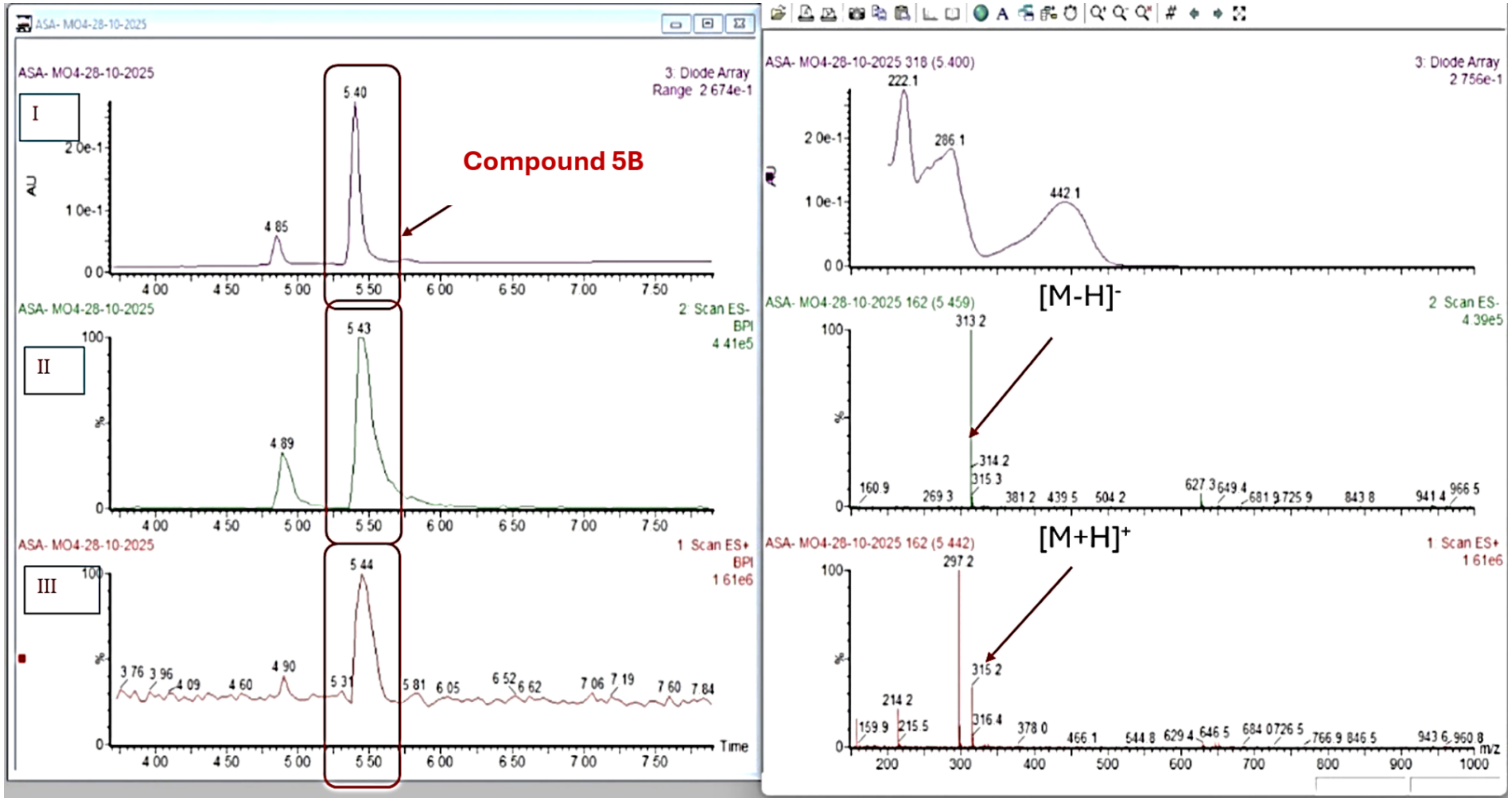

Fig. S43: LC-MS total ion chromatogram of compound 5B. (I) Diode array spectrum (left panel) and UV spectrum (right panel). (II) In negative ion mode (left panel) and mass spectrum (right panel). (III) In positive ion mode (left panel) and MS spectrum (right panel).

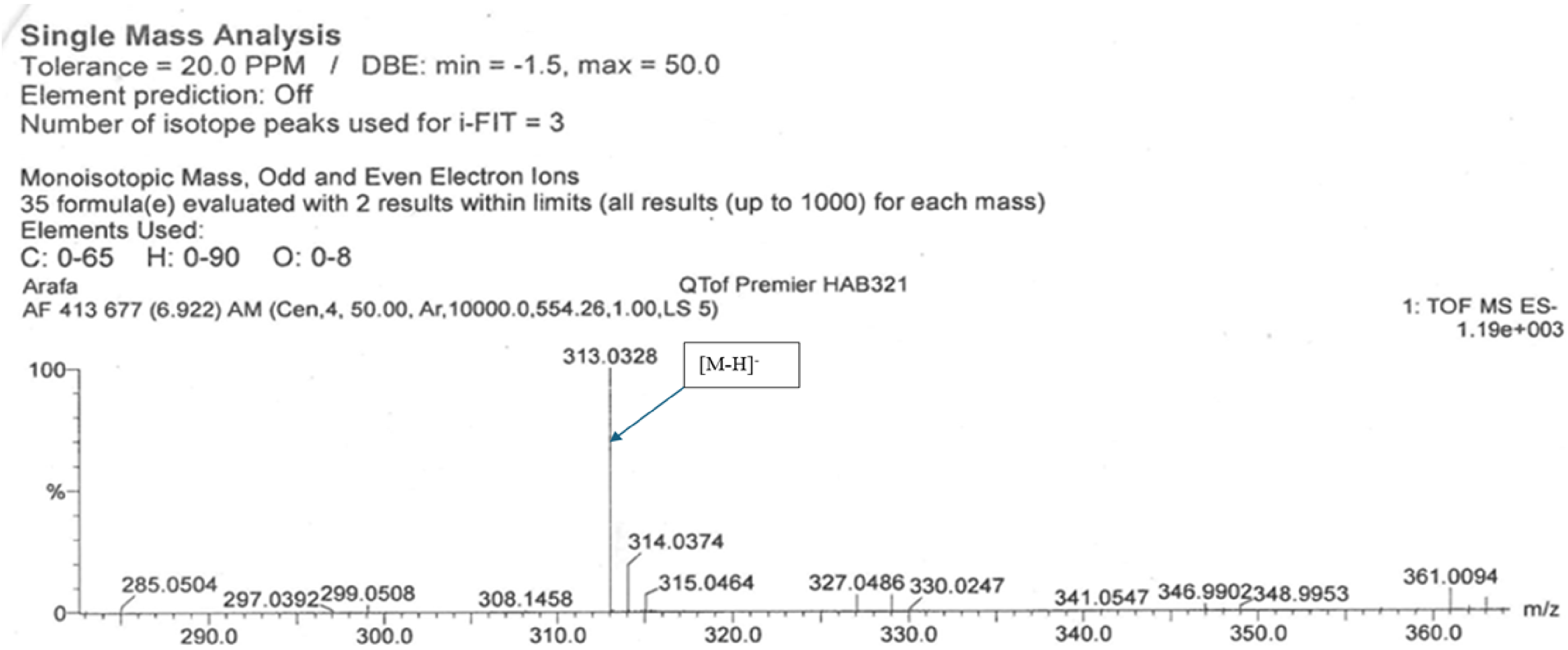

Fig. S44: HRESI-MS (-ve) of compound 5B.

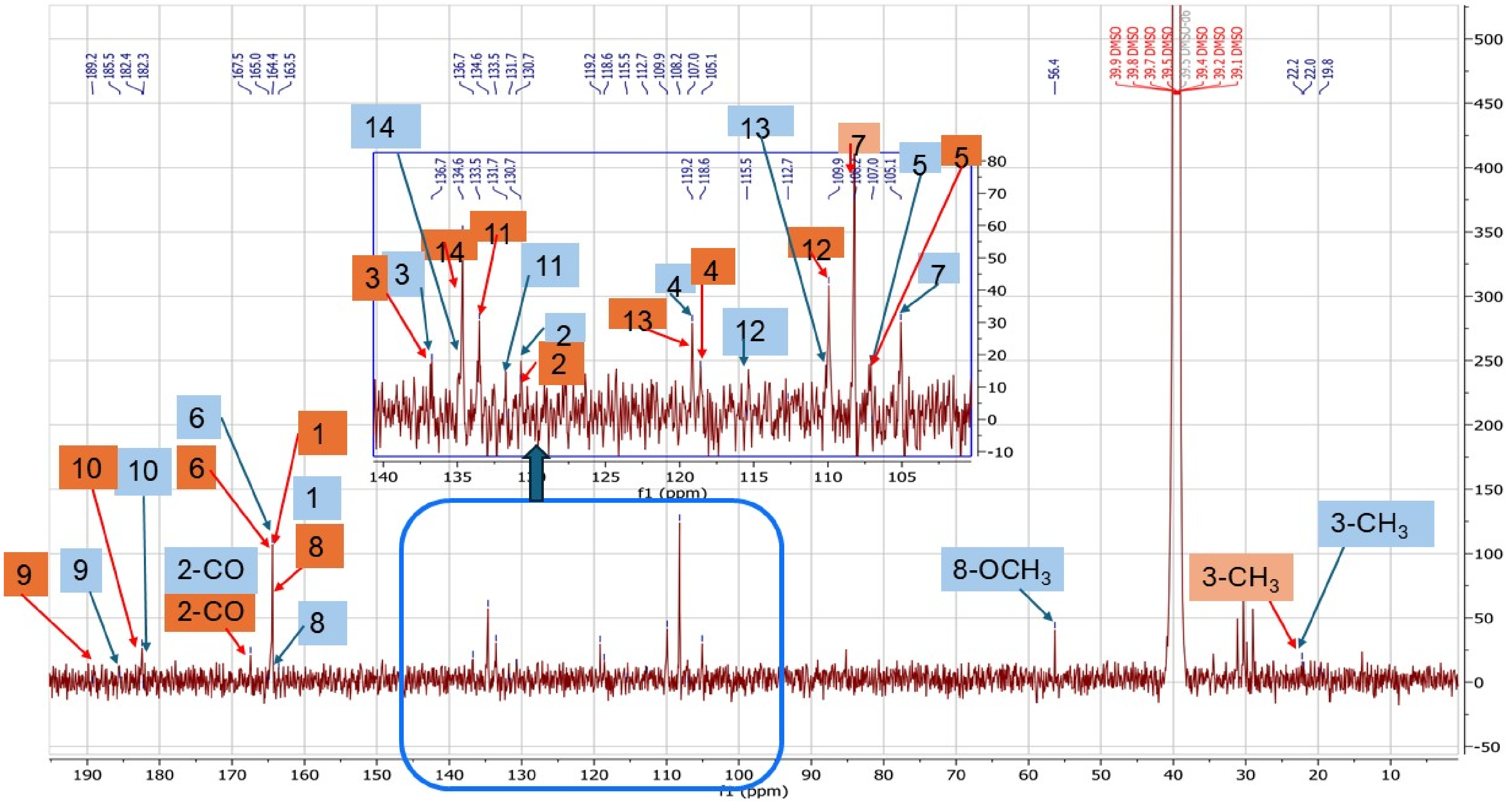

Fig. S45: ^{13}C NMR spectrum (DMSO-*d6*, 151 MHz) of compounds 5B (in orange color) and 5A (in blue color).

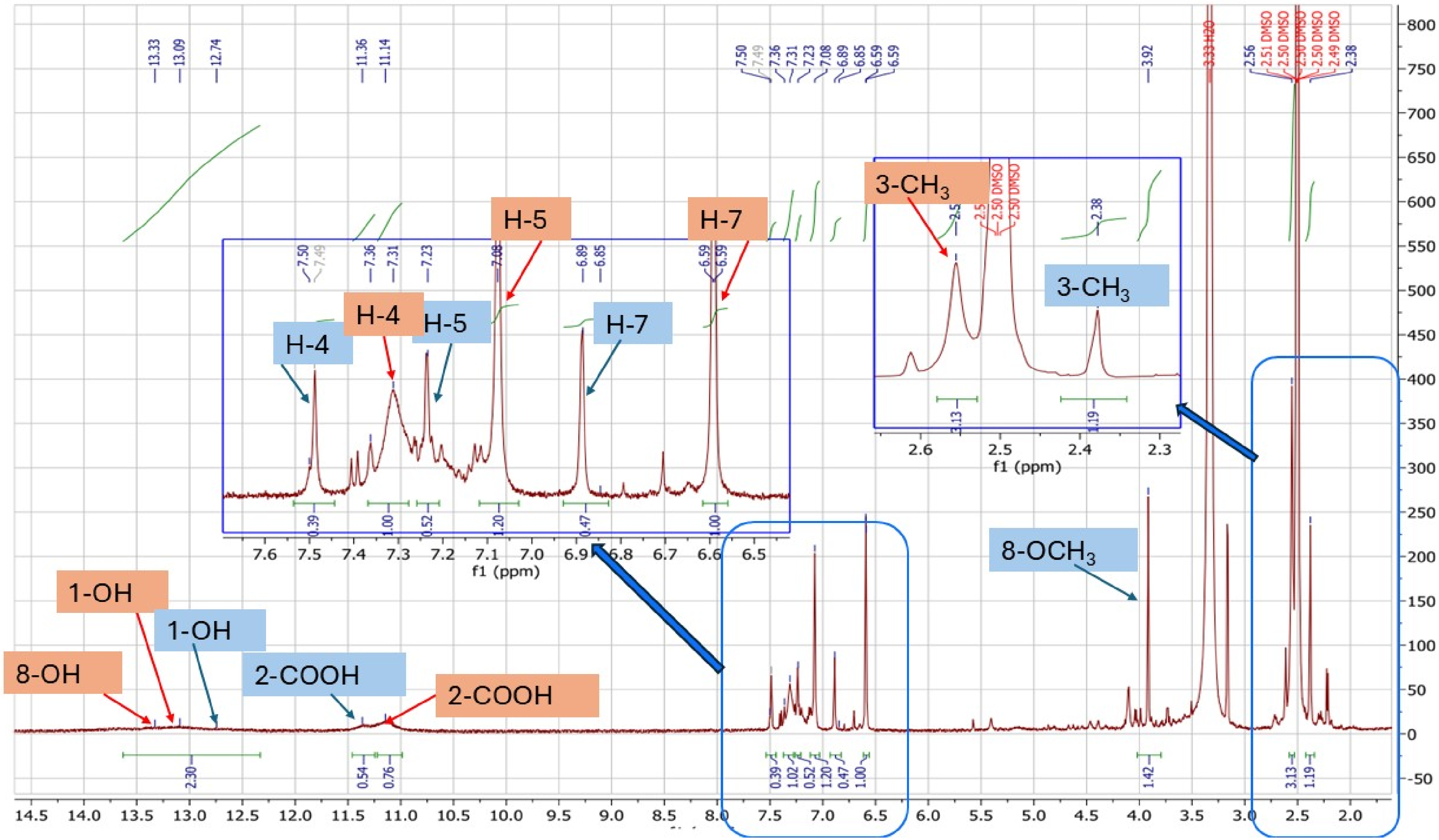

Fig. S46: ^{1}H NMR spectrum (DMSO-*d6*) of compound 5B (in orange color) and 5A (in blue color).

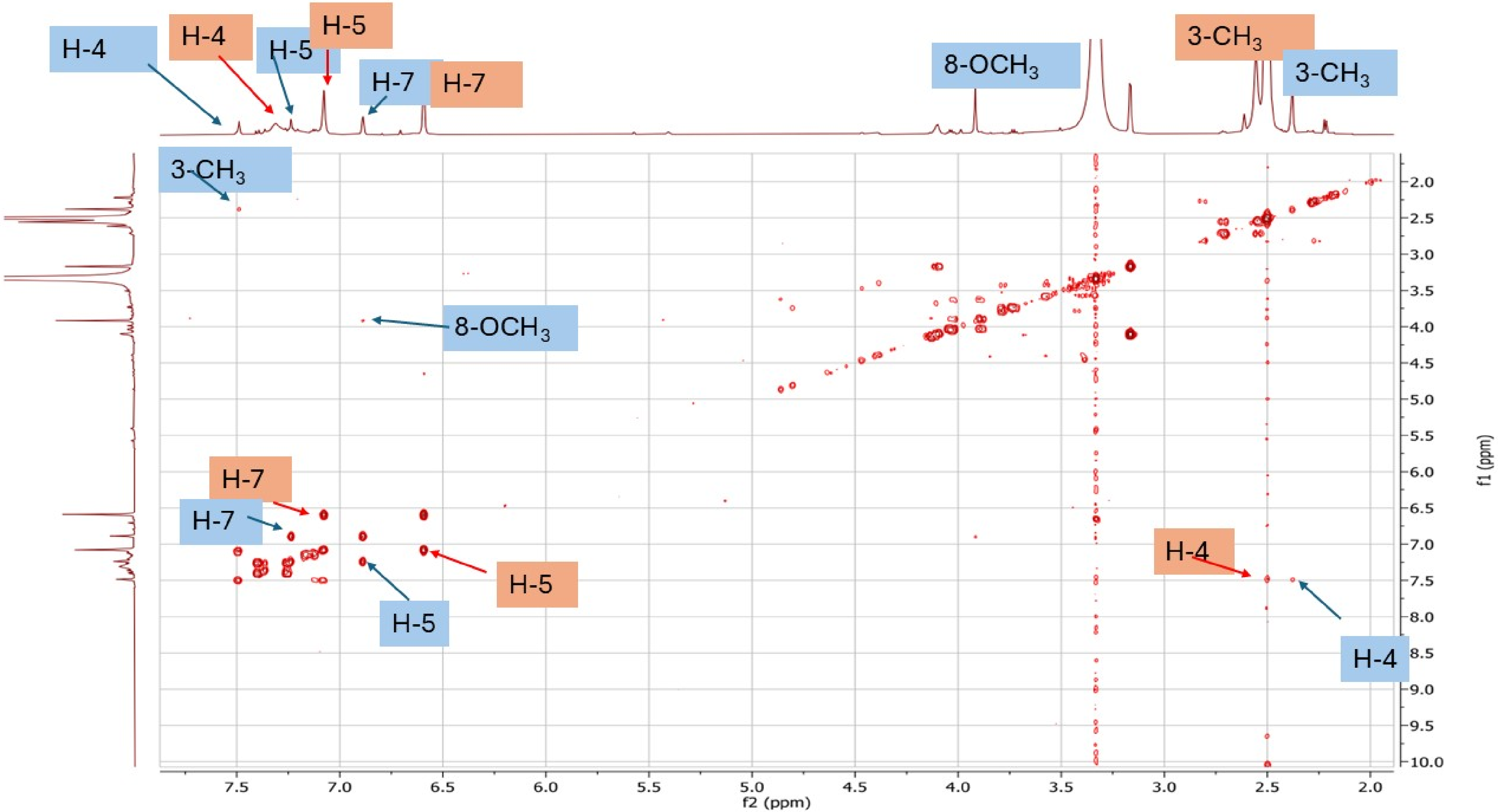

Fig. S47: COSY spectrum (DMSO-*d6*) of compound 5B (in orange color) and 5A (in blue color).

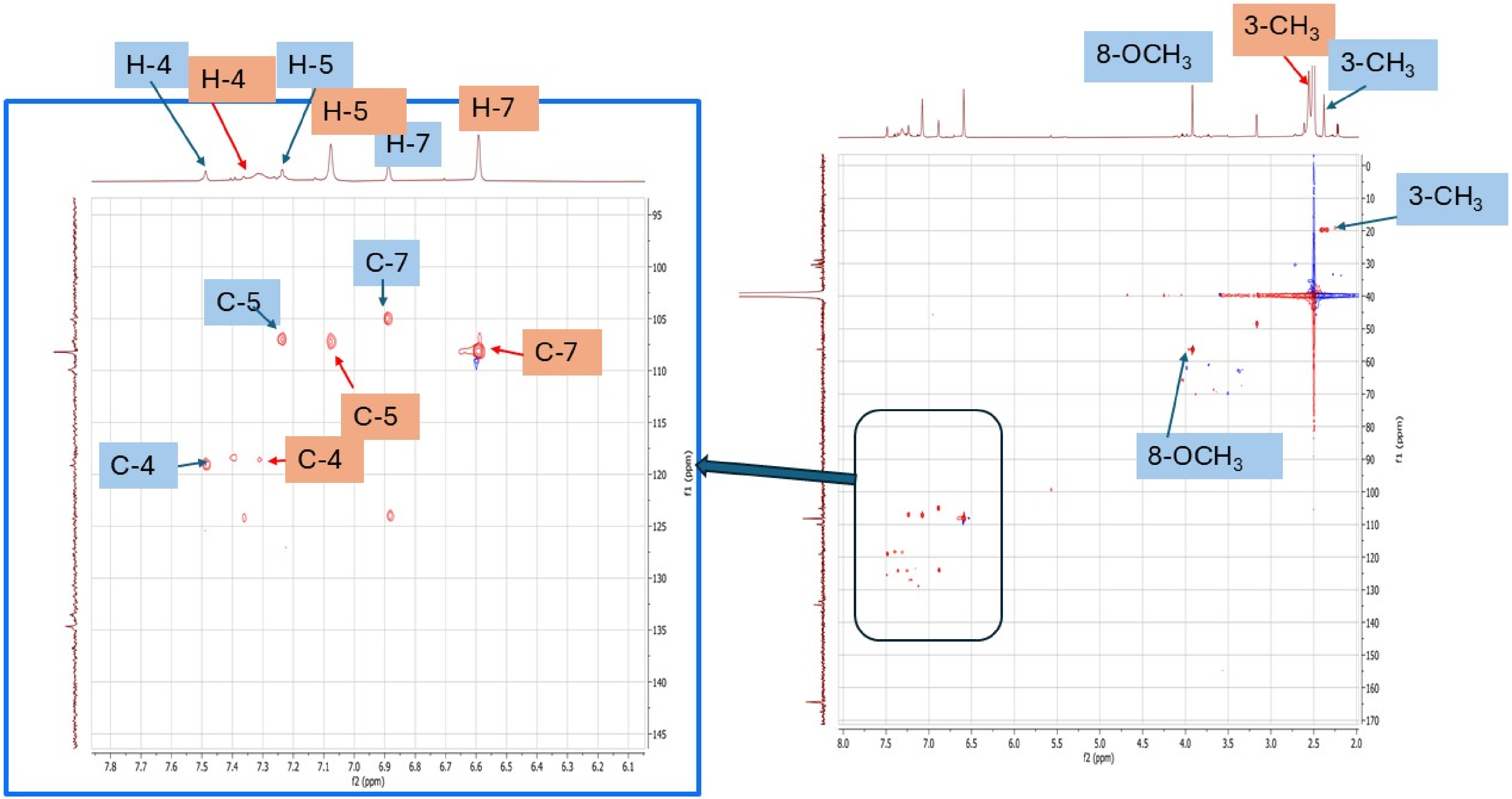

Fig. S48: HSQC spectrum (DMSO-d$_6$) of compound 5B (in orange color) and 5A (in blue color).

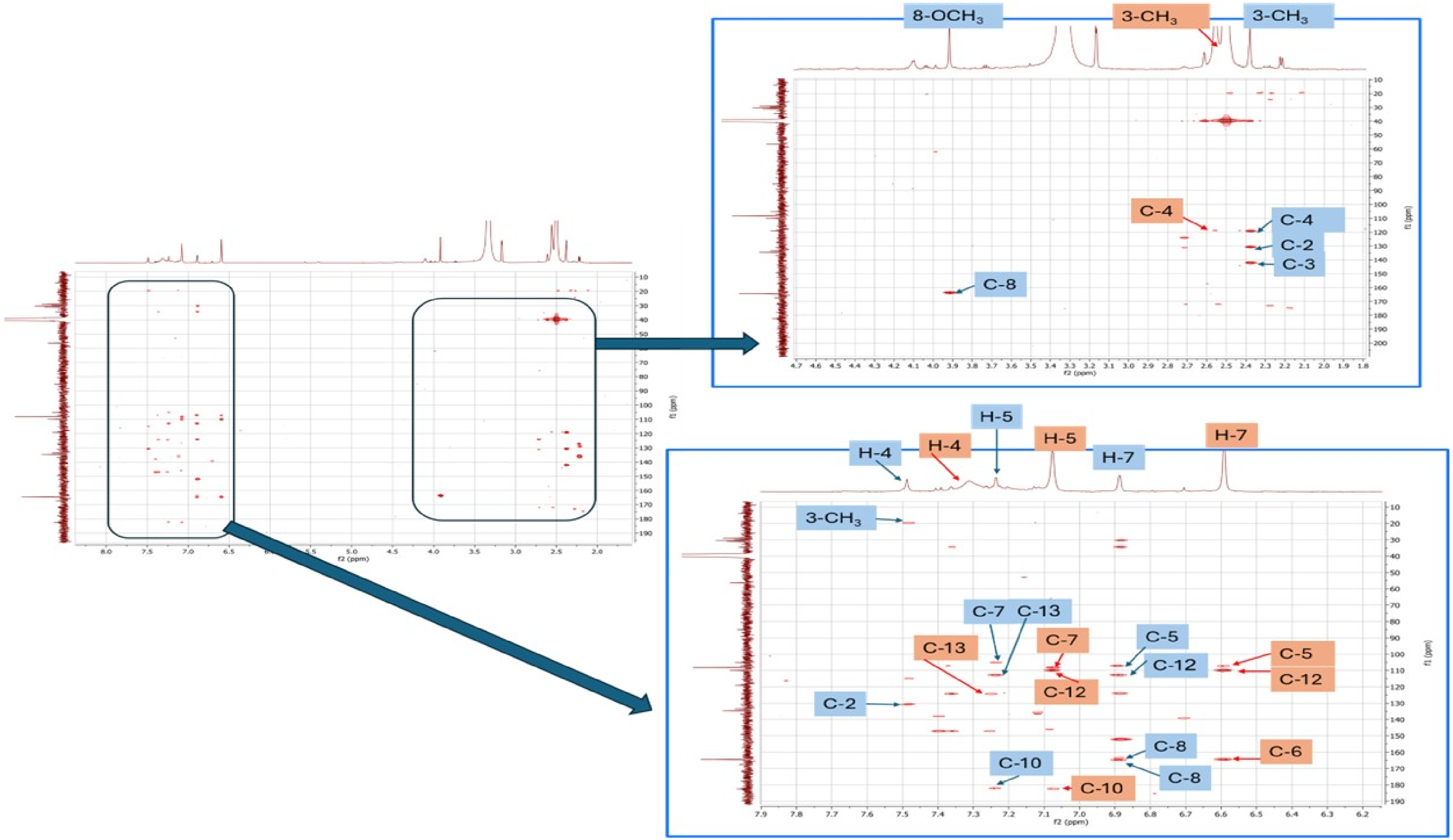

Fig. S49: HMBC spectrum (DMSO-d_6) of compound 5B (in orange color) and 5A (in blue color).

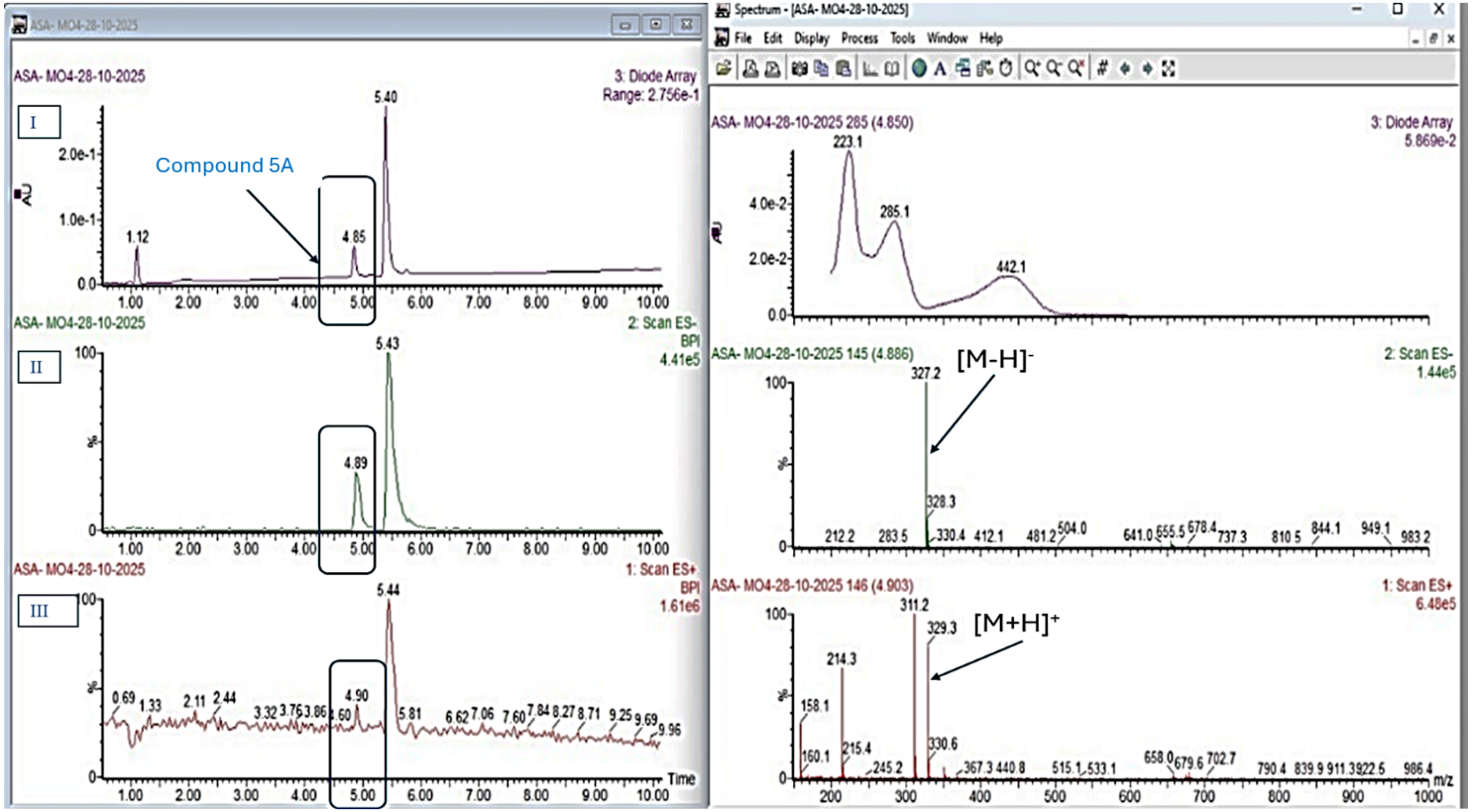

Fig. S50: LC-MS total ion chromatogram of compound 5A. (I) Diode array spectrum (left panel) and UV spectrum (right panel). (II) In negative ion mode (left panel) and mass spectrum (right panel). (III) In positive ion mode (left panel) and MS spectrum (right panel).

lemental Composition Report **Page 1**

Single Mass Analysis
Tolerance = 20.0 PPM / DBE: min = -1.5, max = 50.0
Element prediction: Off
Number of isotope peaks used for i-FIT = 3

Monoisotopic Mass, Even Electron Ions
27 formula(e) evaluated with 1 results within limits (all results (up to 1000) for each mass)
Elements Used:
C: 0-45 H: 0-65 O: 0-5

Arafa QTof Premier HAB321
AF 413B 608 (6.211) AM (Cen,4, 70.00, Ar,10000.0,554.26,1.00,LS 5) 1: TOF MS ES- 1.98e+003

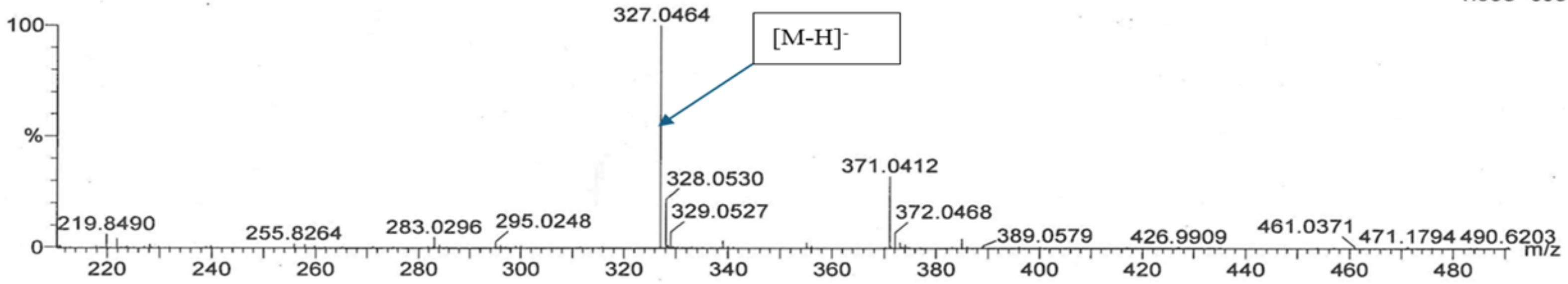

Fig. S51: HRESI-MS (-ve) of compound 5A.

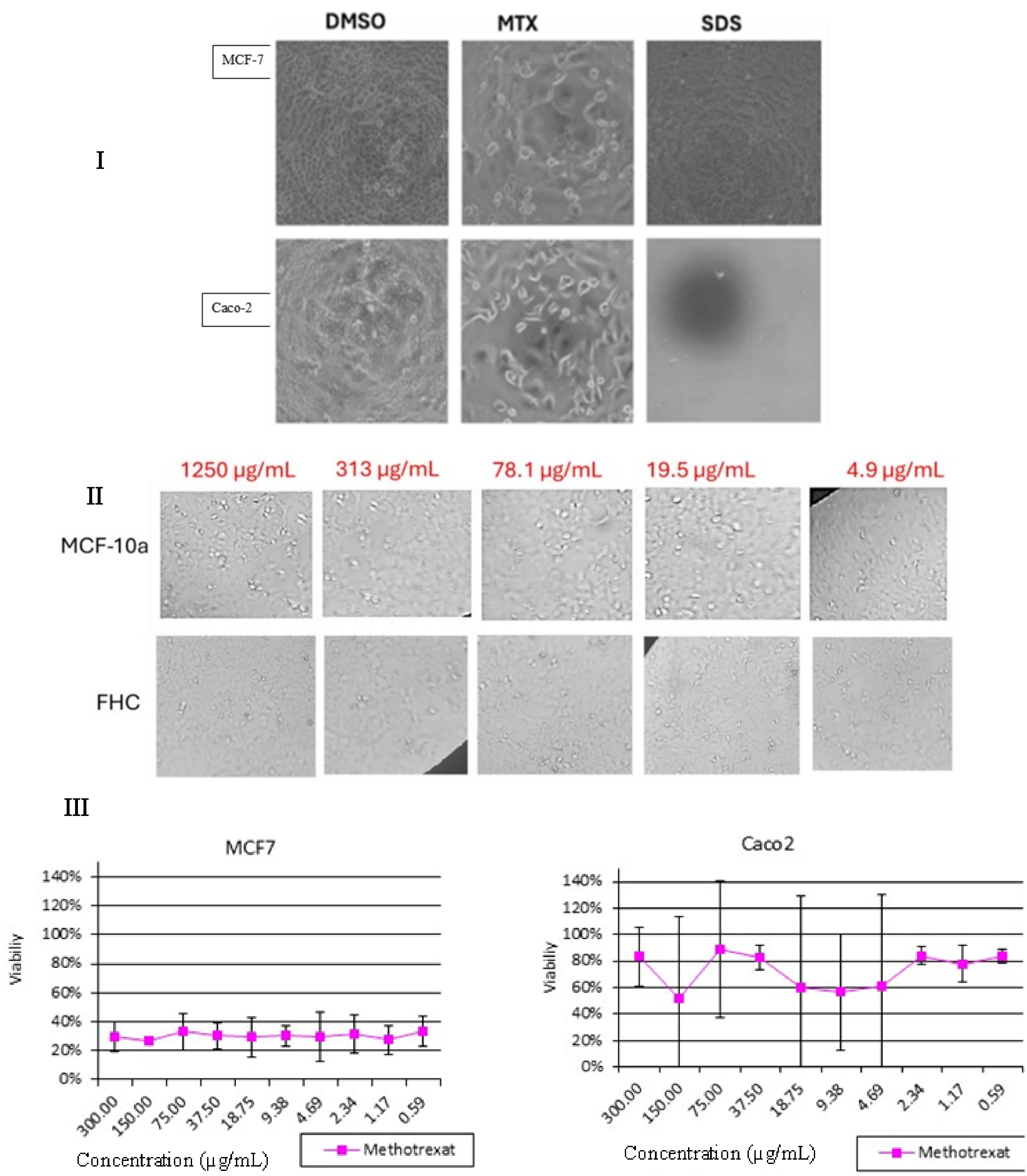

Fig. S52: (I) Effect of DMSO, methotrexate and SDS on breast MCF-7 and colon Caco-2 cancer cell lines and (II) Effect of Am-10 on normal breast MCF10a and normal colon FHC cell lines. (III): Methotrexate dose response curve.

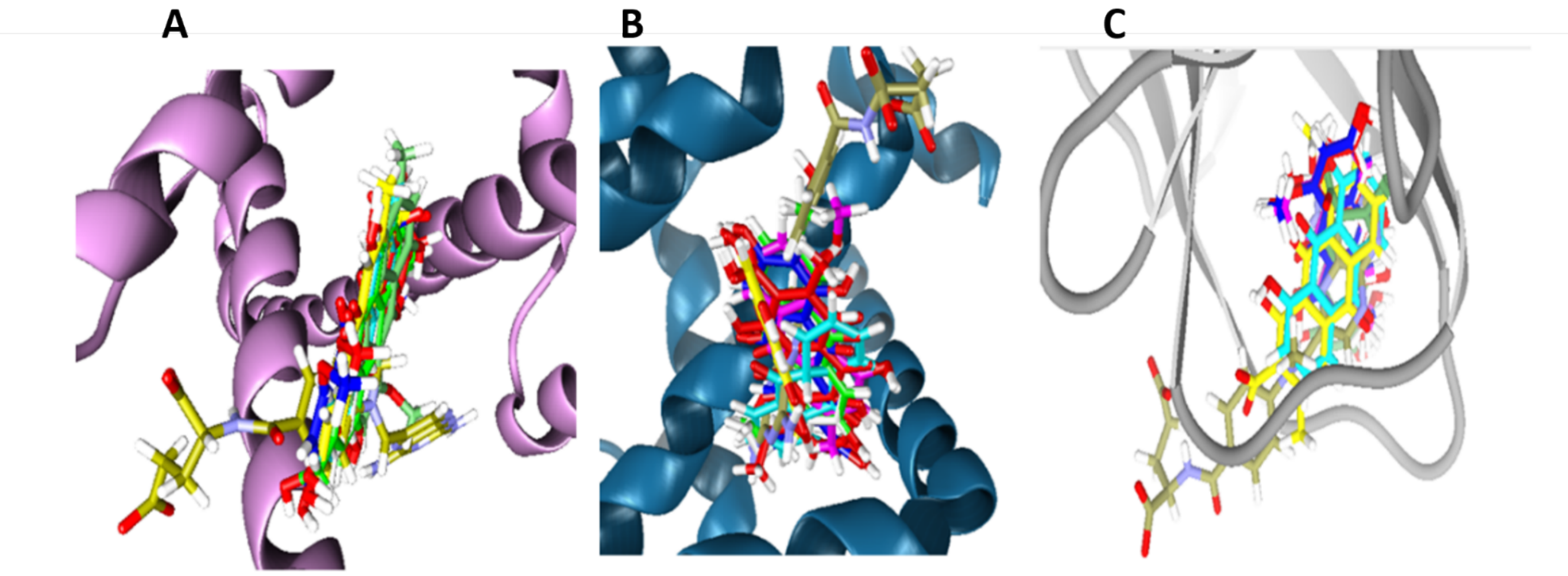

Fig. S53: Superimposable docked poses of methotrexate and metabolites **1-5** (all presented as sticks) to apoptosis genes (A): *Bax* (PDB ID:4BD6), (B): *Bcl-2* (PDB ID: 6O0K) and (C) *p53* (PDB ID: 8DC6). Methotrexate (olive green), compound **1** (white), compound **2** (sky blue), compound **3A** (red), compound **3B** (green), compound **4** (Light green) compound **5B** (Yellow) and compound 5A (blue).

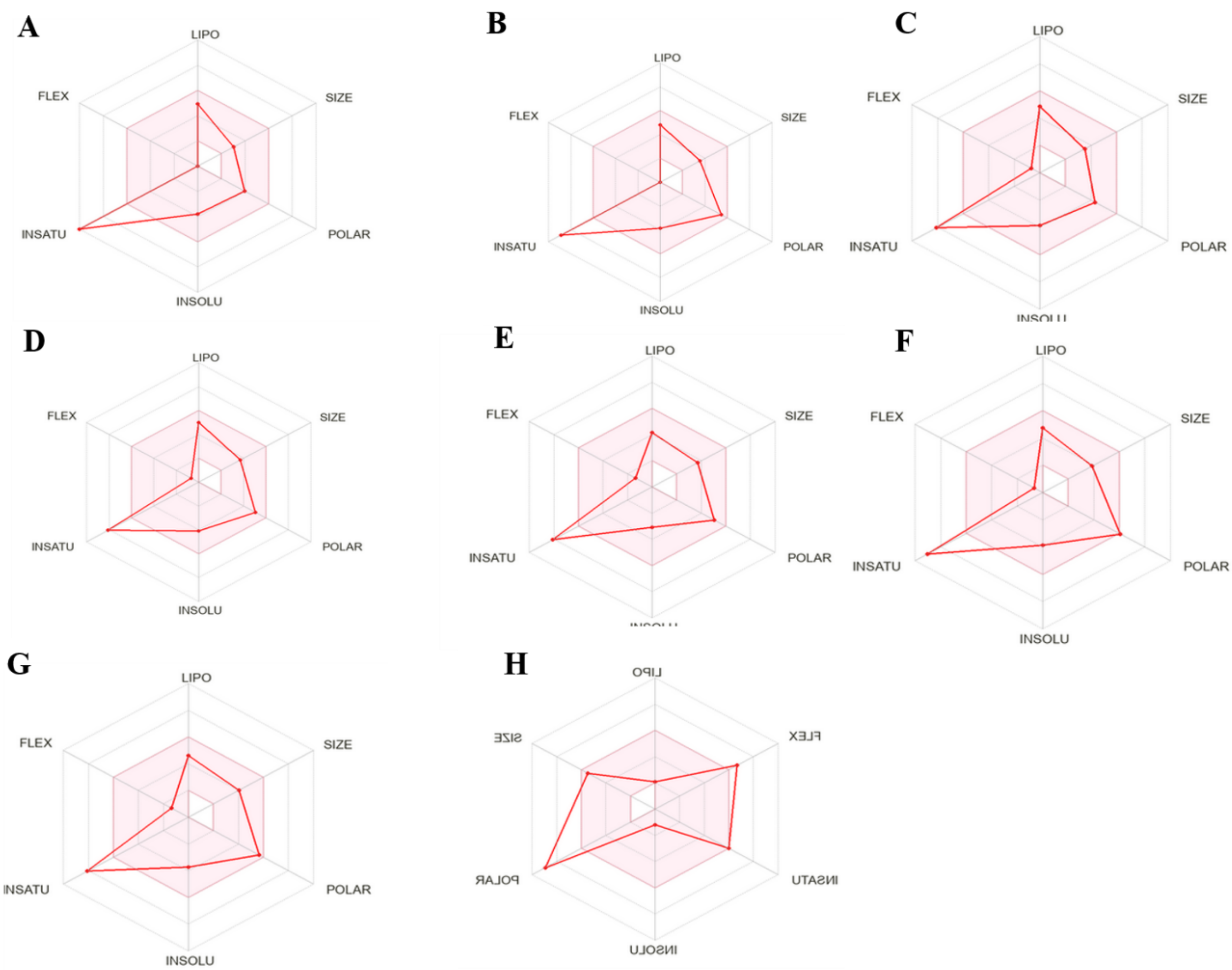

Fig. S54: Bioavailability radar of methotrexate and compounds **1-5** obtained from SWISSADME. The radar plot is based on six important physicochemical properties that influence oral bioavailability, namely lipophilicity (LIPO), molecular size (SIZE), polarity (POLAR), solubility (INSOLU), flexibility (FLEX) and saturation (INSATU). The pink-colored region in the radar represents the optimal physicochemical space associated with good oral bioavailability. Compound 1 (A), 2(B), 3A(C), 3B(D), 4(E), 5B(F), 5A(G), and methotrexate (H).

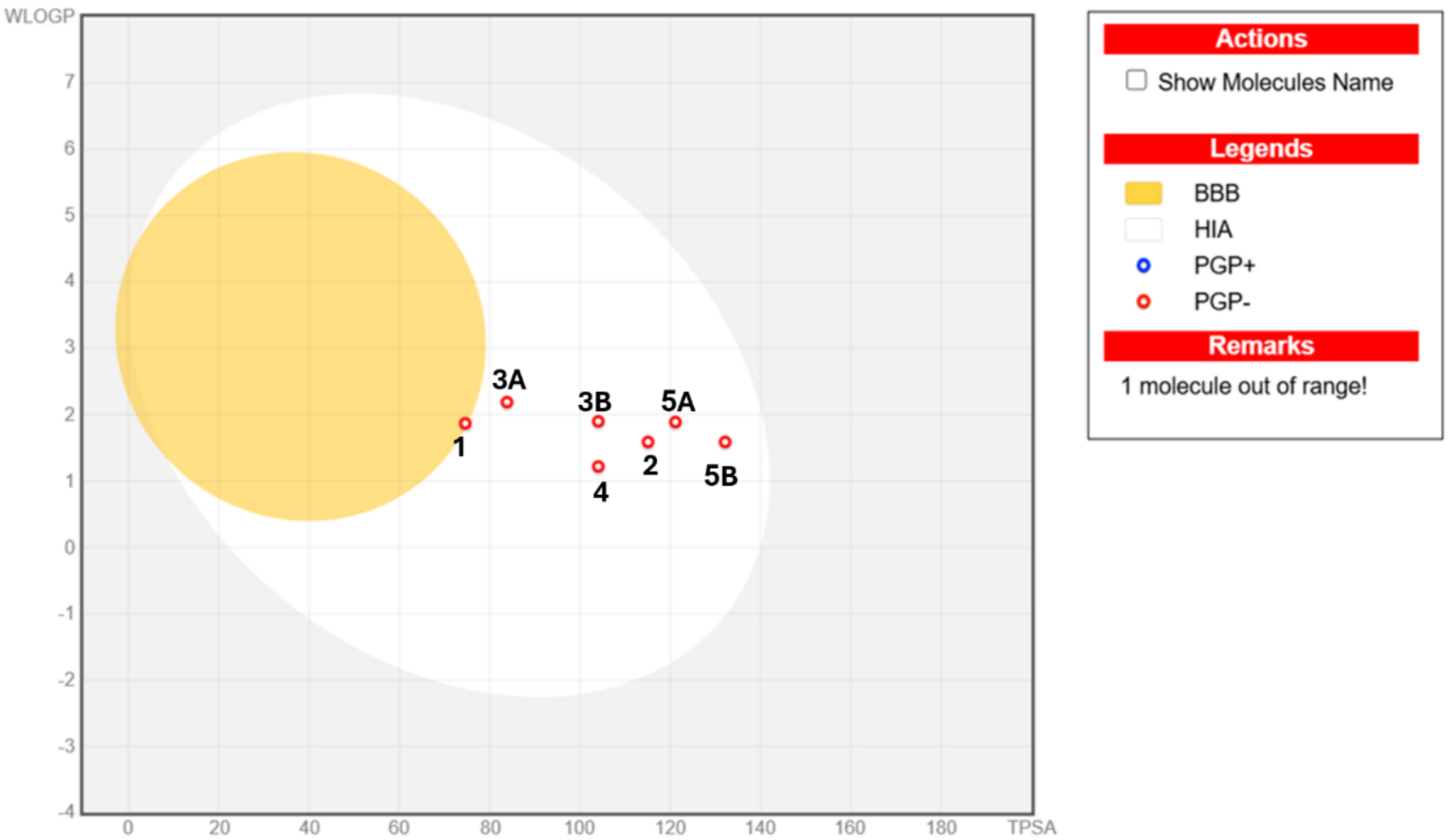

Fig. S55: Predicted Brain Or IntestinaL EstimateD permeation (BOILED-Egg) plot. BBB (blood brain barrier), HIA (human intestinal absorption), PGP- (not P-glycoprotein substrate).

Table S1: Evaluation of crude extract production by the isolated fungi using different fermentation media (rice, wheat, and potato dextrose broth).

Fungal Species	Rice (g/ 100g)	Wheat (g/ 100g)	Potato dextrose broth (g/400 mL)
	Crude extract yield (g) and color		
A. flavus	0.125 (Yellow)	0.030 (Yellow)	0.166 (Orange to yellow)
A. amstelodami (Am-10)	0.156 (Yellow)	0.180 (Orange to brown)	0.264 (Redish brown)
A. sydowii	0.072 (Brown)	0.095 (Dark brown)	0.021 Green

Table S2: R_f values and TLC detection results of isolated compounds isolated from *A. amstelodami* (Am-10).

		Compound				
		1	2	3 (A, B)	4	5 (A, B)
UV 265 nm		Dark brown	Dark brown	Dark brown	Dark brown	Dark brown
UV 365 nm		Yellow	Dark yellow	Orange	orange	Orange
$FeCl_3$		+ve Reddish-	+ve violet	+ve Violet	+ve violet	+ve redish-
10% Sulfuric acid		+ve Green	+ve orange	+ve Pink	+ve orange	+ve yellow
Anisaldehyde sulfuric		Orange	+ve orange	+ve Pink	+ve orange	+ve yellow
$AlCl_3$		Pink	Pink	Pink	Pink	Not clear
Dragendorff's		-ve	-ve	-ve	-ve	-ve
R_f	Pet. ether: EtOAc (8:2)	0.77	0.41	0.09	0.0	0.0
	CH_2Cl_2:EtOAc (7:3)	0.94	0.83	0.67	0.30	0.0
	CH_2Cl_2: MeOH (8:2)	0.96	0.96	0.96	0.92	0.20

Table S3: Gene primers for *Bax*, *Bcl-2* and *p53* apoptotic genes.

Gene	FORWARD PRIMER (5'→3')	REVERSE PRIMER (5'→3')
Bax	TGC TTC AGG GTT TCA TCC AG	GGC TGC CCA GTT TGC TTC A
Bcl-2	GGT GGG GTC ATG TGT GTG G	CGG TTC AGG TAC TCA GTC ATC C
p53	GTT CCG TCC CAA GCA ATG GAT G	TCT GAG TCG CCC TTC TGT CTT G
GAPDH	GAA GGT GAA GGT CGG AGT C	GAA GAT GGT GAT GGG ATT TC

Table S4: Tentatively annotated metabolites detected in *A. amstelodami* (Am-10) EtOAc extract by UPLC-HRMS/MS, including retention time, molecular formula, diagnostic fragment ions, and relative abundance.

Peak NO	RT	$[M-H]^-$	Error (ppm)	Name	Molecular formula	Fragmentation MS/MS	%Area	Ref.
**Sugars and sugar acids**								
8	0.84	179.0557	-1.01	Mannose	$C_6H_{12}O_6$	160.8418,141.8675,119.03	0.22	Data base
10	0.85	195.0509	-1.1	Gluconic acid	$C_6H_{12}O_7$	179.056, 161.045, 149.045	0.45	[13]
**Organic acids**								
11	1.29	89.02412	-2.8	Lactic acid	C₃H₆O₃	71.0140, 61.9880	0.75	[14]
12	1.49	128.035	2.52	Pidolic acid	$C_5H_7NO_3$	112.984, 100.9510	0.56	Data base
13	1.56	115.0033	-2.22	Fumaric acid	$C_4H_4O_4$	96.969, 71.0134	0.15	Data base
14	1.72	117.0190	-2.19	Succinic acid	$C_4H_6O_4$	98.9487, 73.0288	0.09	Data base
15	9.67	193.0502	-1.1	Ferulic acid	$C_{10}H_{10}O_4$	178.978, 134.8653	0.05	[15]
**Mycotoxins**								
16	8.4	153.0189	-1.34	Patulin	$C_7H_6O_4$	111.0082, 109.0290, 91.0033	0.04	[15]
17	11.73	327.051	0.09	Aflatoxin G1	$C_{17}H_{12}O_7$	309.0076, 283.0611, 240.0444	0.09	Data base
18	13.44	323.0553	0.8	Sterigmatocystin	$C_{18}H_{12}O_7$	297.0398	0.16	Data base
**IDP Alkaloids**								
19	12.78	322.15598	-1.32	Neoechinulin A	$C_{19}H_{21}N_3O_2$	285.0399, 249.1126	2.96	Database
**Benzaldehyde derivatives**								
20	18.26	297.14918	-0.37	Auroglaucin	$C_{19}H_{22}O_3$	281.1579, 279.1387, 277.1690, 269.0812	1.16	Database
21	18.43	331.15446	0.28	2-(3,6 dihydroxyhepta1,4-dien-1yl-3,6dihydroxy5-dimethyl allyl benzaldehyde	$C_{19}H_{24}O_5$	313.0737, 303.1637	0.02	Database
22	18.77	299.16509	-1.24	Dihydroauroglaucin	$C_{19}H_{24}O_3$	281.1536, 271.1694, 244.1697	3.5	Data base

**23**	19.19	301.18084	-1.55	Tetrahydroauroglaucin	$C_{19}H_{26}O_3$	283.1701, 273.1857, 258.1491, 248.96, 219.98	1.91	Data base
**24**	19.46	303.19591	0.37	Flavoglaucin	$C_{19}H_{28}O_3$	302.1895, 285.0395, 275.1975, 191.0072	7.2	Data base
**Lactones**								
**25**	16.6	331.11817	-0.04	Epiheveadride	$C_{18}H_{20}O_6$	287.1283, 259.1326	0.84	Data base
**26**	18.46	319.19073	0.63	Oryzalide B	$C_{19}H_{28}O_4$	301.0666	0.09	Data base
**Dihydro-isocoumarin polyketide**								
**28**	18.12	345.17008	0.34	Eurotiumide D	$C_{20}H_{26}O_5$	299.1653, 271.1699, 249.0588	0.25	Data base
**29**	19.19	347.18566	0.55	Eurotiumide A	$C_{20}H_{28}O_5$	301.189, 273.1847	0.08	Data base
**30**	19.3	349.20144	0.18	Dihydroeurotiumide A	$C_{20}H_{30}O_5$	303.1965, 275.2005	1.28	Data base
**Anthraquinones**								
**Anthraquinone monomers**								
**31**	10.24	317.06615	-0.08	Atrochrysone-2-carboxylate	$C_{16}H_{14}O_7$	273.0764, 187.0971	0.04	Data base
**32**	10.45	273.07626	0.13	Atrochrysone	$C_{15}H_{14}O_5$	149.0233, 112.9852, 244.9842	0.34	Data base
**33**	11.35	287.0919	0.17	Asperflavin	$C_{16}H_{16}O_5$	243.1231, 189.0551, 112.9851	0.23	Data base
**4**	11.68	299.05572	-0.52	Questinol	$C_{16}H_{12}O_6$	284.0665, 281.0643, 271.0565, 256.0369, 255.0650, 178.977, 91.003	3.8	Data base
**5A**	11.74	327.05074	-0.79	Dermolutein	$C_{17}H_{12}O_7$	309.0075, 312.0532, 283.0610, 299.0550, 268.0029, 240.0444,	2.38	5
**5B**	12.38	313.03485	-0.05	Endocrocin	$C_{16}H_{10}O_7$	298.0562, 295.0338, 285.9382, 269.0449	3.45	
**34**	12.76	285.04018	-0.93	Citreorosein	$C_{15}H_{10}O_6$	270.03456, 267.0876, 257,0875, 268.0990, 240.532,178.997, 112.985	1.9	
**35**	12.88	371.11298	0.28	Averantin	$C_{20}H_{20}O_7$	311.0919, 296.1415, 269.0835, 329.232	0.2	Data base
**40**	13.8	339.0504	0.24	Versicolorin C	$C_{18}H_{12}O_8$	321.0715, 311.0554	0.03	Data base
**36**	13.21	301.0346	0.76	Paeciloquinone C	$C_{15}H_{10}O_7$	245.0814	0.22	Data base
**37**	13.23	401.0868	1.15	Variecolorquinone A	$C_{20}H_{18}O_9$	383.0577, 373.088, 355.0822, 311.0551	0.04	5
**38**	13.25	297.0397	0.7	Rhein methylester	$C_{16}H_{10}O_6$	282.0618, 256.0547	0.66	Data base
**39**	13.59	341.06618	-0.15	Acetyl questinol	$C_{18}H_{14}O_7$	323.0544, 299.0550	0.94	Data base
**41**	14.07	357.0609	0.39	Asparasone A	$C_{18}H_{14}O_8$	339.0517, 311.0561, 283.0606	0.12	

**3A**	14.12	283.06012	1.87	Questin	$C_{16}H_{12}O_5$	255.0632, 265.0553, 268.0593, 240.0424, 91.003	2.15	[5]
**42**	14.2	283.0241	0.35	Rhein	$C_{15}H_8O_6$	212.1072, 112.9852	0.05	[16]
**43**	14.32	299.0559	-1.19	Erythroglaucin	$C_{16}H_{12}O_6$	255.0678, 284.0321, 178.977, 91.003	0.52	[5]
**3B**	14.61	299.05581	-0.84	Rubrocristin	$C_{16}H_{12}O_6$	284.0515, 281.0553, 271.0333, 255.0893,	2.48	
**44**	15.37	283.06051	0.49	Physcion	$C_{16}H_{12}O_5$	268.0355, 265.0602, 255.0876, 240.0425	0.29	
**27**	15.4	385.0918	1.4	Versicolorone	$C_{20}H_{18}O_8$	283.0603, 367.0470, 194.0816	0.05	Data base
**45**	15.5	269.04518	-0.66	Emodin	$C_{15}H_{10}O_5$	251.0699, 241.0822, 225.0180, 254.0557, 195.0674, 194.0817	1.24	[16]
**46**	15.89	257.0813	0.31	Dihydroemodin anthrone	$C_{15}H_{14}O_4$	229.0852, 213.0415, 112.9853	0.28	Data base
**2**	16.14	285.0403	-1.37	Catenarin	$C_{15}H_{10}O_6$	257.0136, 270.042, 267.0253 211.028108.0064	3.25	[5]
**1**	16.8	239.03451	2.32	Danthron	$C_{14}H_8O_4$	211.0254, 221.06658, 194.0817	2.6	Data base
**47**	16.99	269.08145	-0.27	Physcion anthrone B	$C_{16}H_{14}O_4$	251.0543, 112.9853	0.11	Data base
**48**	18.21	255.06581	-0.32	Emodin anthrone	$C_{15}H_{12}O_4$	237.0409, 227.0469, 211.0254	0.2	Data base
**Anthraquinone glycosides and dimers**								
**49**	10.59	445.0766	0.9	Rhein-O-glucoside	$C_{21}H_{18}O_{11}$	283.0094, 239.0548, 178.9774	0.1	[16]
**50**	13.73	537.0816	1.06	Sennidin A	$C_{30}H_{18}O_{10}$	493.0132, 283.0607, 269.0818	0.03	
**51**	15.71	523.1387	1.13	Emodin-physcion bianthrone	$C_{31}H_{24}O_8$	269.0814, 254.0583	0.04	Data base
**52**	16.84	509.123	1.26	Emodin bianthrone	$C_{30}H_{22}O_8$	269.0814, 255.0534, 241.0822, 225.0180	0.09	Data base
**Miscellaneous**								
**53**	13.88	407.0224	3.43	Variecoxanthone B	$C_{25}H_{28}O_5$	389.0648, 361.0701, 345.007839	0.07	Data base
**54**	15.04	415.1756	1.38	Xanthomonasin B	$C_{23}H_{28}O_7$	371.1785, 369.0610, 327.1237	1.33	Data base
**55**	16.94	333.1701	0.3	Taichunin B	$C_{19}H_{26}O_5$	274.1579, 189.0790	0.11	Data base
**Unknowns**							47.94	-

Table S5: ^{1}H, ^{13}C NMR (DMSO-d_6, 600 MHz and 151 MHz) except **1** (400 MHz and 100.6 MHz) of compounds **1**, **2**, **3A** and **3B**.

	**1**		**2**		**3A**		**3B**	
**Position**	δ_H**	δ_C	δ_H**	δ_C	δ_H**	δ_C	δ_H**	δ_C
**1**	-	161.8	-	156.5	-	161.7	-	155.5
**2**	7.29 (1H, d, *J*= 8.0)	124.9	-	138.8	7.44 (1H, d, *J*= 1.2)	119.1	-	137.6
**3**	7.74 (1H, t)	137.9	7.27 (1H, s)	129.1	-	146.6	7.28 (1H, s)	129.4
**4**	7.60 (1H, d, *J*= 4.0)	119.8	-	155.7	7.14 (1H, s)	124.2	-	156.0
**5**	7.60 (1H, d, *J*= 4.0)	119.8	-	168.9	7.22 (1H, d, *J*= 2.4)	107.0	-	163.5
**6**	7.74 (1H, t)	137.9	6.41 (1H, s)	107.4	-	164.5	6.89 (1H, d, *J*= 1.8)	105.4
**7**	7.29 (1H, d, *J*= 8.0)	124.9	-	164.9	6.86 (1H, d, *J*= 2.4)	105.0	-	164.5
**8**	-	161.8	7.07 (1H, s)	108.2	-	163.5	7.32 (1H, d, *J*= 1.8)	106.6
**9**	-	192.5	-	187.0	-	186.3	-	187.0
**10**	-	181.8	-	186.2	-	182.4	-	184.9
**11**	-	133.7	-	110.6	-	136.8	-	112.9
**12**	-	116.4	-	134.6	-	112.6	-	136.4
**13**	-	116.4	-	111.8	-	114.5	-	111.2
**14**	-	133.7	-	110.6	-	132.1	-	111.6
**2-CH_3**	-	-	2.26 (3H, s)	15.8	-	-	2.28 (3H, s)	15.7
**3-CH_3**	-	-	-	-	2.40 (3H, s)	21.4	-	-
**8-OCH_3**	-	-	-	-	3.92 (3H, s)	56.3	-	-
**5-OCH_3**	-	-	-	-	-	-	3.92 (3H, s)	56.4
**1-OH**	11.82 (1H, s)	-	13.18 (1H, s)	-	13.10 (1H, s)	-	13.57 (1H, s)	-
**4-OH**	-	-	13.33 (1H, s)	-	-	-	13.26 (1H, s)	-
**5-OH**	-	-	13.45 (1H, s)	-	-	-	-	-

**8-OH**	11.82 (1H, s)	-	-	-	-	-	-	-

** δ_H (integration, multiplicities, *J* in Hz

Table S6: ^{1}H, ^{13}C NMR (DMSO-d_6, 600 MHz and 151 MHz) of **compound 4, 5A and 5B.**

	**4**		**5B**		**5A**	
**Position**	δ_H**	δ_C	δ_H**	δ_C	δ_H**	δ_C
**1**	-	161.7	-	164.4	-	164.4
**2**	7.21 (1H, d, *J*=1.2)	120.9	-	130.7	-	130.7
**3**	-	151.3	-	136.7	-	136.7
**4**	7.58 (1H, d, *J*=1.2)	115.8	7.31 (1H, s)	118.6	7.50 (1H, s)	119.2
**5**	7.22 (1H, d, *J*=2.4)	107.5	7.08 (1H, d, *J*= 1.8)	107.0	7.23 (1H, d, *J*= 1.8)	107.0
**6**	-	165.3	-	165.0	-	165.0
**7**	6.86 (1H, d, *J*= 2.4)	105.1	6.59 (1H, d, *J*= 1.8)	108.2	6.89 (1H, d, *J*= 1.8)	105.1
**8**	-	163.5	-	164.6	-	163.5
**9**	-	186.2	-	189.2	-	185.5
**10**	-	182.5	-	182.3	-	182.4
**11**	-	136.8	-	133.5	-	131.7
**12**	-	112.3	-	109.9	-	115.5
**13**	-	115.2	-	119.2	-	112.6
**14**	-	132.2	-	134.6	-	134.7
**2-CH_3**	-	-	-	-	-	-
**3-CH_3**	-	-	2.56 (3H, s)	22.0	2.38 (3H, s)	19.8
**8-OCH_3**	3.90 (3H, s)	56.3	-	-	3.92 (3H, s)	56.4
**5-OCH_3**	-	-	-	-	-	-
**3-CH_2**	4.58 (2H, s)	62.0	-	-	-	-
**1-OH**	13.35 (1H, s)	-	13.09 (1H, s)	-	12.74 (1H, s)	-
**8-OH**	-	-	13.33 (1H, s)	-	-	-
**3- OH**	3.87 (1H, s)	-	-	-	-	-
**2-COOH**	-	-	11.14 (1H, s)	167.5	11.36 (1H, s)	167.5

** δ_H (integration, multiplicities, *J* in Hz).

Table S7: Effect of Am-10 on cell cycle distribution and apoptosis of breast cancer cell line MCF-7.

Sample	A) Cell cycle distribution (%)			
		%G0-G1	%S	%G2/M
Am-10/MCF-7		61.22 ± 5.51	36.92 ± 2.95	1.86 ± 0.09
Untreated MCF-		52.94 ± 3.18	28.11 ± 1.97	18.95 ± 1.33
p-value		0.0171	0.0115	<0.0001
	B) Detection of Apoptosis and Necrosis (%)			
	Apoptosis			Necrosis
	Total apoptosis	Early	Late	
Am-10/MCF-7	23.26± 1.04	17.92 ± 0.72	5.34 ± 0.32	4.07 ± 0.20
Untreated MCF-	0.85±0.04	0.66 ± 0.05	0.19 ± 0.01	2.27 ± 0.16
p-value	<0.0001	<0.0001	<0.0001	<0.0001
Fold change	27.36-fold	27.15	28.10	1.79

*Untreated breast cancer cell line MCF-7 was used as the negative control. Values are presented as mean ± SD ($n = 3$ independent biological replicates). Statistical comparisons between Am-10-treated and untreated MCF-7 cells were performed using two-way ANOVA followed by Tukey's multiple-comparisons test. Statistical significance was set at $p < 0.05$.

Table S8: Docking results of isolated compounds (kJ/mol) to apoptosis-related proteins *Bax* (PDB ID:4BD6), *Bcl-2* (PDB ID: 6O0K) and *p53* (PDB ID: 8DC6).

Compound		MolDock Score	H. bond	LE1	Docking Score	Amino acids involved in H-bonding
1	*Bax*	-49.1347	-2.58133	-2.72971	-50.7226	Ile 19
	Bcl-2	-70.2894	-3.55364	-3.90497	-68.1883	Ser 105, Asp 111
	p53	-73.9132	1.45862	-4.10629	-73.8057	Thr 155, Cys 220
2	*Bax*	-61.0899	-4.50903	-2.90904	-63.1871	Ile 19, Pro 13
	Bcl-2	-66.7591	-3.71757	-3.17901	-72.3529	Val 133, Ala 149
	p53	-75.2373	-11.0599	-3.58273	-85.0368	Glu 221, Ser 227, Ser 229, Trp 146
3A	*Bax*	-69.6832	-2.5	-3.31825	-69.9673	Ile 19
	Bcl-2	-86.4638	-2.81681	-4.11732	-88.3132	Tyr 108, Val 133
	p53	-74.2696	-6.00123	-3.53665	-82.7194	Cys 220, Trp 146, Glu 221
3B	*Bax*	-67.6396	-2.5	-3.07453	-65.3806	Ile 19
	Bcl-2	-71.2698	-3.00301	-3.23953	-73.2529	Val 133, Asp 111
	p53	-77.3884	-11.5135	-3.51766	-86.6417	Cys 220,Trp 146, Ser 227, Ser 229, Glu 221
4	*Bax*	-76.4211	-5	-3.47369	-80.3281	Ile 19, Gln 18
	Bcl-2	-89.6944	-5.32575	-4.07702	-92.9328	Val 133, Glu 136, Tyr 108
	p53	-80.856	-8.48917	-3.67527	-89.9813	Ser 227, Trp 146, Cys 220, Glu 221
	Bax	-74.6385	-4.51274	-3.24515	-76.9524	Ile 19, Pro 13
5B	*Bcl-2*	-72.2217	-2.73861	-3.14007	-75.2944	Ser 105, Val 133
	p53	-76.3484	-6.61866	-3.3195	-76.8388	Ser 227, Glu 224, Pro 222, Glu 221, Cys 220
	Bax	-73.2483	-2.5	-3.05201	-77.9587	Pro 13
5A	*Bcl-2*	-91.1892	-2.5000	-3.79955	-91.9478	Tyr 108, Ser 105
	p53	-107.548	-9.44189	-4.48115	-105.695	Thr 155, Cys 220, Asp 228,Val 147, Leu 145
Methotrexate	*Bax*	-105.865	-2.65084	-3.20803	-119.317	Thr 22,Ser 55,Thr 56, Lys 21
	Bcl-2	-110.596	-7.19698	-3.35138	-130.473	Arg 146, Asp 111
	p53	-135.504	-9.27586	-4.10618	-142.049	Gln 136,Asp 228, Leu 145

[a]MolDock score: The docking energy score. [b]H. Bond: hydrogen bonding energy. . [c]LE 1 (Ligand efficiency 1): calculated by dividing MolDock score by heavy atoms count.

Table S9: Physicochemical properties methotrexate and compounds 1-5 for prediction of drug-likeness and oral bioavailability.

Compound	M.W[a] g/mol	Rotatable bonds	HBA[b]	HBD [c]	TPSA (Å²) [d]	WLogP [e]
**1**	240.21	0	4	2	74.60	1.87
**2**	286.24	0	6	4	115.06	1.59
**3A**	284.26	1	5	2	83.83	2.19
**3B**	300.26	1	6	3	104.06	1.90
**4**	300.26	2	6	3	104.06	1.22
**5B**	314.25	1	7	4	132.13	1.59
**5A**	328.27	2	7	3	121.13	1.89
**Methotrexate**	454.44	10	9	5	210.54	0.13

[a] M.W: Molecular weight c HBA: Hydrogen bond acceptors
[b] HBA:hydrogen bond acceptor
[c] HBD: Hydrogen bond donor
[d]TPSA: Topological polar surface area
[e] WLOGP: Represents partition coefficient

Table S10: *In silico* toxicity prediction of isolated anthraquinones using pkCSM.

Compound	AMES Mutagenicity	Hepatotoxicity	hERG Inhibition hERG I/II	Oral Rat Acute Toxicity (LD50, mol/kg)	Chronic Toxicity (LOAEL, log mg/kg/day)
**1**	+ve	-ve	-ve	1.979	2.073
**2**	+ve	-ve	-ve	1.996	2.089
**3A**	+ve	-ve	-ve	2.009	1.571
**3B**	+ve	-ve	-ve	2.042	1.681
**4**	+ve	-ve	-ve	1.820	1.738
**5B**	**-ve**	**-ve**	**-ve**	**2.890**	2.487
**5A**	**-ve**	**-ve**	**-ve**	**2.887**	1.872
**Methotrexate**	+ve	+ve	+ve	+ve	Significant chronic toxicity

AMES, mutagenicity prediction; hERG, human ether-à-go-go-related gene potassium channel; hERG I/II, class I and class II potassium channels; LD_{50}, median lethal dose (mol/kg); LOAEL, lowest observed adverse effect level (log mg/kg/day).

References

1 E. H. Anouar, C. P. Osman, J.-F. F. Weber and N. H. Ismail, *Springerplus*, 2014, **3**, 233.

2 I. Vidal, J. A. Torres-Vargas, J. M. Sanchez, M. Trigal, M. Garcia-Caballero, M. A. Medina and A. R. Quesada, *Antioxidants*, 2023, **12**, 1101.

3 S. Rossi, C. Tabolacci, A. Lentini, B. Provenzano, F. Carlomosti, S. Frezzotti and S. Beninati, *Anticancer Res.*, 2010, **30**, 445–449.

4 B. S. M. M. Al Kazman and J. M. Prieto, *Nutraceuticals*, 2021, **1**, 13–30.

5 Z. Xie, X. Fan, A. S. Sallam, W. Dong, Y. Sun, X. Zeng and Z. Liu, *Food Chem.*, 2025, **474**, 143104.

6 D.-L. Li, X.-M. Li and B.-G. Wang, *J. Microbiol. Biotechnol.*, 2009, **19**, 675–680.

7 C. Yu, S. Lin, X. Ma, J. Zhang, C. Wu, B. Ma, H. Wang and Y. Pei, *Nat. Prod. Res.*, 2022, **36**, 5484–5489.

8 W. W. M. Zin, S. Buttachon, T. Dethoup, J. A. Pereira, L. Gales, Â. Inácio, P. M. Costa, M. Lee, N. Sekeroglu, A. M. S. Silva, M. M. M. Pinto and A. Kijjoa, *Phytochemistry*, 2017, **141**, 86–97.

9 L. Guo, F. Zhang, X. Wang, H. Chen, Q. Wang, J. Guo, X. Cao and L. Wang, *3 Biotech*, 2019, **9**, 14.

10 W. Wang, T. Zhu, H. Tao, Z. Lu, Y. Fang, Q. Gu and W. Zhu, *J. Antibiot. (Tokyo)*, 2007, **60**, 603–607.

11 P. Jetha, D. Mojzita, N. Maiorova, J. C. de Ruijter, H. Maaheimo, S. Hilditch, G. Peddinti, S. Castillo, M. Toivari and M. Penttilä, *Biotechnol. Biofuels Bioprod.*, 2025, **18**, 25.

12 X. Yang, M.-C. Kang, Y. Li, E.-A. Kim, S.-M. Kang and Y.-J. Jeon, *J. Microbiol. Biotechnol.*, 2014, **24**, 1346–1353.

13 H. Yang, W. Lin, J. Zhang, W. Lin, P. Xu, J. Li and X. Ling, *J. Chromatogr. B*, 2014, **959**, 49–54.

14 W. Hou, J. Ling, Y. Xu, K. Li and F. Wang, *MethodsX*, 2021, **8**, 101470.

15 N. Sinosaki, A. P. P. Tonin, M. A. S. Ribeiro, C. B. Poliseli, S. B. Roberto, R. da Silveira, J. V. Visentainer, O. O. Santos and E. C. Meurer, *J. Braz. Chem. Soc.*, 2020, 31, 402–408.

16 C. Zhan, A. Xiong, D. Shen, L. Yang and Z. Wang, *Molecules*, 2016, **21**, 631.
